# Differential gene screening and bioinformatics analysis of epidermal stem cells and dermal fibroblasts during skin aging

**DOI:** 10.1038/s41598-022-16314-z

**Published:** 2022-07-14

**Authors:** Weisheng Hu, Yuan Jing, Qingqian Yu, Ning Huang

**Affiliations:** 1 The Second Affiliated Hospital of Fujian University of Traditional Chinese Medicine, Fuzhou, 350003 China; 2 College of Acupuncture, Fujian University of Traditional Chinese Medicine, Fuzhou, 350122 China; 3 College of Traditional Chinese Medicine, Beijing University of Traditional Chinese Medicine, Beijing, 100105 China; 4 Key Laboratory of Dermatology in Integrated Traditional Chinese and Western Medicine, The Second Affiliated Hospital of Fujian University of Traditional Chinese Medicine, Fuzhou, 350003 China

**Keywords:** Computational biology and bioinformatics, Molecular biology

## Abstract

To explore the differentially expressed genes (DEGs) and potential therapeutic targets of skin aging in GEO database by bioinformatics methods. Dermal fibroblasts and skin aging related data sets GSE110978 and GSE117763 were downloaded from GEO database, and epidermal stem cells and skin aging related data sets GSE137176 were downloaded. GEO2R was used to screen DEGs of candidate samples from the three microarrays, GO function analysis and KEGG pathway analysis were performed. Protein interaction network was constructed using String database, and hub gene was obtained by Cytoscape. NetworkAnalys was used to analyze the coregulatory network of DEGs and MicroRNA (miRNA), interaction with TF, and protein-chemical interactions of DEGs. Finally, DSigDB was used to determine candidate drugs for DEGs. Six DEGs were obtained. It mainly involves the cytological processes such as response to metal ion, and is enriched in mineral absorption and other signal pathways. Ten genes were screened by PPI analysis. Gene-miRNA coregulatory network found that Peg3 and mmu-miR-1931 in DEGs were related to each other, and Cybrd1 was related to mmu-miR-290a-5p and mmu-miR-3082-5p. TF-gene interactions found that the transcription factor UBTF co-regulated two genes, Arhgap24 and Mpzl1. Protein-chemical Interactions analysis and identification of candidate drugs show results for candidate drugs. Try to explore the mechanism of hub gene action in skin aging progression, and to discover the key signaling pathways leading to skin aging, which may be a high risk of skin aging.

## Introduction

Aging is a complex and continuous biological process in the human body and an inevitable trend of human metabolism. With the increase in the number of aging cells, the skin shows a very obvious correlation between aging and the passage of time. Skin aging is characterized by reduced thickness^1^ and changes in biomechanical properties^2^, and clinical manifestations include dryness, wrinkles, sagging, blemishes, and all other signs of skin aging observed in the aging process^3^. Skin aging does not shorten life expectancy, but can significantly reduce skin condition and affect psychosocial status.

Skin aging is the product of a variety of pathogenic mechanisms and pathways. Although people still know little about the basic mechanisms and pathways of skin aging, more and more evidence suggests that one of the most important factors affecting the in vivo aging is the gradual degradation or loss of functions at the cellular level^4^.Epidermal stem cells and dermal fibroblasts play a critical role in the multi-causal process of skin aging.With the increase of age, the decrease in the growth potential of skin dermal fibroblasts has long been observed^5^.Dermal fibroblasts play an important part in maintaining the integrity of the dermis structure^6^. During aging, the proliferation and metabolic activity of dermal fibroblasts decreased, and the structure changed characteristically, including thinning of the dermis, flattening of the dermis-epidermal junction, and loss of normal reticular ridge.This may be because the relative lack of adult dermal fibroblasts during aging is not sufficient to maintain the structural integrity of the extracellular matrix, that is associated with changes in the composition of endodermal fibroblast subtypes with age^7,8^. At the same time, another notable feature of skin aging is the depletion or imbalance of epidermal stem cells. Stem cell senescence marks the development of aging and aging related diseases. Epidermal stem cells are the progenitor cells of various epidermal cells. They derive from the embryonic ectoderm and have the ability of bidirectional differentiation. On the one hand, it can migrate down and differentiate into epidermal basal layer, and then generate hair follicles. On the other hand, they migrate upward and eventually differentiate into various epidermal cells^9^. Epidermal stem cells constantly renew their surface throughout the life cycle of animals and recapitualtion after wound injury, suggesting the existence of epidermal stem cells to ensure that these key functions are performed^10^.Skin aging is caused by the impaired mobilization of stem cells or the decrease in the number of stem cells capable of responding to proliferation signals, which increases skin inflammation and is difficult to maintain homeostasis in vivo, and slows down the repair of skin tissue after external injury^11^.At the same time, miRNAs play an important role in regulating the balance between skin cell proliferation and replicative senescence. MiRNA mediates the function of dermal fibroblasts and is associated with decreased expression of transmembrane receptors (such as integrin) and ECM components (such as collagen and elastin) in aging skin fibroblasts^12^.However, there is the absence of reliable research on the role of dermal fibroblasts and epidermal stem cell-related genes and miRNAs in skin aging and anti-aging. Various aspects of these related genes remain to be further studied.

Bioinformatics analysis is to use the computer for high-throughput cell biology technology to produce all kinds of data searching, sorting, analysis, storage, in nucleic acid and protein level analysis, to explore the structure and function of skin aging related biological macromolecular information, provide us with different genes play a role in the process of skin aging progress more information related pathways and comments. This study based on the GEO database, screening of epidermal stem cells and dermal fibroblasts of gene chip data, using bioinformatics analysis tools of epidermal stem cells and dermal fibroblasts genomics data analysis, screening epidermal stem cells and dermal fibroblasts in the DEGs in the process of aging.

## Materials and methods

### Data acquisition and identification of DEGs

In GEO database (https://www.ncbi.nlm.nih.gov/geo/)^13^ in search of skin aging related data expression spectrum, 3 microarray data set (GSE110978 GSE117763 GSE137176) are included in the study, the chip dataset contains mice data grouped by age. GSE110978 and GSE117763 are derived from GPL11180 Affymetrix HT MG-430 PM Array Plate platform, and GSE137176 from GPL6246 Affymetrix Mouse Gene platform. The inclusion criteria for this study were to select dermal fibroblasts or epidermal stem cells isolated from the skin of mice of different ages as samples, and divide the mice into young and old groups according to their age. The GSE110978 dataset consisted of 8 specimens, including dermal fibroblasts from 4 young (2 months old) and 4 old (18 months old) mice. The GSE117763 dataset consisted of 8 specimens, including dermal fibroblasts from 4 young (2 months old) and 4 old (18 months old) mice. The GSE137176 dataset included epidermal stem cells from 3 young (1 month old) and 3 old (12 months old) mice.

### Screening of DEGs and correlation analysis

Online analysis tool geo2r using geo database (https://www.nvbi.nlm.nih.gov/geo/Geo2r/) to screen the differential genes. The limma package^14^ of the R language is used to screen DEGs. The screening criteria are FDR < 0.05 and|log2 (FC) |> 1. In order to intuitively reflect the gene expression, all genes in the two data sets are drawn into volcanic maps respectively. To better understand the DEGs, online software Morpheus (https://software.broadinstitute.org/morpheus/) was used to draw heat maps of the first 20 up-regulated and the first 20 down-regulated genes in the two datasets, and according to the screening criteria, The software Draw Venn Diagram (http://bioinformatics.psb.ugent.be/webtools/Venn/) was used to Draw the Venn Diagram of the two groups of differential genes screened from the two data sets to obtain the common DEGs.

### Functional enrichment analysis of DEGs

The above common DEGs were subjected to GO functional annotation and KEGG pathway analysis using DAVID (the Database for Annotation, Visualization and Integrated Discovery) database (https://david.ncifcrf.gov/)^15^. The DAVID database integrates biological data and analysis tools to provide researchers with the information annotation function of genes and proteins. GO is a bioinformatics tool for analyzing and annotating biological processes of genes. GO consists of three parts: Molecular Function, Biological Process, and Cellular Component. KEGG analysis can help people analyze signaling pathways from large-scale molecular data sets generated by high-throughput experimental techniques, and include the process by which multiple proteins interact and co-regulate cellular function and metabolic activity. Adjusted *P* < 0.05 for threshold screening, visualized, and plotted GO and KEGG results as chord diagrams utilizing R language.

### PPI network construction and module analysis

Protein–protein interaction (PPI) refers to the dynamic and complex network of interactions that arise between two or more proteins. The String Database (http://string-db.org)^16^ is a common database for PPI analysis, providing experimental and predictive interaction information. To obtain proteins that interact directly or indirectly with DEGs, DEGs was imported into the String database, the trust value was set to > 0.4, mice were selected for species selection, protein interaction network was formed, and data was imported into GeneMANIA (http://genemania.org/), A database that generates gene function hypotheses, analyzes gene lists, and analyzes gene priorities, and visualizes the results. Cytoscape^17^ is a software platform for visualizing molecular interaction networks. Cytoscape software was utilized to visualize the results. At the same time, the MCODE plug-in of Cytoscape software was used to determine the most important modules in the PPI network of the two data sets. The threshold score was set to ≥ 4.5, and the important modules with the highest score were selected for visualization display.

### Expression level analysis of hub genes identification and module analysis

CytoHubba plug-in in Cystoscape^18^ was used to screen hub genes, and the MCC algorithm in CytoHubba plug-in was used to screen the top 10 genes with the largest correlation in the current research network as hub genes. These top 10 genes are the hub genes associated with epidermal stem cells and dermal fibroblasts during skin aging. And then by using ImageG the boxplot tools (http://www.ehbio.com/ImageGP/), old and young group of GSE137176 data set of differences in gene expression between the hub for visualization, draw boxplot. GO functional annotation and KEGG pathway analysis were performed for the above 10 genes using DAVID database.

### Gene—micrornas coregulatory network

The interaction information between GENE and miRNA was retrieved from miRTarBasev8.0 information base. Data collected from Comprehensive satisfaction validated mirNA-gene interaction data collected from miRTarBase This database is helpful to detect the association between DEGs gene and miRNA. The Gene-mirNA coordination network was visualized using NetworkAnalyst. NetworkAnalyst NetworkAnalyst (https://www.NetworkAnalyst.ca/) is a comprehensive network platform, is used to identify transcription factor genes and the interaction between DEGs. At the same time, the miRTarBase database in MiRwalk(http://mirwalk.umm.uni-heidelberg.de/) database was used to input 6 selected DEGs to conduct prediction analysis of interaction between miRNA and mRNA. The results into the miRNA—mRNA interaction table.

### TF—gene interaction

NetworkAnalyst is also used to identify the interaction between transcription factors(TF) and DEGs, interact identified common DEGs with transcription factors, evaluate the results of transcription factors in functional pathways and gene expression levels, and visualize the results. NetworkAnalyst ENCODE (https://www.encodeproject.org/) in the database is used to search the TF and gene interaction network.

### Protein—chemical interactions

The data is based on The Comparative Toxicogenomics Database Interactions (CTD) (Downloaded on Nov. 2016), Modules regulating protein-chemical Interactions were collected from the Comparative Toxicogenomics Database Interactions information bank and visualized using NetworkAnalyst. To determine the biometrics and functions of genes, leading to valid drug hypotheses.

### Identification of candidate drugs

The Drug Molecule Identification is used to find the key components of a Drug. Using the Drug Signatures Database (DSigDB) to find Drug candidates related to DEGs. The access of the DSigDB database is acquired through Enrichr (https://amp.pharm.mssm.edu/Enrichr/) platform. Enrichr is mostly used as an enrichment analysis platform that represents numerous visualization details on collective functions for the genes that are provided as input^19^.

### Ethical consent

This article is based on public data and does not address patients' ethical and informed consent.

## Results

### Identification of DEGs and correlation analysis

**Figure 1:**
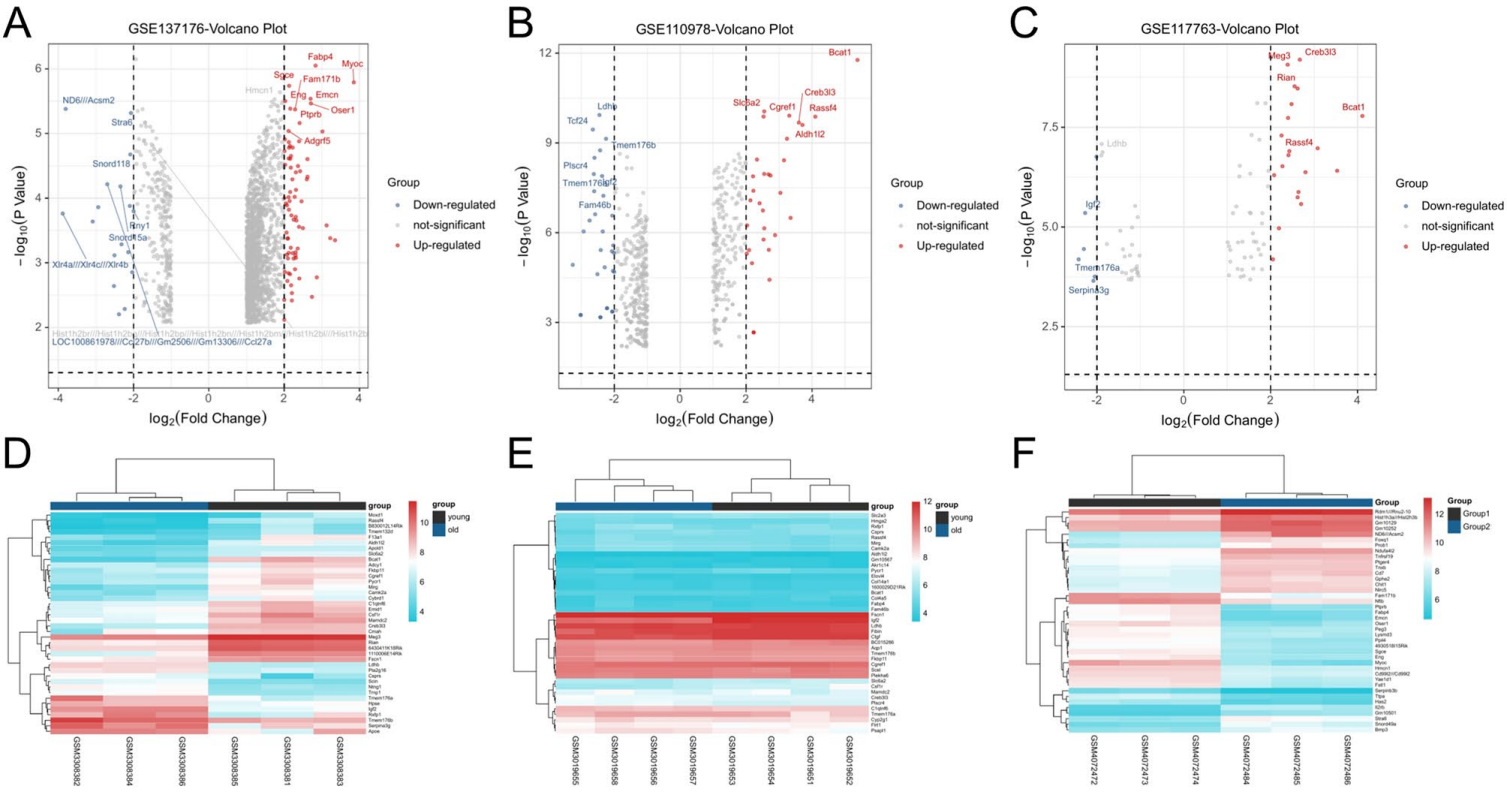
Identification and analysis of DEGs: (**A**) Volcano map of DEGs in the GSE137176 dataset. (**B**) Volcano map of DEGs in the GSE110978 dataset. (**C**) Volcano map of DEGs in the GSE117763 dataset. (**D**) Heat map of DEGs in GSE137176 dataset. (**E**) Heat map of DEGs in the GSE110978 dataset. (**F**) Heat map of DEGs in the GSE117763 dataset. In all images of figure, red represents up-regulated genes and blue represents down-regulated genes.

After screening the inclusion conditions, GSE110978 and GSE117763 data sets were used for the identification of DEGs in dermal fibroblasts from young and old mice. GSE110978 obtained 532 DEGs genes, including 153 up-regulated genes and 199 down-regulated genes. Sixty DEGs genes were obtained from GSE117763, including 33 up-regulated genes and 27 down-regulated genes. The GSE137176 dataset was used to identify epidermal stem cell genes in young and old mice. A total of 1519 DEGs were identified, of which 1280 genes were up-regulated and 239 genes were down-regulated. All genes in the three data sets were plotted into volcanic maps (Fig. 1A–C), with blue representing up-regulation and red representing down-regulation. In order to better understand the distribution of DEGs, online software Morpheus was used to draw heat maps of the top 20 up-regulated and top 20 down-regulated genes screened out from the three data sets, respectively, and the results showed that mice of different age groups could be well distinguished, as shown in Fig. 1D–F.

### Screening of DEGs

**Figure 2:**
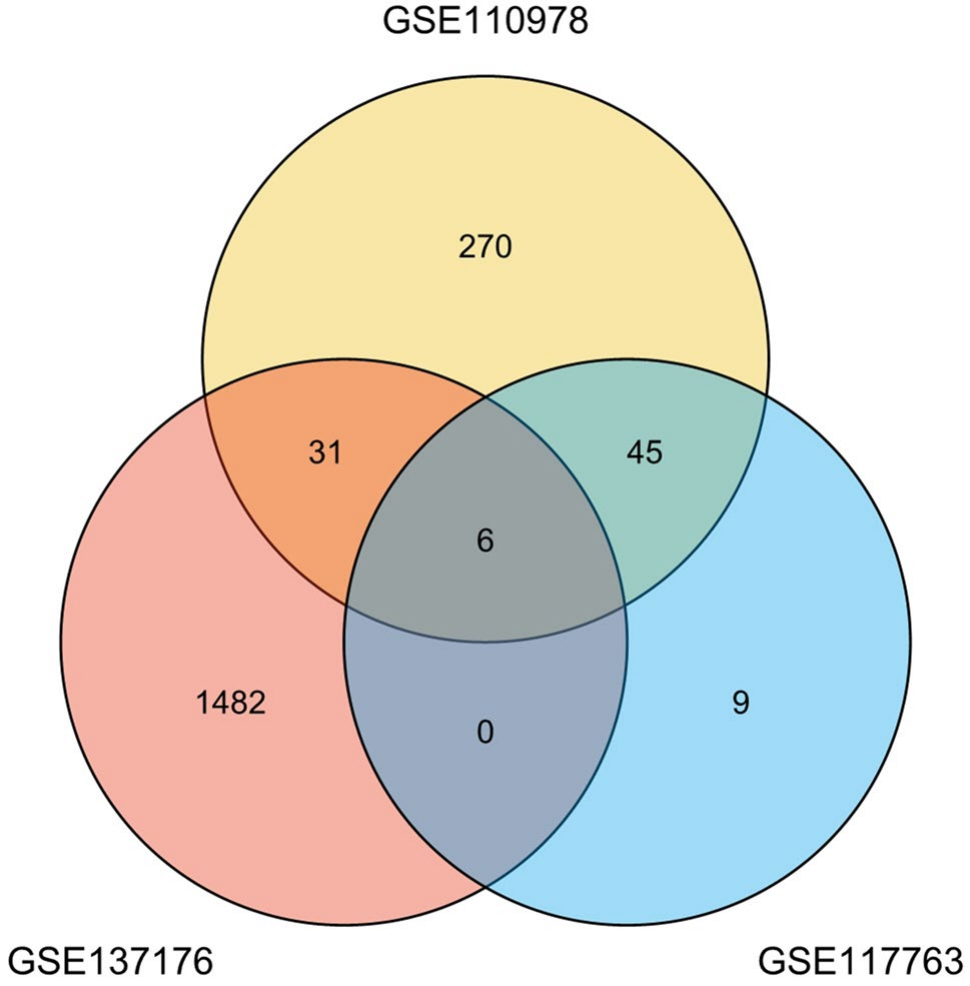
Venn diagram: Venn plots of DEGs in GSE137176, GSE110978, and GSE117763 data sets, and the overlapped part represents the common DEGs in the three data sets, 6 in total.

According to the screening criteria, the DEGs screened out from the three sets of data were drawn by the online software Draw Venn Diagram to obtain the common DEGs, as shown in Fig. 2. A total of 592 dermal fibroblast genes collected by GSE110978 and GSE117763 were compared with 1519 epidermal stem cell genes collected by GSE137176. Six common DEGs (Arhgap24, Peg3, Mpzl1, Col14a1, Cybrd1 and Trf) were identified. Among the 6 genes, Arhgap24, Peg3, Mpzl1, Col14a1 and Cybrd1 were up-regulated in three data sets, while Trf gene was up-regulated in dermal fibroblast gene data sets (GSE110978 and GSE117763). However, Trf gene was down-regulated in the epidermal stem cell gene dataset (GSE137176).

### Enrichment analysis of common DEGs

**Figure 3:**
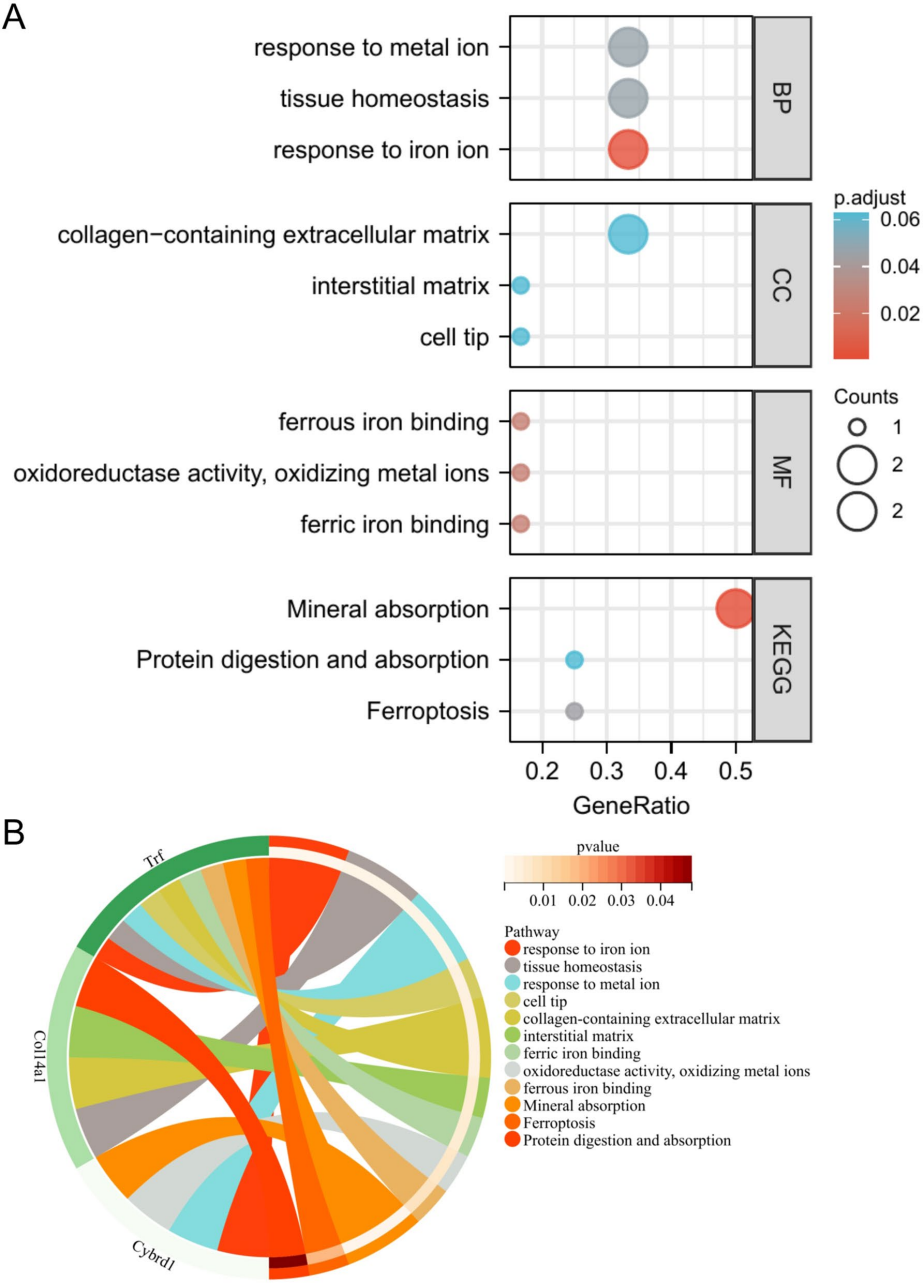
GO and KEGG analysis results of genes: (**A**) GO biological, GO cell, GO molecular function enrichment results, and KEGG pathway enrichment results. (**B**) GO and KEGG analysis of string.

Using the DAVID online database, the online analysis of the six common DEGs (Arhgap24, Peg3, Mpzl1, Col14a1, Cybrd1, Trf) obtained from the screening obtained the enrichment information of GO and KEGG. The "BP" enriched by common DEGs mainly include response to metal ion, tissue homeostasis, and response to iron ion (Fig. 3A); in terms of "CC", common DEGs are mainly enriched in collagen-containing extracellular matrix, interstitial matrix, cell tip region (Fig. 3A). The "MF" aspect suggests that DEGs are mainly related to the processes of ferrous iron binding, oxidoreductase activity, oxidizing metal ions, and ferric iron binding (Fig. 3A). In the enrichment analysis of the KEGG signaling pathway, the information obtained in Fig. 3A shows that the Mineral absorption, Protein digestion and absorption, and Ferroptosis regions (Fig. 3A) interact with genes in the KEGG signaling pathway database. The results obtained from the enrichment analysis of the GO and KEGG signaling pathways were plotted as a chord diagram (Fig. 3B). The distribution of the 12 enriched pathways can be observed. The figure shows three genes, Col14a1, Trf, and Cybrd1.

### PPI network construction and module analysis

**Figure 4:**
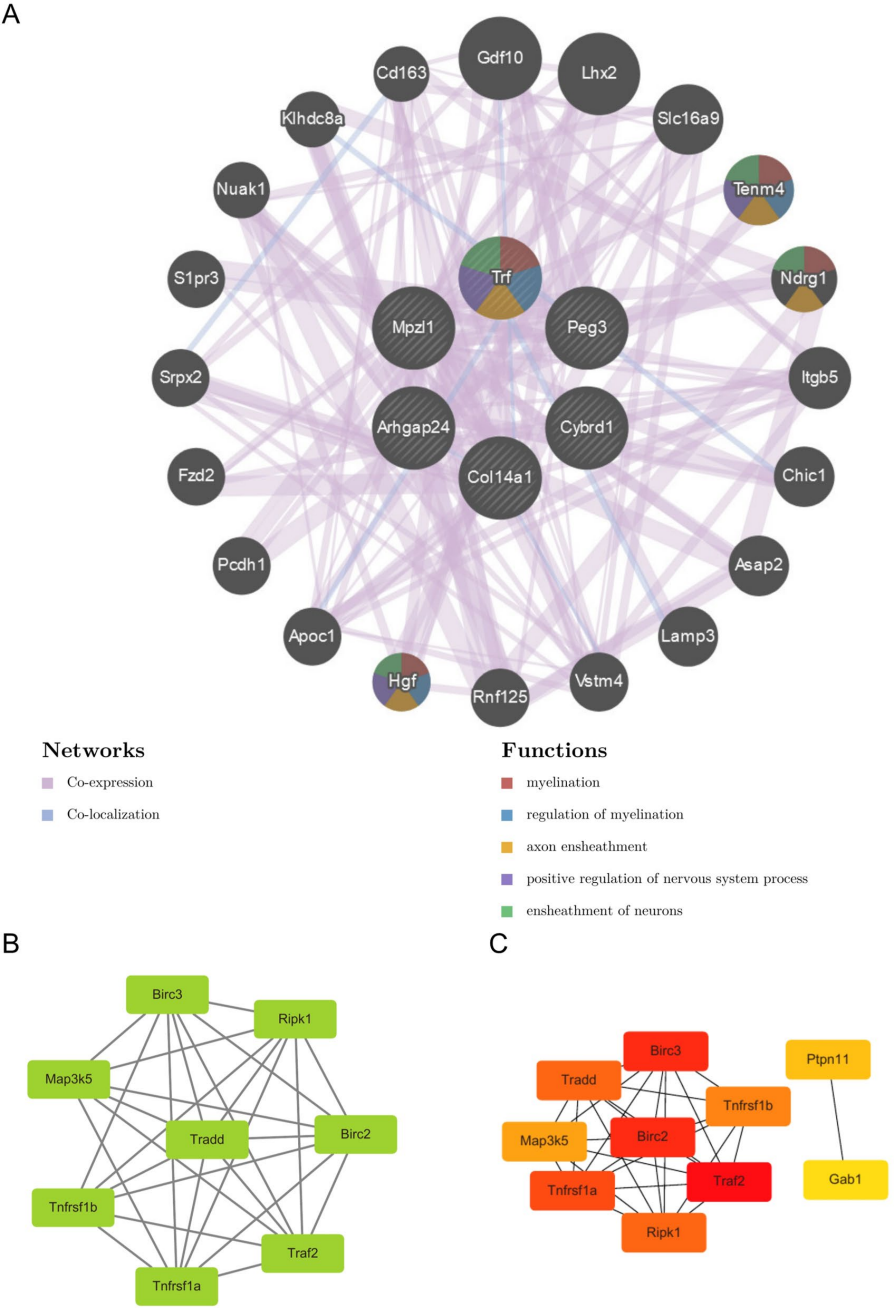
PPI network: (**A**) Common gene PPI network. (**B**) Key protein expression module (**C**) PPI networks encoded by key gene interactions.

The String tool was used to construct the PPI network of the common DEGs, the species was selected as mouse, and the obtained results were imported into the Cytoscape software, and the PPI network was visually analyzed and screened. The PPI network was plotted using GeneMANIA, see Fig. 4A, showing that the common DEGs contain 26 nodes and 142 edges. Using the MCODE plug-in of Cytoscape software to analyze the most important clustering modules in the PPI network, the screening method is the threshold score ≥ 4.5, and the key module results can be obtained. The most important modules with the highest scores are selected for presentation, and new clustering networks are created, forming Fig. 4B. The results show that it contains 8 nodes and 29 edges, and the genes of key modules include: Birc3, Tradd, Birc2, Tnfrsf1b, Tnfrsf1a, Ripk1, Traf2, Map3k5.

### Expression level analysis of hub genes

**Figure 5:**
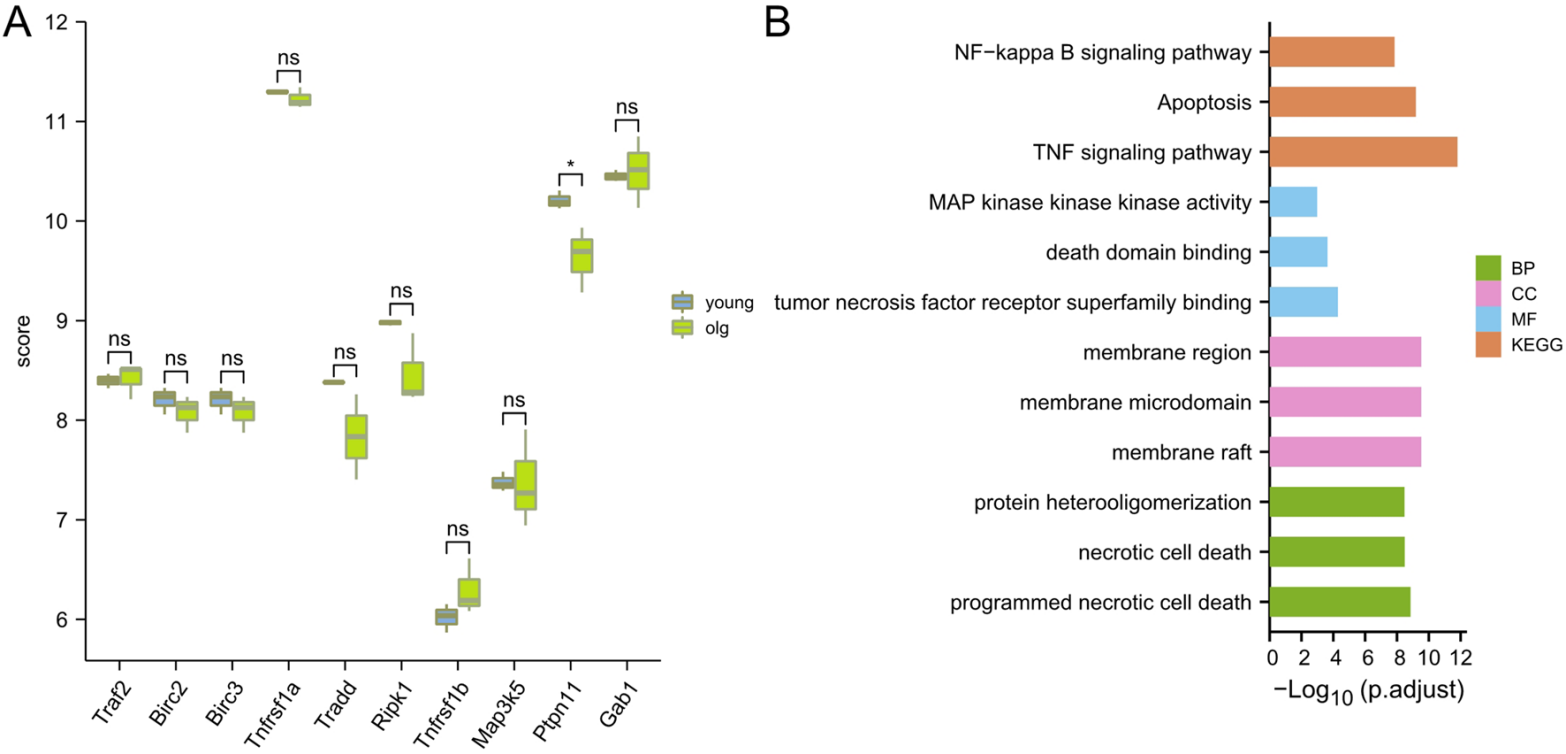
Analysis of hub genes: (**A**) Box diagram of the distribution of 10 hub genes in the GSE137176 dataset; (**B**) GO and KEGG analysis of 10 hub genes.

The Cytohubba plugin was used to sort the hub genes according to the degree value, and the top 10 hub genes identified were Birc3, Tradd, Map3k5, Birc2, Tnfrsf1b, Tnfrsf1a, Ripk1, Traf2, Ptpn11, and Gab1. In the PPI network, the interactions of hub proteins with other proteins are shown in Fig. 4C. The network consists of 53 nodes and 378 edges. By using the ImageGP boxplot tool, the expression differences of 10 genes in the old and young groups in the GSE137176 dataset were visualized. The results are shown in Fig. 5A. The overall distribution and data of continuous variables, the tightness of the grouping and whether the data distribution is skewed can be drawn from the boxplot. And the 10 genes were analyzed by GO and KEGG, and the pathways related to the hub gene were shown. The results are shown in Fig. 5B.

### Gene—micrornas coregulatory network

**Figure 6:**
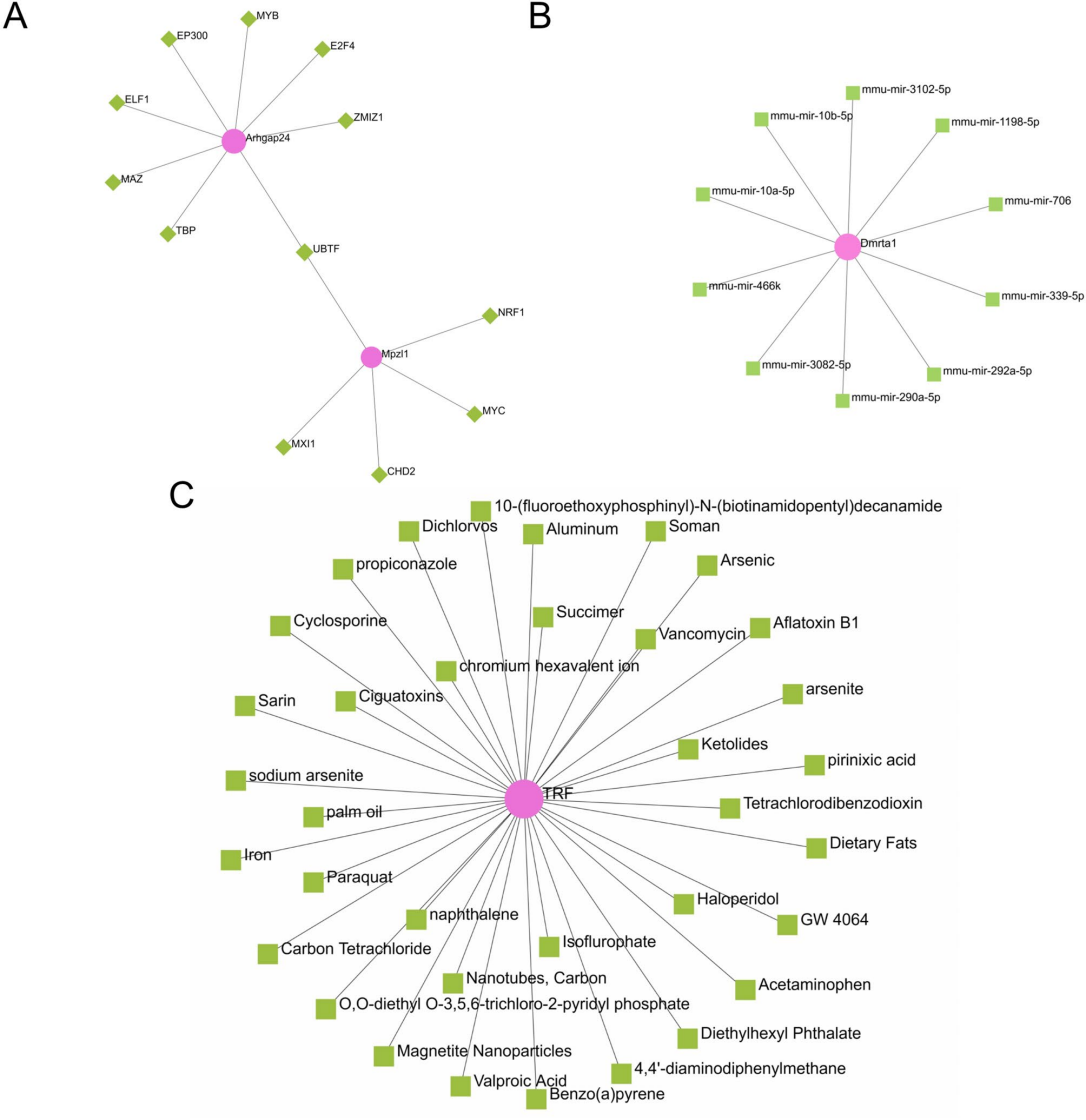
NetworkAnalyst analysis of common DEGs: (**A**) Gene-mirNA coregulatory network, pink represents genes and green represents mirnas. (**B**) Gene identification of TF-Gene Interactions. Pink represents genes and green represents transcription factors. (**C**) Protein-chemical Interactions of common DEGs. Pink represents genes and green represents drugs.

Gene-miRNA coordination networks were generated using NetworkAnalyst. Through the analysis of the Gene-miRNA co-regulatory network, a network diagram of the interaction between DEGs and miRNAs was generated. This interaction may be responsible for regulating the expression of DEGs. Figure 6A shows the synergistic regulatory network of Gene-miRNA, including 11 nodes and 10 edges. 10 miRNAs interact with 1 gene (Dmrta1) in common DEGs. At the same time, MiRwalk was used to predict and analyze the interaction of miRNA-mRNA, and a table of miRNA-mRNA interaction was drawn, which is shown in Table 1. Table 1 shows that Peg3 and Cybrd1 interact with 3 miRNAs in common DEGs, among which mmu-miR-1931 interacts with Peg3, Cybrd1 interacts with mmu-miR-290a-5p, mmu-miR-3082-5p connect.

**Table 1:** Prediction analysis of mirNA-mrna interactions of common DEGs.

miRNA	GeneSymbol	Binding Site	Au	Me	N Pairings	Mirtarbase
mmu-miR-1931	Peg3	57,865,815	0.56	− 6.51	19	MIRT596152
mmu-miR-290a-5p	Cybrd1	18,611,883	0.52	− 9.77	19	MIRT600998
mmu-miR-3082-5p	Cybrd1	27,132,726	0.57	− 15.56	12	MIRT600993

### TF—gene interactions

Collect transcription factor and gene interaction information using NetworkAnalyst. Tf-gene Interactions of common DEGs (Arhgap24, Peg3, Mpzl1, Col14a1, Cybrd1, and Trf) were identified, and the results were shown in Fig. 6B. The network showed links between 12 transcription factors and 2 common DEGs, with a total of 14 nodes and 13 edges. Arhgap24 is regulated by 8 transcription factors, and Mpzl1 by 5 transcription factors. Transcription factor UBTF co-regulates Arhgap24 and Mpzl1 genes. These 12 transcription factor regulate more than one common DEGs in the network, indicating a high degree of interaction between common DEGs and transcription factors.

### Protein—chemical Interactions

Use NetworkAnalyst software to generate a network of protein and chemical Interaction. Through the analysis of the Protein-chemica co-regulatory network, Fig. 6C shows the interaction of Protein and Chemica among common DEGs. This interaction may provide direction for chemical drugs that modulate proteins expressed by common DEGs. The network built for Protein-chemical consists of 1 node (Trf) and 36 edges. This shows that epidermal stem cells and dermal fibroblasts regulate Trf gene-related chemicals in genes.

### Identification of candidate drugs

The Enrichr platform is used to identify the relationship between drug molecules and six common DEGs. The data was collected from the DSigDB database. According to the P value and adjusted P value, the results of candidate drugs are generated. Table 2 indicates the DEGs candidate drugs from DSigDB database. Analysis showed that yohimbic acid PC3 UP was the drug molecule with most gene interactions, Combined score was 857.29. 4-TERT-Octylphenol CTD 00003436 ranked second. Because these signature drugs were found in six common DEGs, they represent related drugs that regulate epidermal stem cell senescence and dermal fibroblast senescence.

**Table 2:** Candidate drugs of DEGs in DSigDB database.

Index	Name	*P*-value	Adjusted *p*-value	Odds ratio	Combined score
1	Yohimbic acid PC3 UP	0.007179	0.2346	173.66	857.29
2	4-tert-Octylphenol CTD 00003436	0.01372	0.2346	88.66	380.25
3	4-nonylphenol CTD 00001904	0.02052	0.2346	58.61	227.75
4	Adenine CTD 00005317	0.02553	0.2347	46.84	171.83
5	Hydroxyurea CTD 00006132	0.02846	0.2347	41.89	149.11
6	Rofecoxib CTD 00003631	0.02934	0.2347	40.6	143.29
7	Solanine HL60 UP	0.0375	0.2728	31.54	103.54
8	Primaquine PC3 DOWN	0.009975	0.2346	18.26	84.12
9	Midecamycin HL60 UP	0.05166	0.3009	22.65	67.12
10	IRON CTD 00006166	0.05281	0.3009	22.14	65.12

## Discussion

The skin is the largest organ of the human body, providing the necessary protective barrier for the internal organs of the body, protecting the human body from external aggressions such as microorganisms, chemicals and physical agents, ultraviolet (UV) radiation in sunlight, etc., and contributing to thermoregulation . Maintaining the self-renewal capacity of the skin is essential for the survival of the organism^20^. This unique barrier is formed by the epidermis, dermis, subcutaneous tissue, and many appendages such as hair follicles, sweat glands, and sebaceous glands^21^. The skin of adults is composed of cells from different embryonic origins and differentiates into three layers in the skin: the epidermis, the dermis, and the subcutaneous layer. The epidermis, the outermost layer of the skin, originates from the ectoderm and is closely connected to the dermal ridges. The epidermis includes the basal layer, spinous layer, granular layer, and stratum corneum, and is composed of keratinocytes, Langerhans cells, melanocytes, neuroendocrine cells, and inflammatory cells^22,23^. The main role of the outermost epidermis is to act as a protective barrier between the external world and the internal environment of the body^24^. Epidermal homeostasis depends on the differentiation of epidermal stem cells present in the basal layer, which provide new cells to replace those lost following tissue renewal or injury. Epidermal stem cells located in the basal layer of the epidermis have lifelong self-renewal capacity and function through intermediates called transport-amplifying cells, which can maintain a certain number through division while continuing to differentiate into different levels of skin tissue. Thus, the epidermis is in a state of continuous proliferation, differentiation and apoptosis^25^, ensuring the production of a large number of keratinocytes required to maintain epidermal homeostasis. The microenvironment provided by the complex structure of the extracellular matrix and basement membrane helps embryonic stem cells to form a complete stem cell niche^26^. Different skin stem cells help maintain and repair various epidermal tissues of the skin, including the interfollicular epidermis, hair follicles, and sebaceous glands. Adult embryonic stem cells can self-renew while their progeny differentiate, leaving the basal layer and migrating toward the epidermal surface. Different types of resident stem or progenitor cells present in different layers of the skin maintain the stability of the human body's internal environment^27^.

In contrast, the dermis is composed of a complex extracellular matrix (ECM) and dermal fibroblasts that provide the skin with strength and elasticity. The dermal fibroblast population includes papillary fibroblasts, reticular fibroblasts, and dermal papillary cells^6^. Dermal fibroblasts maintain skin homeostasis through interactions with the epidermis and extracellular matrix. Dermal fibroblasts provide structural support to the skin by producing collagen and elastin and other structures under the epidermis. Due to the production of these basic structural substances, dermal fibroblasts play an important role in maintaining tissue structure and preventing skin aging. The synthesis and degradation of dermal matrix molecules undergo profound changes with age^1,28^. Aging of dermal fibroblasts leads to skin-related functional defects, resulting in reduced synthesis of structural substances such as collagen, elastin, hyaluronic acid, and chondroitin, the density of dermal collagen, as well as the density of various types of skin cells were continuously lower^29^.

The self-renewal of dermal and epidermal cells of the skin is accomplished through gene regulation in time and space. Skin as the main barrier between self and environment, natural aging and photoaging may occur in parallel or overlapping, which may cause adverse effects on skin and cause skin aging, and their leading mechanism is those genes involved in skin aging^30^. There are several specific genes and signaling pathways involved in the regulation of epidermal stem cells and dermal fibroblasts, which are the result of complex interactions of multiple genes and signaling pathways that synergistically regulate the characteristics of dermal fibroblasts and the function of epidermal stem cells. In this study, based on the analysis of GEO database, dermal fibroblasts and epidermal stem cells samples isolated from the skin of mice of different ages in GEO database were selected, and they were divided into young group and old group according to the age of mice. Data sets GSE110978 and GSE117763 included DEGs of dermal fibroblasts in different age groups. The GSE137176 dataset included the identification of epidermal stem cell genes DEGs in mice of different age groups. By comparing 592 dermal fibroblast genes collected by GSE110978 and GSE117763 with 1519 epidermal stem cell genes collected by GSE137176, We identified six common DEGs (Arhgap24, Peg3, Mpzl1, Col14a1, Cybrd1, and Trf). The comprehensive comparison and analysis of these 6 skin aging related genes can find the regulation rules of skin aging related genes. Extensive recombination of hair follicle stem cells occurs in the aging process, which is manifested as weakened self-renewal ability and delayed response to activation signals, due to the combined effect of internal microenvironment and external macro-environmental regulatory factors^31^. Embryonic stem cells fail with age and no longer stabilize the internal environment of epidermal tissue or repair damaged tissue^12,32^.

Arhgap24, this gene encodes a Rho-GTPase activating protein, Arhgap24 is specific for the small GTPase family member Rac. Arhgap24 belongs to Rho GTPase activating protein (RhoGAP), which is a kind of protein containing 748 amino acids. It is involved in cell cycle, apoptosis and invasion^33^. Peg3 may play a role in skin cell proliferation and p53-mediated apoptosis. One study showed that peG3-mutated mice had excess fat in the abdomen, subcutaneous and scapular area, even with low food intake^34^. This suggests that lipid metabolism is influenced by Peg3 mutant model^35^, and Peg3 may be responsible for the regulation of lipid metabolism. As a highly conserved protein, Mpzl1 is expressed in a wide variety of cells, suggesting that it may play an important role in the basic functions of skin cells. Previous studies have shown that Mpzl1 may promote fiber-connexin-dependent cell migration by recruiting and activating SHP2^36^. Recent studies have also suggested that Mpzl1 may promote fibronectin dependent migration of mouse embryonic fibroblasts^37^. Col14a1, this gene encodes the alpha chain of type XIV collagen, a member of the FACIT (fibril-associated collagens with interrupted triple helices) collagen family. Type XIV collagen interacts with the fibril surface and is involved in the regulation of fibrillogenesis^38^. Cybrd1 is an iron reductase that regulates iron, The iron-regulated signaling pathway mediated by catalytic conversion of iron into ferrous ions during iron absorption is believed to play a physiological role in dietary iron absorption^39^. Trf is the main plasma siderophore, which delivers iron to tissues through binding to its receptor (TrfR) and receptor-mediated endocytosis^40^. It is generally accepted that iron transport is the main function of Trf. Expression of highly conserved ontogenetic and phylogenetic patterns suggests that Trf/ TRFR-mediated iron delivery pathways are required for normal development^41^. Iron is known to be essential for cell division, and this is reflected in the aging process of the skin.

Other related genes and signaling pathways have also been implicated in skin aging. In terms of epidermal stem cells, comprehensive analysis of genes related to aging and skin diseases showed that aqp5 regulates the proliferation and differentiation of epidermal stem cells during skin aging, and may play an important role in maintaining the growth potential of keratinocytes and balancing the proliferation and differentiation of skin^42,43^. Wnt1 induces excessive proliferation of hair follicle cells and rapid depletion of stem cells^44^. The expressions of pro-inflammatory genes IRF4 and Cxcl12 are up-regulated in aging skin cells, which may lead to age-related skin diseases with increased susceptibility^45^. Ecm1, an extracellular interstitial molecule, maintains differentiation and maturation of epidermis, keratinocyte adhesion and signal transduction, and plays a role in angiogenesis and dermis through ECM formation^46^. In terms of dermal fibroblasts, factors secreted by dermal fibroblasts affect the growth and differentiation of keratinocytes, and keratinocyte proliferation depends on the expression of PTN and SDF-1 in fibroblasts, indicating cell–cell interaction^47^. A feature of dermal fibroblasts in aging includes a decrease in papillary fibroblasts and an increase in reticular fibroblasts in the skin^48^. YAP signal has been shown to adjust ECM strength by adjusting Hippo pathway to adapt and respond to mechanical stress^49^. In addition, the FAK signaling pathway plays a role in the response of wound repair to mechanical stress^50^. FAK signaling is triggered by skin injury, and monocyte chemoattractor protein-1 (McP-1) expression is induced by ERK signaling. Fibroblasts also produce IGF-1, which binds to igF-1 receptors (IGF-1Rs) on keratinocytes to activate signaling pathways that regulate cell proliferation and cell response to genotoxic stress such as ULTRAVIOLET radiation to avoid skin aging^51^. Signaling pathways involved in regulating skin aging also include the Notch pathway, which plays a role in regulating inflammation and hair follicle morphogenesis^52,53^. TGF-β signaling also plays an important role in wound healing and scar formation. BMP signaling is involved in the regulation of hair follicle morphogenesis^54,55^. In addition, BMP signaling is a necessary differentiation transfer for myofibroblast to adipocyte transformation in wound healing^56^.

We also performed GO and KEGG analysis on the top 10 hub genes analyzed by the Cytohubba plugin, and found that the enriched "BP" mainly included protein heterooligomerization, necrotic cell death, programmed necrotic cell death; in terms of "CC", hub The genes were mainly enriched in the membrane region, the membrane microdomain, and the membrane raft region. The "MF" aspect suggested that the hub gene was mainly related to the processes of tumor necrosis factor receptor superfamily binding, MAP kinase activity, and death domain binding. KEGG showed that hub gene was related to TNF signaling pathway. Apoptosis and NF-kappa B signaling pathway. We can speculate from this that senescence may be linked to the suppression of the expression of epidermal stem cells and dermal fibroblasts.

In the past decade, miRNAs have been tried and found widely involved in the regulation of almost all processes in an organism, including skin aging. In addition, most miRNAs associated with proteins in aging skin were down-regulated. In our study, two genes, Peg3 and Cybrd1, were shown to interact with three miRNAs, among which mmu-miR-1931 interacted with Peg3, Cybrd1 interacted with mmu-miR-290a-5p, mmu-miR-3082-5p there is a link, and it also appears to be down-regulated. At the same time, other studies have further shown that other related miRNAs are also responsible for regulating skin aging-related processes. For example, Mancini et al. found that mir-152 and miR-181a induce senescence in proliferating human skin fibroblasts and play a multifaceted role in dermal extracellular matrix remodeling in aged skin^57^. Further studies have confirmed that the expression of dnmt1 and mir-217 is negatively correlated in skin tissue and fibroblasts of different ages. Showed that mir-217 promotes fibroblast senescence by targeting dnmt13′-UTR to inhibit dnmt1-mediated methylation of p16 and pRb^58^. In addition, mir-1299 inhibits the senescence of fibroblasts by inhibiting the expression of arg2, and the miR-1299/ARG2/arl1 axis is a novel pathway by which arg2 participates in the inhibition of cellular senescence by inhibiting autophagy^59^. Rivetti et al. found that keratinocyte miR-138, miR-181a, miR-181b and miR-130b were up-regulated during replicative senescence. They showed that these four miRNAs regulate cell proliferation pathways by targeting p63 and Sirtuin 1 (SIRT1) mRNAs^60^. In addition, we have also done two analyses of drugs related to gene regulation, Protein-chemical Interactions and Identification of candidate drugs. Including the Trf gene-related chemical drugs in the regulatory genes of epidermal stem cells and dermal fibroblasts provided by NetworkAnalyst, and the drug molecules identified by the Enrichr platform that are related to 6 common DEGs. These drug candidates also provide inspiration for the research and development of skin care ingredients related to skin aging.

## Conclusion summary

This article analyzes the age-dependent expression of epidermal stem cell genes and dermal fibroblast genes associated with skin aging by means of bioinformatics analysis, which is based on the analysis of epidermal stem cell and dermal fibroblast cell samples from young and old mice. , based on these three datasets identified cell-type-specific DEGs, emphasizing molecular changes during skin aging. Six genes related to skin aging, as well as related gene-mediated signaling pathways and apoptosis signaling pathways, as well as genes related to increased inflammation were up-regulated with age, and genes related to epithelial cell proliferation and epithelial maintenance were identified by analysis. Expression is down-regulated with age. Specifically, skin aging reduces the self-renewal capacity of epidermal stem cells and dermal fibroblasts and promotes aging phenotypes, including increased inflammatory responses, which may be key factors driving epithelial wear and skin atrophy and skin aging. Comprehensive analysis of the expression characteristics and significance of these six common DEGs (Arhgap24, Peg3, Mpzl1, Col14a1, Cybrd1, Trf) in skin aging can provide theoretical support and guidance for further research on skin aging-related functions (Supplementary Information).

## Supplementary Information

Supplementary Information.

## Data Availability

The datasets used and/or analyzed during the current study are available from the corresponding author on reasonable request.

## References

[1] Lavker RM, Zheng PS, Dong G (1987). Aged skin: a study by light, transmission electron, and scanning electron microscopy. J. Invest. Dermatol..

[2] Daly CH, Odland GF (1979). Age-related changes in the mechanical properties of human skin. J. Invest. Dermatol..

[3] Baumann L (2007). Skin ageing and its treatment. J. Pathol..

[4] Campisi J (2013). Aging, cellular senescence, and cancer. Annu. Rev. Physiol..

[5] Schneider EL, Mitsui Y (1976). The relationship between in vitro cellular aging and in vivo human age. Proc. Natl. Acad. Sci. U. S. A..

[6] Lynch MD, Watt FM (2018). Fibroblast heterogeneity: implications for human disease. J. Clin. Invest..

[7] Gilchrest BA (1996). A review of skin ageing and its medical therapy. Br. J. Dermatol..

[8] El-Domyati M, Attia S, Saleh F, Brown D, Birk DE, Gasparro F, Ahmad H, Uitto J (2002). Intrinsic aging vs photoaging: a comparative histopathological, immunohistochemical, and ultrastructural study of skin. Exp. Dermatol..

[9] Petek LM, Fleckman P, Miller DG (2010). Efficient KRT14 targeting and functional characterization of transplanted human keratinocytes for the treatment of epidermolysis bullosa simplex. Mol. Ther..

[10] Blanpain C, Fuchs E (2006). Epidermal stem cells of the skin. Annu. Rev. Cell Dev. Biol..

[11] Taub AF, Pham K (2018). Stem cells in dermatology and anti-aging care of the skin. Facial Plast. Surg. Clin. North Am..

[12] Gerasymchuk M, Cherkasova V, Kovalchuk O, Kovalchuk I (2020). The role of microRNAs in organismal and skin aging. Int. J. Mol. Sci..

[13] Edgar R, Domrachev M, Lash AE (2002). Gene expression omnibus: NCBI gene expression and hybridization array data repository. Nucl. Acids Res..

[14] Smyth GK, Gentleman R, Care V, Dudoit S (2005). Limma: linear models for microarray data. Bioinformatics and computational biology solutions using R and bioconductor.

[15] He X, Yang K, Wang H, Chen X, Wu H, Yao L, Ma S (2018). Expression and clinical significance of survivin in ovarian cancer: a meta-analysis. PLoS ONE.

[16] Szklarczyk D, Gable AL, Lyon D, Junge A, Wyder S, Huerta-Cepas J, Simonovic M, Doncheva NT, Morris JH, Bork P, Jensen LJ, Mering CV (2019). STRING v11: protein-protein association networks with increased coverage, supporting functional discovery in genome-wide experimental datasets. Nucl. Acids Res..

[17] Shannon P, Markiel A, Ozier O, Baliga NS, Wang JT, Ramage D, Amin N, Schwikowski B, Ideker T (2003). Cytoscape: a software environment for integrated models of biomolecular interaction networks. Genome Res..

[18] Chin, C. H. *et al.* cytoHubba: identifying hub objects and sub-networks from complex interactome. *BMC Syst Biol.* **8**(Suppl 4), S11 (2014). 10.1186/1752-0509-8-S4-S11 PMC4290687 25521941

[19] Chen, E. Y. *et al.* Enrichr: interactive and collaborative HTML5 gene list enrichment analysis tool. *BMC Bioinf.* **15**(14), 128 (2013). 10.1186/1471-2105-14-128 PMC3637064 23586463

[20] Zouboulis, C. C. & Makrantonaki, E. Clinical aspects and molecular diagnostics of skin aging. *Clin. Dermatol.* **29**(1), 3–14 (2011). 10.1016/j.clindermatol.2010.07.001 21146726

[21] Venus M, Waterman J, McNab I (2011). Basic physiology of the Skin. Surgery..

[22] Merad M, Ginhoux F, Collin M (2008). Origin, homeostasis and function of Langerhans cells and other langerin-expressing dendritic cells. Nat. Rev. Immunol..

[23] Mort RL, Jackson IJ, Patton EE (2015). The melanocyte lineage in development and disease. Development.

[24] Fuchs E (2009). Finding one's niche in the skin. Cell Stem Cell.

[25] Kammeyer A, Luiten RM (2015). Oxidation events and skin aging. Ageing Res. Rev..

[26] Moore KA, Lemischka IR (2006). Stem cells and their niches. Science.

[27] Blanpain C, Fuchs E (2009). Epidermal homeostasis: a balancing act of stem cells in the skin. Nat. Rev. Mol. Cell Biol..

[28] Carrino DA, Onnerfjord P, Sandy JD, Cs-Szabo G, Scott PG, Sorrell JM, Heinegård D, Caplan AI (2003). Age-related changes in the proteoglycans of human skin: specific cleavage of decorin to yield a major catabolic fragment in adult skin. J. Biol. Chem..

[29] Zou Z, Long X, Zhao Q, Zheng Y, Song M, Ma S, Jing Y, Wang S, He Y, Esteban CR, Yu N, Huang J, Chan P, Chen T, Izpisua Belmonte JC, Zhang W, Qu J, Liu GH (2021). A single-cell transcriptomic atlas of human skin aging. Dev. Cell..

[30] DiLoreto R, Murphy CT (2015). The cell biology of aging. Mol. Biol. Cell..

[31] Zouboulis CC, Adjaye J, Akamatsu H, Moe-Behrens G, Niemann C (2008). Human skin stem cells and the ageing process. Exp Gerontol..

[32] López-Otín C, Blasco MA, Partridge L, Serrano M, Kroemer G (2013). The hallmarks of aging. Cell.

[33] Zhang S, Sui L, Zhuang J, He S, Song Y, Ye Y, Xia W (2018). ARHGAP24 regulates cell ability and apoptosis of colorectal cancer cells via the regulation of P53. Oncol. Lett..

[34] Curley JP, Pinnock SB, Dickson SL, Thresher R, Miyoshi N, Surani MA, Keverne EB (2005). Increased body fat in mice with a targeted mutation of the paternally expressed imprinted gene Peg3. FASEB J..

[35] Kim J, Frey WD, He H, Kim H, Ekram MB, Bakshi A, Faisal M, Perera BP, Ye A, Teruyama R (2013). Peg3 mutational effects on reproduction and placenta-specific gene families. PLoS ONE.

[36] Taniguchi K, Karin M (2014). IL-6 and related cytokines as the critical lynchpins between inflammation and cancer. Semin. Immunol..

[37] Roubelakis MG, Martin-Rendon E, Tsaknakis G, Stavropoulos A, Watt SM (2007). The murine ortholog of the SHP-2 binding molecule, PZR accelerates cell migration on fibronectin and is expressed in early embryo formation. J. Cell Biochem..

[38] Tono-Oka S, Tanase S, Miike T, Tanaka H (1996). Transient expression of collagen type XIV during muscle development and its reappearance after denervation and degeneration. J. Histochem. Cytochem..

[39] Sandberg AS, Önning G, Engström N, Scheers N (2018). Iron supplements containing lactobacillus plantarum 299v increase ferric iron and up-regulate the ferric reductase DCYTB in human Caco-2/HT29 MTX Co-cultures. Nutrients.

[40] Kaplan J (2002). Mechanisms of cellular iron acquisition: another iron in the fire. Cell.

[41] Levy JE, Jin O, Fujiwara Y, Kuo F, Andrews NC (1999). Transferrin receptor is necessary for development of erythrocytes and the nervous system. Nat. Genet..

[42] Zhou J, Dong Y, Liu J, Ren J, Wu J, Zhu N (2020). AQP5 regulates the proliferation and differentiation of epidermal stem cells in skin aging. Braz. J. Med. Biol. Res..

[43] Giangreco A, Qin M, Pintar JE, Watt FM (2008). Epidermal stem cells are retained in vivo throughout skin aging. Aging Cell.

[44] Castilho RM, Squarize CH, Chodosh LA, Williams BO, Gutkind JS (2009). mTOR mediates Wnt-induced epidermal stem cell exhaustion and aging. Cell Stem Cell.

[45] Piñero, J. *et al.* DisGeNET: a comprehensive platform integrating information on human disease-associated genes and variants. *Nucl. Acids Res.* **45**(D1), D833–D839 (2017). 10.1093/nar/gkw943 PMC5210640 27924018

[46] Gao F, Xia Y, Wang J, Lin Z, Ou Y, Liu X, Liu W, Zhou B, Luo H, Zhou B, Wen B, Zhang X, Huang J (2014). Integrated analyses of DNA methylation and hydroxymethylation reveal tumor suppressive roles of ECM1, ATF5, and EOMES in human hepatocellular carcinoma. Genome Biol..

[47] Florin L, Maas-Szabowski N, Werner S, Szabowski A, Angel P (2005). Increased keratinocyte proliferation by JUN-dependent expression of PTN and SDF-1 in fibroblasts. J. Cell Sci..

[48] Lämmermann I, Terlecki-Zaniewicz L, Weinmüllner R, Schosserer M, Dellago H, de Matos Branco AD, Autheried D, Sevcnikar B, Kleissl L, Berlin I, Morizot F, Lejeune F, Fuzzati N, Forestier S, Toribio A, Tromeur A, Weinberg L, Higareda Almaraz JC, Scheideler M, Rietveld M, El Ghalbzouri A, Tschachler E, Gruber F, Grillari J (2018). Blocking negative effects of senescence in human skin fibroblasts with a plant extract. NPJ Aging Mech. Dis..

[49] Calvo F, Ege N, Grande-Garcia A, Hooper S, Jenkins RP, Chaudhry SI, Harrington K, Williamson P, Moeendarbary E, Charras G, Sahai E (2013). Mechanotransduction and YAP-dependent matrix remodelling is required for the generation and maintenance of cancer-associated fibroblasts. Nat. Cell Biol..

[50] Wong VW, Rustad KC, Akaishi S, Sorkin M, Glotzbach JP, Januszyk M, Nelson ER, Levi K, Paterno J, Vial IN, Kuang AA, Longaker MT, Gurtner GC (2011). Focal adhesion kinase links mechanical force to skin fibrosis via inflammatory signaling. NatMed..

[51] Mahajan AS, Arikatla VS, Thyagarajan A, Zhelay T, Sahu RP, Kemp MG, Spandau DF, Travers JB (2021). Creatine and nicotinamide prevent oxidant-induced senescence in human fibroblasts. Nutrients.

[52] Hu B, Lefort K, Qiu W, Nguyen BC, Rajaram RD, Castillo E, He F, Chen Y, Angel P, Brisken C, Dotto GP (2010). Control of hair follicle cell fate by underlying mesenchyme through a CSL-Wnt5a-FoxN1 regulatory axis. Genes Dev..

[53] Hu B, Castillo E, Harewood L, Ostano P, Reymond A, Dummer R, Raffoul W, Hoetzenecker W, Hofbauer GF, Dotto GP (2012). Multifocal epithelial tumors and field cancerization from loss of mesenchymal CSL signaling. Cell.

[54] Clavel C, Grisanti L, Zemla R, Rezza A, Barros R, Sennett R, Mazloom AR, Chung CY, Cai X, Cai CL, Pevny L, Nicolis S, Ma'ayan A, Rendl M (2012). Sox2 in the dermal papilla niche controls hair growth by fine-tuning BMP signaling in differentiating hair shaft progenitors. Dev. Cell..

[55] Plikus MV, Baker RE, Chen CC, Fare C, de la Cruz D, Andl T, Maini PK, Millar SE, Widelitz R, Chuong CM (2011). Self-organizing and stochastic behaviors during the regeneration of hair stem cells. Science.

[56] Sharov, A. A. *et al.* DisGeNET: a comprehensive platform integrating information on human disease-associated genes and variants. *Proc. Natl. Acad. Sci. U. S. A.* **103**(48), 18166–18171 (2006).

[57] Mancini M, Lena AM, Saintigny G, Mahé C, Di Daniele N, Melino G, Candi E (2014). MicroRNAs in human skin ageing. Ageing Res Rev..

[58] Wang B, Du R, Xiao X, Deng ZL, Jian D, Xie HF, Li J (2017). Microrna-217 modulates human skin fibroblast senescence by directly targeting DNA methyltransferase 1. Oncotarget.

[59] Kim HJ, Kim B, Byun HJ, Yu L, Nguyen TM, Nguyen TH, Do PA, Kim EJ, Cheong KA, Kim KS, Huy Phùng H, Rahman M, Jang JY, Rho SB, Kang GJ, Park MK, Lee H, Lee K, Cho J, Han HK, Kim SG, Lee AY, Lee CH (2021). Resolvin D1 Suppresses H2O2-Induced Senescence in Fibroblasts by Inducing Autophagy through the miR-1299/ARG2/ARL1 Axis. Antioxidants (Basel)..

[60] Glass, D., Viñuela, A., Davies, M.N., Ramasamy, A., Parts, L., Knowles, D., Brown, A.A., Hedman, A.K., Small, K.S., Buil, A., Grundberg, E., Nica, A.C., Di Meglio, P., Nestle, F.O., Ryten, M., UK Brain Expression consortium, MuTHER consortium, Durbin, R., McCarthy, M.I., Deloukas, P., Dermitzakis, E.T., Weale, M.E., Bataille, V., Spector, T.D. Gene expression changes with age in skin, adipose tissue, blood and brain. Genome Biol. **14**(7), R75 (2013). 10.1186/gb-2013-14-7-r75 PMC4054017 23889843

