# Supplementary material for: Differential gene screening and bioinformatics analysis of epidermal stem cells and dermal fibroblasts during skin aging
Source: Sci Rep. 2022 Jul 14;12:12019. doi: 10.1038/s41598-022-16314-z (PMC9283434; doi:10.1038/s41598-022-16314-z)

**Supplementary Materials:**

**Table S1 GSE137176 Full length DEGs**

| **ID** | **adj.P.Val** | **P.Value** | **t** | **B** | **logFC** | **Gene.symbol** |
| --- | --- | --- | --- | --- | --- | --- |
| 10366951 | 0.0041 | 7.12E-07 | -15.9897295 | 6.446359 | -1.94213541 | Ndufa4l2 |
| 10497265 | 0.0041 | 8.92E-07 | 15.481616 | 6.268024 | 2.83408257 | Fabp4 |
| 10351131 | 0.0041 | 1.62E-06 | 14.218029 | 5.783655 | 3.85014142 | Myoc |
| 10542965 | 0.0041 | 1.83E-06 | 13.9655092 | 5.67925 | 2.13131405 | Sgce |
| 10358587 | 0.0041 | 2.30E-06 | 13.5098911 | 5.483907 | 1.88893451 | Hmcn1 |
| 10496359 | 0.0041 | 2.90E-06 | 13.0702052 | 5.286456 | 2.70110261 | Emcn |
| 10358517 | 0.0041 | 2.95E-06 | 13.0329682 | 5.269314 | 1.74559695 | Hmcn1 |
| 10358597 | 0.0041 | 3.12E-06 | 12.927789 | 5.22053 | 1.98321833 | Hmcn1 |
| 10471486 | 0.0041 | 3.15E-06 | 12.9155705 | 5.214828 | 2.02817127 | Eng |
| 10559796 | 0.0041 | 3.23E-06 | 12.8632123 | 5.19031 | 1.67271346 | Peg3 |
| 10358670 | 0.0041 | 3.40E-06 | 12.7709213 | 5.146762 | 1.93854268 | Hmcn1 |
| 10376142 | 0.0041 | 3.43E-06 | 12.7558875 | 5.139628 | 2.70820765 | Oser1 |
| 10358555 | 0.0041 | 3.52E-06 | 12.7070352 | 5.116367 | 1.7345041 | Hmcn1 |
| 10361682 | 0.0041 | 3.68E-06 | 12.6265784 | 5.077795 | 1.80836967 | Ppil4 |
| 10497587 | 0.0041 | 3.82E-06 | 12.5584198 | 5.044859 | 1.84118663 | Yae1d1 |
| 10604932 | 0.0041 | 3.82E-06 | 12.5555057 | 5.043446 | 1.64595533 | Cd99l2///Cd99l2 |
| 10358633 | 0.0041 | 4.13E-06 | 12.4157513 | 4.975141 | 2.16146008 | Hmcn1 |
| 10598087 | 0.0041 | 4.17E-06 | -12.3997111 | 4.967236 | -3.80427045 | ND6///Acsm2 |
| 10430344 | 0.0041 | 4.20E-06 | -12.3840446 | 4.959502 | -1.76395783 | Il2rb |
| 10473312 | 0.0041 | 4.24E-06 | 12.3690096 | 4.952068 | 2.28943697 | Fam171b |
| 10358571 | 0.0041 | 4.48E-06 | 12.2683966 | 4.902008 | 1.84819652 | Hmcn1 |
| 10404402 | 0.0041 | 4.50E-06 | -12.261661 | 4.898637 | -1.8931551 | Foxq1 |
| 10585803 | 0.0041 | 4.83E-06 | -12.1353953 | 4.834998 | -2.07111544 | Stra6 |
| 10358601 | 0.00421 | 5.34E-06 | 11.9613466 | 4.745843 | 1.57962265 | Hmcn1 |
| 10358658 | 0.00421 | 5.57E-06 | 11.8861433 | 4.706798 | 1.98674889 | Hmcn1 |
| 10435641 | 0.00427 | 6.11E-06 | 11.7271806 | 4.623208 | 1.91006356 | Fstl1 |
| 10489053 | 0.00427 | 6.22E-06 | 11.6991403 | 4.608313 | 1.77171408 | 4930518I15Rik |
| 10406419 | 0.00427 | 6.35E-06 | 11.6639358 | 4.589546 | 1.68352486 | Lysmd3 |
| 10514049 | 0.00427 | 6.37E-06 | 11.6568085 | 4.585738 | 1.66984299 | Nfib |
| 10349953 | 0.00427 | 6.70E-06 | -11.5721938 | 4.5403 | -1.69580622 | Chit1 |
| 10358549 | 0.00427 | 6.88E-06 | 11.5272328 | 4.515985 | 1.82759739 | Hmcn1 |
| 10574141 | 0.00427 | 6.88E-06 | -11.5263901 | 4.515528 | -1.69930225 | Nlrc5 |
| 10366476 | 0.00427 | 6.89E-06 | 11.525163 | 4.514862 | 2.41059842 | Ptprb |
| 10358629 | 0.00427 | 6.90E-06 | 11.5220217 | 4.513159 | 1.80161422 | Hmcn1 |
| 10408083 | 0.00427 | 7.60E-06 | -11.3609818 | 4.425021 | -1.79950497 | Hist1h3a///Hist2h3b///Hist1h3i///Hist1h3h///Hist1h3e///Hist1h3b///Hist1h3d///Hist1h3c///Hist1h3f///Hist1h3g///Hist2h3c2///Hist2h3c1///Hist1h3d |
| 10444459 | 0.00427 | 7.78E-06 | -11.3207978 | 4.402783 | -1.67057386 | Tnxb |
| 10408739 | 0.00427 | 7.89E-06 | -11.2974613 | 4.389823 | -1.56033292 | Gm10129 |
| 10358668 | 0.00427 | 8.04E-06 | 11.2670668 | 4.372893 | 1.87999931 | Hmcn1 |
| 10357084 | 0.00427 | 8.09E-06 | 11.2571782 | 4.367373 | 1.53978085 | Serpinb3b |
| 10358631 | 0.00427 | 8.10E-06 | 11.2545841 | 4.365923 | 1.65060419 | Hmcn1 |
| 10503520 | 0.00427 | 8.13E-06 | 11.2491903 | 4.362909 | 1.88010771 | Ttpa |
| 10428707 | 0.00427 | 8.76E-06 | 11.1259268 | 4.293525 | 1.52790317 | Has2 |
| 10358537 | 0.00427 | 8.98E-06 | 11.0856514 | 4.270647 | 1.47979928 | Hmcn1 |
| 10395553 | 0.00427 | 8.98E-06 | 11.0856132 | 4.270625 | 1.54805256 | Nrcam |
| 10554063 | 0.00427 | 9.07E-06 | 11.0710983 | 4.262355 | 1.52396135 | Adamts17 |
| 10391488 | 0.00427 | 9.13E-06 | -11.0600177 | 4.256033 | -1.5002685 | Rdm1///Rnu2-10 |
| 10445268 | 0.00427 | 9.15E-06 | 11.056453 | 4.253997 | 2.1174618 | Adgrf5 |
| 10518167 | 0.00427 | 9.33E-06 | 11.0240801 | 4.235473 | 3.01635287 | Trappc2 |
| 10554808 | 0.00442 | 9.90E-06 | 10.9297318 | 4.181104 | 1.45568435 | Fzd4 |
| 10452793 | 0.00445 | 1.02E-05 | 10.8789302 | 4.15159 | 1.83155096 | Galnt14 |
| 10565204 | 0.00445 | 1.04E-05 | 10.8498815 | 4.134638 | 1.57448279 | Bnc1 |
| 10458046 | 0.00445 | 1.05E-05 | 10.8346155 | 4.125707 | 1.50275542 | Nrep |
| 10436169 | 0.00445 | 1.09E-05 | 10.7799733 | 4.093614 | 1.83159816 | Ift57 |
| 10358619 | 0.00445 | 1.11E-05 | 10.7516798 | 4.076918 | 1.88130262 | Hmcn1 |
| 10475890 | 0.00445 | 1.14E-05 | 10.699158 | 4.045785 | 1.33528024 | Mertk |
| 10358567 | 0.00445 | 1.16E-05 | 10.6841979 | 4.036883 | 1.53513825 | Hmcn1 |
| 10358595 | 0.00445 | 1.19E-05 | 10.6409862 | 4.011086 | 1.69778685 | Hmcn1 |
| 10450372 | 0.00445 | 1.19E-05 | -10.6358055 | 4.007984 | -1.39738429 | Gm10501 |
| 10408239 | 0.00445 | 1.22E-05 | -10.5990278 | 3.985916 | -1.86095387 | Hist1h3a///Hist2h3b///Hist1h3i///Hist1h3h///Hist1h3e///Hist1h3b///Hist1h3d///Hist1h3c///Hist1h3f///Hist1h3g///Hist2h3c2///Hist2h3c1///Hist1h3d |
| 10358625 | 0.00445 | 1.23E-05 | 10.5872672 | 3.978839 | 2.01759738 | Hmcn1 |
| 10358664 | 0.00445 | 1.24E-05 | 10.5761987 | 3.972171 | 1.83519155 | Hmcn1 |
| 10574104 | 0.00445 | 1.27E-05 | -10.5323073 | 3.945644 | -1.71761154 | Nlrc5 |
| 10394054 | 0.00445 | 1.31E-05 | -10.4907108 | 3.920382 | -1.72960414 | Cd7 |
| 10451953 | 0.00445 | 1.32E-05 | 10.4798587 | 3.913772 | 2.39707296 | Lrg1 |
| 10375529 | 0.00445 | 1.32E-05 | 10.4731277 | 3.909668 | 1.50314027 | Zfp62 |
| 10362171 | 0.00445 | 1.33E-05 | 10.4644834 | 3.904392 | 1.38138191 | Stx7 |
| 10358541 | 0.00445 | 1.36E-05 | 10.4330951 | 3.885194 | 1.94553366 | Hmcn1 |
| 10578880 | 0.00445 | 1.36E-05 | 10.4305942 | 3.883661 | 2.12436353 | Tll1 |
| 10565794 | 0.00447 | 1.39E-05 | 10.3975333 | 3.863359 | 1.34497188 | Serpinh1 |
| 10467206 | 0.00447 | 1.44E-05 | 10.3390521 | 3.827259 | 1.70831893 | Ppp1r3c |
| 10458283 | 0.00447 | 1.45E-05 | -10.328247 | 3.820562 | -1.67851829 | Prob1 |
| 10460782 | 0.00448 | 1.47E-05 | -10.3099952 | 3.809232 | -1.52002445 | Gpha2 |
| 10400126 | 0.00448 | 1.49E-05 | 10.2884668 | 3.795837 | 1.80789815 | Lrrn3 |
| 10436602 | 0.00448 | 1.51E-05 | 10.2709553 | 3.784917 | 1.67365021 | Mirlet7c-1 |
| 10427268 | 0.00448 | 1.53E-05 | 10.2513049 | 3.772637 | 1.43645246 | Hoxc13 |
| 10358652 | 0.00448 | 1.56E-05 | 10.2250542 | 3.75619 | 2.15514106 | Hmcn1 |
| 10403980 | 0.00448 | 1.56E-05 | -10.2237336 | 3.755361 | -1.48730495 | Hist1h2br///Hist1h2bq///Hist1h2bp///Hist1h2bn///Hist1h2bm///Hist1h2bl///Hist1h2bk///Hist1h2bj///Hist1h2bh///Hist1h2bg///Hist1h2bf///Hist1h2be///Hist1h2bb///Hist1h2ba///Hist1h2bc |
| 10583297 | 0.00448 | 1.61E-05 | 10.1746937 | 3.724497 | 1.47955063 | Taf1d |
| 10447341 | 0.00448 | 1.62E-05 | 10.1669873 | 3.719631 | 1.59544996 | Rhoq |
| 10358539 | 0.00448 | 1.65E-05 | 10.1390053 | 3.701926 | 2.22675961 | Hmcn1 |
| 10358533 | 0.00448 | 1.65E-05 | 10.1384975 | 3.701604 | 1.4995629 | Hmcn1 |
| 10603166 | 0.00448 | 1.66E-05 | 10.1307294 | 3.696679 | 2.1300926 | Trappc2 |
| 10419216 | 0.00448 | 1.66E-05 | 10.1232768 | 3.691949 | 1.7123645 | Gnpnat1 |
| 10358605 | 0.00448 | 1.68E-05 | 10.1109818 | 3.684137 | 1.5404896 | Hmcn1 |
| 10531931 | 0.00448 | 1.68E-05 | 10.1058289 | 3.68086 | 1.70942605 | Sparcl1 |
| 10420596 | 0.0045 | 1.75E-05 | -10.04981 | 3.645107 | -1.45539338 | Tnfrsf19 |
| 10427461 | 0.0045 | 1.78E-05 | -10.023018 | 3.627926 | -1.28550104 | Ptger4 |
| 10424363 | 0.0045 | 1.81E-05 | 10.0005828 | 3.613498 | 1.88073999 | Nsmce2 |
| 10498058 | 0.0045 | 1.83E-05 | 9.9822618 | 3.601687 | 1.29497789 | Ndufc1 |
| 10593361 | 0.0045 | 1.83E-05 | 9.9811725 | 3.600985 | 1.62952652 | AU019823 |
| 10601778 | 0.0045 | 1.85E-05 | 9.9654546 | 3.590831 | 1.50080763 | Armcx3 |
| 10355500 | 0.0045 | 1.87E-05 | 9.9523997 | 3.582384 | 2.00979369 | Igfbp5 |
| 10376887 | 0.0045 | 1.87E-05 | -9.9522501 | 3.582287 | -1.93927364 | Snord49a |
| 10523506 | 0.0045 | 1.87E-05 | -9.946657 | 3.578664 | -1.55037344 | Bmp3 |
| 10552964 | 0.0045 | 1.89E-05 | -9.9311825 | 3.568628 | -1.30383235 | Gm10252 |
| 10358521 | 0.0045 | 1.90E-05 | 9.9275422 | 3.566264 | 1.26085196 | Hmcn1 |
| 10358662 | 0.0045 | 1.91E-05 | 9.9193005 | 3.560909 | 1.47218511 | Hmcn1 |
| 10358581 | 0.0045 | 1.92E-05 | 9.911557 | 3.555873 | 1.47485681 | Hmcn1 |
| 10425040 | 0.0045 | 1.92E-05 | -9.9099004 | 3.554795 | -1.85765985 | Apol7e///Apol7b |
| 10592106 | 0.0045 | 1.93E-05 | 9.9052148 | 3.551745 | 1.62138874 | Tirap |
| 10358573 | 0.0045 | 1.94E-05 | 9.8951325 | 3.545177 | 1.3670746 | Hmcn1 |
| 10404028 | 0.00452 | 1.96E-05 | -9.8784883 | 3.534317 | -1.35922165 | Hist1h3a///Hist2h3b///Hist1h3i///Hist1h3h///Hist1h3e///Hist1h3b///Hist1h3d///Hist1h3c///Hist1h3f///Hist1h3g///Hist2h3c2///Hist2h3c1///Hist1h3d |
| 10389674 | 0.00453 | 1.98E-05 | 9.8653646 | 3.525739 | 1.18975099 | Dynll2 |
| 10436666 | 0.00455 | 2.02E-05 | 9.8368048 | 3.507026 | 1.82755726 | Jam2 |
| 10358543 | 0.00455 | 2.03E-05 | 9.8331992 | 3.504659 | 1.96849571 | Hmcn1 |
| 10500610 | 0.00456 | 2.08E-05 | 9.7932974 | 3.478399 | 1.75366022 | Fam46c///Fam46c |
| 10399428 | 0.00456 | 2.11E-05 | -9.7737901 | 3.465517 | -2.08830108 | Snord118 |
| 10476106 | 0.00456 | 2.12E-05 | -9.7653728 | 3.459949 | -1.46953426 | Snord57 |
| 10586773 | 0.00456 | 2.14E-05 | 9.7549617 | 3.453055 | 1.53321418 | Gtf2a2 |
| 10404700 | 0.00456 | 2.15E-05 | 9.7462364 | 3.447271 | 1.73271406 | Ubxn2a |
| 10543120 | 0.00456 | 2.16E-05 | 9.7411686 | 3.443909 | 1.43785772 | Ica1 |
| 10566205 | 0.00456 | 2.16E-05 | -9.7369025 | 3.441077 | -1.80919772 | Usp17ld |
| 10465826 | 0.00456 | 2.17E-05 | 9.7326562 | 3.438256 | 1.41851367 | Uqcc3 |
| 10372082 | 0.00456 | 2.18E-05 | 9.728836 | 3.435718 | 1.21820215 | Nudt4 |
| 10416496 | 0.00461 | 2.21E-05 | 9.7040887 | 3.419247 | 1.48906432 | Tpt1 |
| 10541910 | 0.00461 | 2.23E-05 | 9.6922627 | 3.411359 | 2.18106641 | Vwf |
| 10446553 | 0.00461 | 2.24E-05 | 9.6859716 | 3.407158 | 1.45353189 | Epb41l3 |
| 10607004 | 0.00462 | 2.27E-05 | 9.666452 | 3.394105 | 1.94877392 | Psmd10 |
| 10400023 | 0.00465 | 2.31E-05 | 9.6404238 | 3.376653 | 1.74780493 | Tspan13 |
| 10578572 | 0.00467 | 2.37E-05 | 9.607807 | 3.354708 | 1.83559703 | Stox2 |
| 10595211 | 0.00467 | 2.40E-05 | 9.5890671 | 3.342062 | 1.33762809 | Col12a1 |
| 10358666 | 0.00467 | 2.40E-05 | 9.5862203 | 3.340138 | 1.42074318 | Hmcn1 |
| 10358593 | 0.00467 | 2.41E-05 | 9.5823997 | 3.337555 | 1.37519468 | Hmcn1 |
| 10375360 | 0.00467 | 2.42E-05 | 9.5760996 | 3.333294 | 1.28799495 | Ebf1 |
| 10502240 | 0.00467 | 2.43E-05 | 9.5716674 | 3.330295 | 2.11911095 | Npnt |
| 10358432 | 0.00467 | 2.44E-05 | 9.5639458 | 3.325065 | 1.65996275 | Zfp825 |
| 10358621 | 0.00467 | 2.45E-05 | 9.5580338 | 3.321058 | 1.46578671 | Hmcn1 |
| 10358557 | 0.00467 | 2.46E-05 | 9.5550585 | 3.31904 | 2.16097169 | Hmcn1 |
| 10513737 | 0.00471 | 2.49E-05 | 9.5350409 | 3.305446 | 2.61769624 | Gm6133///Rpl17///Hk1 |
| 10574137 | 0.00471 | 2.50E-05 | -9.5272757 | 3.300164 | -1.44576289 | Nlrc5 |
| 10416541 | 0.00499 | 2.69E-05 | 9.4282268 | 3.232366 | 1.24303391 | Enox1 |
| 10358599 | 0.00506 | 2.74E-05 | 9.4018044 | 3.214146 | 1.32486289 | Hmcn1 |
| 10389581 | 0.00506 | 2.75E-05 | 9.3933716 | 3.208319 | 1.46785418 | Ypel2 |
| 10347497 | 0.00506 | 2.77E-05 | -9.3861003 | 3.203289 | -1.17394965 | Wnt10a |
| 10403680 | 0.00511 | 2.83E-05 | 9.354048 | 3.181069 | 1.61728869 | Arid4b |
| 10358615 | 0.00511 | 2.84E-05 | 9.3520218 | 3.179662 | 1.87375769 | Hmcn1 |
| 10408200 | 0.00511 | 2.85E-05 | -9.3473275 | 3.176399 | -1.27948721 | Hist1h4f |
| 10442224 | 0.00519 | 2.92E-05 | 9.3092597 | 3.149878 | 1.94622818 | Zfp948 |
| 10358535 | 0.00519 | 2.93E-05 | 9.3066805 | 3.148077 | 1.24628538 | Hmcn1 |
| 10574415 | 0.00519 | 2.96E-05 | -9.2946751 | 3.139685 | -1.71872507 | 1700047G07Rik |
| 10574143 | 0.00519 | 2.96E-05 | -9.2933304 | 3.138744 | -1.9254449 | Nlrc5 |
| 10369604 | 0.00519 | 2.97E-05 | 9.2882033 | 3.135157 | 1.29322652 | Vps26a |
| 10404132 | 0.00519 | 3.04E-05 | 9.2577286 | 3.113785 | 2.02621 | Cmah |
| 10518837 | 0.00519 | 3.04E-05 | 9.2571774 | 3.113398 | 1.49626447 | Camta1 |
| 10483667 | 0.00519 | 3.08E-05 | 9.2358022 | 3.098361 | 1.6056533 | Cir1 |
| 10358519 | 0.00519 | 3.10E-05 | 9.2287 | 3.093356 | 1.36838845 | Hmcn1 |
| 10512739 | 0.00519 | 3.11E-05 | 9.2260886 | 3.091515 | 1.16497868 | Xpa |
| 10358660 | 0.00519 | 3.12E-05 | 9.2204117 | 3.08751 | 1.39985442 | Hmcn1 |
| 10358565 | 0.00519 | 3.14E-05 | 9.2129487 | 3.082241 | 1.84491727 | Hmcn1 |
| 10516906 | 0.00519 | 3.15E-05 | -9.2043443 | 3.076161 | -1.2328536 | Snora73b |
| 10372988 | 0.00519 | 3.19E-05 | 9.1888628 | 3.065205 | 2.36774127 | Slc16a7 |
| 10358654 | 0.00519 | 3.21E-05 | 9.1799126 | 3.058862 | 1.35649061 | Hmcn1 |
| 10406877 | 0.00519 | 3.27E-05 | 9.1530118 | 3.039756 | 1.96186077 | Serf1 |
| 10358513 | 0.00519 | 3.28E-05 | 9.1513398 | 3.038567 | 1.67033367 | Hmcn1 |
| 10578688 | 0.00519 | 3.29E-05 | -9.1467736 | 3.035317 | -1.69882188 | Rdm1///Rnu2-10 |
| 10497421 | 0.00519 | 3.30E-05 | 9.1439649 | 3.033317 | 1.38789601 | Hps3 |
| 10424140 | 0.00523 | 3.36E-05 | 9.119656 | 3.015981 | 1.99963951 | Col14a1 |
| 10391746 | 0.00523 | 3.38E-05 | 9.1097345 | 3.008891 | 2.1765395 | Gpatch8 |
| 10372503 | 0.00523 | 3.38E-05 | 9.1088224 | 3.008239 | 1.34046307 | Lgr5 |
| 10505187 | 0.00523 | 3.40E-05 | 9.1014444 | 3.00296 | 1.24038219 | Ugcg |
| 10475932 | 0.00523 | 3.44E-05 | -9.0855097 | 2.991544 | -1.18286802 | Fbln7 |
| 10503828 | 0.00523 | 3.47E-05 | 9.0723058 | 2.982068 | 1.16504637 | Lyrm2 |
| 10523727 | 0.00523 | 3.48E-05 | 9.0680551 | 2.979014 | 1.18107725 | Pkd2 |
| 10441064 | 0.00523 | 3.49E-05 | 9.0653433 | 2.977065 | 1.86146171 | Dscr3 |
| 10531776 | 0.00523 | 3.51E-05 | 9.0588684 | 2.972409 | 1.14893526 | Fam175a |
| 10496854 | 0.00523 | 3.55E-05 | 9.0414152 | 2.959839 | 1.5507094 | Ttll7 |
| 10587880 | 0.00523 | 3.57E-05 | 9.0361617 | 2.956051 | 1.47783742 | Pcolce2 |
| 10358563 | 0.00523 | 3.58E-05 | 9.0314176 | 2.952628 | 1.18347579 | Hmcn1 |
| 10607848 | 0.00523 | 3.60E-05 | 9.0255076 | 2.94836 | 1.73370553 | Egfl6 |
| 10539617 | 0.00535 | 3.72E-05 | 8.979065 | 2.914722 | 1.23223913 | Alms1 |
| 10608107 | 0.00537 | 3.80E-05 | 8.9509104 | 2.894238 | 1.65163155 | Uty |
| 10450038 | 0.00537 | 3.81E-05 | 8.9481821 | 2.892249 | 1.10326648 | Angptl4 |
| 10402422 | 0.00537 | 3.83E-05 | 8.9418213 | 2.88761 | 1.26889675 | Serpina9 |
| 10607952 | 0.00537 | 3.83E-05 | 8.9405183 | 2.88666 | 2.42441387 | Vamp7 |
| 10582295 | 0.00537 | 3.87E-05 | 8.9253929 | 2.875613 | 1.12890649 | Odc1 |
| 10449926 | 0.00537 | 3.89E-05 | 8.9190125 | 2.870947 | 1.20318985 | Zfp799 |
| 10582275 | 0.00537 | 3.89E-05 | -8.9182335 | 2.870377 | -1.24515075 | Slc7a5 |
| 10354418 | 0.00537 | 3.98E-05 | 8.8904384 | 2.850006 | 1.20063406 | Nabp1 |
| 10358490 | 0.00537 | 3.98E-05 | 8.888722 | 2.848746 | 1.51613104 | Hmcn1 |
| 10601878 | 0.00537 | 3.98E-05 | 8.888523 | 2.8486 | 2.31122552 | Tceal1 |
| 10396074 | 0.00537 | 3.98E-05 | 8.8878815 | 2.848129 | 1.23008374 | Mgat2 |
| 10603736 | 0.00537 | 3.99E-05 | -8.8868768 | 2.847391 | -1.3579039 | Rdm1///Rnu2-10 |
| 10435271 | 0.00539 | 4.02E-05 | 8.876069 | 2.839449 | 1.32562765 | Heg1 |
| 10429520 | 0.00541 | 4.05E-05 | -8.8669958 | 2.832773 | -1.38003502 | Ly6d |
| 10493858 | 0.00542 | 4.07E-05 | -8.8583607 | 2.826412 | -1.63610351 | Sprr2a3///Sprr2a1 |
| 10540141 | 0.00556 | 4.24E-05 | 8.8059123 | 2.787638 | 1.34488082 | Ccdc174 |
| 10404045 | 0.00556 | 4.26E-05 | -8.7973122 | 2.781257 | -1.29234527 | Hist1h2al///Hist1h2ao///Hist2h2ab///Hist2h2aa2///Hist1h2ai///Hist2h2ac///Hist1h2af///Hist1h2ab///Hist1h2ap///Hist1h2an///Hist1h2ak///Hist1h2ah///Hist1h2ag///Hist1h2ae///Hist1h2ad///Hist1h2ac///Hist1h2aa///Hist3h2a///H2afj///H2afx///Hist2h2aa1///Hist1h3d |
| 10381738 | 0.00556 | 4.27E-05 | -8.7941861 | 2.778935 | -1.42886775 | Wnt3 |
| 10508663 | 0.00556 | 4.28E-05 | -8.7907678 | 2.776396 | -1.60624888 | Laptm5 |
| 10428534 | 0.0056 | 4.35E-05 | 8.7711494 | 2.761803 | 1.74525827 | Trps1 |
| 10596255 | 0.00564 | 4.42E-05 | 8.7490431 | 2.745317 | 1.15311248 | Dnajc13 |
| 10371356 | 0.00564 | 4.43E-05 | 8.7460628 | 2.743091 | 1.23259385 | Appl2 |
| 10408111 | 0.00566 | 4.48E-05 | -8.7324557 | 2.732918 | -1.18443605 | Hist1h2al///Hist1h2ao///Hist2h2ab///Hist2h2aa2///Hist1h2ai///Hist2h2ac///Hist1h2af///Hist1h2ab///Hist1h2ap///Hist1h2an///Hist1h2ak///Hist1h2ah///Hist1h2ag///Hist1h2ae///Hist1h2ad///Hist1h2ac///Hist1h2aa///Hist3h2a///H2afj///H2afx///Hist2h2aa1///Hist1h3d |
| 10453705 | 0.00574 | 4.57E-05 | 8.7048198 | 2.712205 | 1.6464417 | Rab18 |
| 10396079 | 0.00582 | 4.67E-05 | 8.6782119 | 2.692197 | 1.38392294 | Klhdc1 |
| 10505028 | 0.00582 | 4.67E-05 | 8.6762756 | 2.690738 | 2.6206192 | Slc44a1 |
| 10465059 | 0.00582 | 4.69E-05 | -8.6711856 | 2.686902 | -1.09969996 | Ctsw |
| 10413874 | 0.00584 | 4.73E-05 | 8.6597535 | 2.678278 | 1.93772207 | Ogdhl |
| 10354529 | 0.00584 | 4.74E-05 | 8.6589031 | 2.677636 | 1.36836143 | 1700019D03Rik |
| 10457183 | 0.00589 | 4.80E-05 | 8.6416766 | 2.664618 | 1.42258204 | Tmx3 |
| 10518532 | 0.00592 | 4.83E-05 | 8.6318527 | 2.657181 | 1.37743213 | Tardbp |
| 10516908 | 0.00605 | 5.05E-05 | -8.5756434 | 2.61446 | -1.29483657 | Snora73a |
| 10546432 | 0.00607 | 5.11E-05 | 8.5590745 | 2.601811 | 2.60355919 | Adamts9 |
| 10358545 | 0.00607 | 5.13E-05 | 8.5544529 | 2.598279 | 2.21830635 | Hmcn1 |
| 10574149 | 0.00607 | 5.13E-05 | -8.5534531 | 2.597514 | -1.49353054 | Nlrc5 |
| 10499655 | 0.00607 | 5.16E-05 | -8.5473962 | 2.592881 | -1.36681777 | Il6ra |
| 10398453 | 0.00607 | 5.16E-05 | 8.5463624 | 2.59209 | 1.23516323 | Ppp2r5c |
| 10408210 | 0.00607 | 5.18E-05 | -8.5428881 | 2.58943 | -1.05130185 | Hist1h2br///Hist1h2bq///Hist1h2bp///Hist1h2bn///Hist1h2bm///Hist1h2bl///Hist1h2bk///Hist1h2bj///Hist1h2bh///Hist1h2bg///Hist1h2bf///Hist1h2be///Hist1h2bb///Hist1h2bc |
| 10467139 | 0.00607 | 5.20E-05 | 8.5370005 | 2.58492 | 1.10103311 | Lipa |
| 10601303 | 0.00607 | 5.21E-05 | 8.5352715 | 2.583595 | 1.22799147 | Chic1 |
| 10403943 | 0.00611 | 5.26E-05 | -8.5229714 | 2.574161 | -1.34072707 | Hist1h2bm |
| 10600597 | 0.00612 | 5.28E-05 | 8.5172748 | 2.569788 | 1.28545104 | Tmem47 |
| 10606369 | 0.00612 | 5.31E-05 | 8.5092081 | 2.563589 | 1.68406876 | Itm2a |
| 10601044 | 0.00612 | 5.32E-05 | -8.5068773 | 2.561796 | -1.23507701 | Gdpd2 |
| 10362596 | 0.00612 | 5.36E-05 | 8.4982363 | 2.555147 | 1.28860807 | Fyn |
| 10496091 | 0.00612 | 5.37E-05 | 8.4965769 | 2.55387 | 1.15274355 | Lef1 |
| 10454828 | 0.00613 | 5.39E-05 | 8.4896672 | 2.548547 | 1.56054954 | Pnet-ps |
| 10574471 | 0.00617 | 5.47E-05 | 8.4723304 | 2.535172 | 1.41719852 | Cmtm3 |
| 10548905 | 0.00626 | 5.58E-05 | 8.4464933 | 2.515186 | 1.24297601 | Eps8 |
| 10525955 | 0.00628 | 5.62E-05 | -8.437205 | 2.507985 | -1.15587451 | Fzd10 |
| 10408081 | 0.00628 | 5.66E-05 | -8.4276207 | 2.500547 | -1.42355431 | Hist1h1b |
| 10501699 | 0.00628 | 5.69E-05 | 8.4212543 | 2.495601 | 1.1310376 | Agl |
| 10578391 | 0.00628 | 5.73E-05 | 8.4122809 | 2.488624 | 1.41724821 | Smarce1 |
| 10591816 | 0.00628 | 5.77E-05 | 8.4032761 | 2.481614 | 1.10988148 | Dpy19l1 |
| 10442231 | 0.00628 | 5.80E-05 | 8.3977716 | 2.477325 | 1.54291964 | Zfp983 |
| 10428842 | 0.00628 | 5.80E-05 | 8.3960988 | 2.476021 | 1.34245788 | Tatdn1 |
| 10588091 | 0.00628 | 5.87E-05 | 8.3819299 | 2.464966 | 1.15008185 | Cep70 |
| 10414102 | 0.00628 | 5.87E-05 | 8.3810827 | 2.464305 | 1.79293295 | Mmrn2 |
| 10582978 | 0.00631 | 5.94E-05 | 8.3671266 | 2.453396 | 1.37579111 | Msantd4 |
| 10386058 | 0.00631 | 5.98E-05 | 8.3577639 | 2.446067 | 1.4168482 | Sparc |
| 10511843 | 0.00631 | 6.03E-05 | 8.3481017 | 2.438495 | 1.38622925 | Ufl1 |
| 10522303 | 0.00631 | 6.04E-05 | 8.3458126 | 2.436699 | 1.75857747 | Guf1 |
| 10358585 | 0.00631 | 6.04E-05 | 8.3446102 | 2.435756 | 1.09491277 | Hmcn1 |
| 10408689 | 0.00631 | 6.06E-05 | 8.3419192 | 2.433645 | 1.57274671 | Nrn1 |
| 10496715 | 0.00631 | 6.06E-05 | 8.3400733 | 2.432196 | 1.46466114 | Znhit6 |
| 10504194 | 0.00631 | 6.08E-05 | -8.3369169 | 2.429718 | -2.69928308 | LOC100861978///Ccl27b///Gm2506///Gm13306///Ccl27a |
| 10385428 | 0.00635 | 6.14E-05 | -8.3239796 | 2.419551 | -1.21501252 | Itk |
| 10454632 | 0.00636 | 6.16E-05 | 8.3198659 | 2.416315 | 1.79125904 | Camk4 |
| 10457357 | 0.00645 | 6.28E-05 | 8.2967649 | 2.398111 | 1.17470957 | Mpp7 |
| 10348299 | 0.00645 | 6.32E-05 | 8.287502 | 2.390797 | 1.64243207 | 5830472F04Rik |
| 10352969 | 0.00646 | 6.38E-05 | -8.276983 | 2.382482 | -1.10206479 | Npbwr1 |
| 10502881 | 0.00646 | 6.40E-05 | 8.2728327 | 2.379198 | 1.5070588 | St6galnac5 |
| 10358577 | 0.00647 | 6.46E-05 | 8.2609947 | 2.369822 | 1.07760958 | Hmcn1 |
| 10403821 | 0.00647 | 6.47E-05 | -8.257836 | 2.367318 | -1.73517563 | Tcrg-V6///Tcrg-V4///Trgv2 |
| 10529875 | 0.00647 | 6.51E-05 | 8.2515585 | 2.362338 | 1.61166264 | Ldb2 |
| 10565813 | 0.00647 | 6.59E-05 | -8.235955 | 2.349944 | -2.35047898 | Snord15a |
| 10543544 | 0.00647 | 6.60E-05 | 8.2329728 | 2.347573 | 1.08371361 | Lrrc4 |
| 10392947 | 0.00647 | 6.60E-05 | 8.232732 | 2.347381 | 1.82251496 | Sumo2 |
| 10344939 | 0.00647 | 6.62E-05 | 8.2304199 | 2.345542 | 1.479621 | Terf1 |
| 10414025 | 0.00647 | 6.65E-05 | 8.223156 | 2.33976 | 1.46656858 | Gdf10///Gdf10 |
| 10530560 | 0.00647 | 6.68E-05 | -8.2187557 | 2.336255 | -1.37001083 | Slain2 |
| 10442240 | 0.00647 | 6.72E-05 | 8.2114434 | 2.330427 | 1.92205559 | Zfp760 |
| 10534570 | 0.00647 | 6.72E-05 | 8.2106156 | 2.329767 | 1.26392035 | Orai2 |
| 10547073 | 0.00647 | 6.73E-05 | -8.2081932 | 2.327834 | -1.39552824 | Snora7a |
| 10475625 | 0.00647 | 6.76E-05 | 8.2033569 | 2.323975 | 1.72001602 | Eid1 |
| 10574139 | 0.00647 | 6.78E-05 | -8.1991897 | 2.320647 | -1.5924492 | Nlrc5 |
| 10513608 | 0.00647 | 6.79E-05 | -8.197536 | 2.319327 | -1.0336512 | Alad |
| 10426425 | 0.00647 | 6.81E-05 | 8.1935848 | 2.316169 | 1.45490767 | Pdzrn4 |
| 10358656 | 0.00647 | 6.81E-05 | 8.1935307 | 2.316126 | 1.44594069 | Hmcn1 |
| 10410332 | 0.00651 | 6.87E-05 | 8.1829618 | 2.307674 | 1.79508869 | Zfp58 |
| 10475378 | 0.00654 | 6.92E-05 | 8.1739276 | 2.30044 | 1.3758999 | Casc4 |
| 10358591 | 0.00655 | 6.98E-05 | 8.1626363 | 2.291387 | 1.27732166 | Hmcn1 |
| 10505911 | 0.00655 | 7.03E-05 | -8.1550466 | 2.285295 | -1.02527711 | Dmrta1 |
| 10461856 | 0.00655 | 7.03E-05 | -8.1538493 | 2.284334 | -1.5206248 | Gna14 |
| 10518526 | 0.00655 | 7.05E-05 | 8.150757 | 2.28185 | 1.31928694 | Angptl7 |
| 10511665 | 0.00656 | 7.07E-05 | -8.1467844 | 2.278657 | -1.18238955 | Necab1 |
| 10533504 | 0.00657 | 7.15E-05 | 8.1339506 | 2.268332 | 1.19133835 | Ift81 |
| 10407307 | 0.00657 | 7.21E-05 | 8.1234875 | 2.259902 | 1.08281999 | Mocs2 |
| 10484351 | 0.00657 | 7.22E-05 | 8.1222191 | 2.25888 | 1.58425856 | Sumo2 |
| 10471844 | 0.00657 | 7.23E-05 | 8.1192974 | 2.256524 | 1.26909266 | Nek6 |
| 10489535 | 0.00657 | 7.25E-05 | 8.1159375 | 2.253813 | 1.75606791 | Wfdc3 |
| 10472605 | 0.00657 | 7.26E-05 | 8.1142101 | 2.252419 | 1.49584873 | Ssb |
| 10358523 | 0.0066 | 7.32E-05 | 8.105079 | 2.245045 | 1.16809987 | Hmcn1 |
| 10358569 | 0.00661 | 7.39E-05 | 8.0921448 | 2.234585 | 1.47140127 | Hmcn1 |
| 10372417 | 0.00661 | 7.45E-05 | 8.0834654 | 2.227557 | 1.20366623 | Atxn7l3b |
| 10571865 | 0.00661 | 7.45E-05 | 8.0826692 | 2.226912 | 2.31297287 | Scrg1 |
| 10391811 | 0.00661 | 7.47E-05 | -8.0788273 | 2.223798 | -1.23938879 | Kif18b |
| 10399636 | 0.00661 | 7.48E-05 | -8.0783128 | 2.223381 | -1.51031347 | Mrto4 |
| 10374442 | 0.00662 | 7.51E-05 | 8.0723187 | 2.21852 | 1.32317075 | C1d |
| 10358583 | 0.00662 | 7.53E-05 | 8.0701822 | 2.216786 | 1.85720742 | Hmcn1 |
| 10358637 | 0.00662 | 7.57E-05 | 8.0637302 | 2.211548 | 1.56728738 | Hmcn1 |
| 10512487 | 0.00662 | 7.57E-05 | -8.0636954 | 2.21152 | -1.33246677 | Rmrp |
| 10358617 | 0.00662 | 7.61E-05 | 8.0557446 | 2.205059 | 1.80415597 | Hmcn1 |
| 10376885 | 0.00662 | 7.62E-05 | -8.0546585 | 2.204176 | -1.80817039 | Snord49b |
| 10600044 | 0.00672 | 7.76E-05 | 8.0330334 | 2.186569 | 1.45228028 | Prrg3 |
| 10570957 | 0.00673 | 7.78E-05 | 8.0292019 | 2.183445 | 1.21940822 | Sfrp1 |
| 10450920 | 0.00677 | 7.85E-05 | -8.0181095 | 2.174391 | -1.97162082 | AY036118 |
| 10385495 | 0.00687 | 8.04E-05 | 7.989072 | 2.150631 | 2.16020971 | Cdk2ap1 |
| 10427796 | 0.00691 | 8.13E-05 | -7.9759998 | 2.139906 | -1.16912669 | Npr3 |
| 10566870 | 0.00693 | 8.18E-05 | 7.9680009 | 2.133335 | 1.07071571 | Tmem41b |
| 10491058 | 0.00693 | 8.19E-05 | -7.9661924 | 2.131848 | -1.13375012 | Rprl2 |
| 10510215 | 0.00693 | 8.22E-05 | 7.9620344 | 2.128429 | 1.4798127 | Zfp991 |
| 10590427 | 0.00693 | 8.24E-05 | -7.9594246 | 2.126282 | -1.52923432 | Ackr2 |
| 10444883 | 0.00695 | 8.30E-05 | -7.9501131 | 2.118617 | -1.4587804 | Cdsn |
| 10574145 | 0.00695 | 8.33E-05 | -7.9451379 | 2.114517 | -1.98488585 | Nlrc5 |
| 10447591 | 0.00695 | 8.36E-05 | -7.9416014 | 2.111602 | -1.13926998 | Ftl1 |
| 10423030 | 0.00695 | 8.37E-05 | 7.9404848 | 2.110681 | 1.08473617 | Prlr |
| 10489253 | 0.00695 | 8.42E-05 | 7.93194 | 2.10363 | 1.11261476 | Zhx3 |
| 10420935 | 0.00695 | 8.44E-05 | 7.9298411 | 2.101897 | 1.19721765 | Ephx2 |
| 10363163 | 0.00695 | 8.47E-05 | 7.9258828 | 2.098627 | 1.13772301 | Asf1a |
| 10464594 | 0.00695 | 8.51E-05 | -7.9197975 | 2.093598 | -1.07460874 | BC021614 |
| 10599696 | 0.00695 | 8.52E-05 | 7.9176428 | 2.091816 | 1.2902643 | Ddx26b |
| 10523766 | 0.00695 | 8.55E-05 | 7.9143352 | 2.08908 | 1.3114924 | Lrrc8c |
| 10482500 | 0.00695 | 8.57E-05 | 7.9116018 | 2.086818 | 1.56269813 | Rnd3 |
| 10439667 | 0.00695 | 8.57E-05 | 7.9104887 | 2.085896 | 1.69851913 | BC016579 |
| 10585992 | 0.00695 | 8.59E-05 | 7.9076293 | 2.083529 | 1.39239125 | Myo9a |
| 10403948 | 0.00695 | 8.60E-05 | -7.9069678 | 2.082981 | -1.10637312 | Hist1h2br///Hist1h2bq///Hist1h2bp///Hist1h2bn///Hist1h2bm///Hist1h2bl///Hist1h2bk///Hist1h2bj///Hist1h2bh///Hist1h2bg///Hist1h2bf///Hist1h2be///Hist1h2bb///Hist1h2bc |
| 10395910 | 0.00695 | 8.61E-05 | 7.905636 | 2.081878 | 1.34126132 | Pnn |
| 10397081 | 0.00695 | 8.65E-05 | 7.8993921 | 2.076704 | 1.40960428 | Rbm25 |
| 10395103 | 0.00695 | 8.68E-05 | 7.8957976 | 2.073724 | 1.91136116 | Pxdn |
| 10407870 | 0.00695 | 8.71E-05 | 7.8918587 | 2.070456 | 1.16129733 | Mrpl32 |
| 10404783 | 0.00695 | 8.73E-05 | 7.8891911 | 2.068242 | 1.25907177 | Edn1 |
| 10481272 | 0.00695 | 8.78E-05 | 7.8811181 | 2.061538 | 1.29430511 | 1700007K13Rik |
| 10361771 | 0.00695 | 8.82E-05 | 7.8765407 | 2.057734 | 1.26374489 | Plagl1 |
| 10604944 | 0.00697 | 8.86E-05 | 7.8704414 | 2.052662 | 1.4466423 | Gabre |
| 10405866 | 0.00698 | 8.90E-05 | 7.865265 | 2.048354 | 1.06067186 | Gm10139 |
| 10425410 | 0.00706 | 9.02E-05 | -7.8490613 | 2.034851 | -1.16571003 | Grap2 |
| 10351035 | 0.00711 | 9.12E-05 | 7.8354639 | 2.023498 | 1.46526989 | Gas5 |
| 10580522 | 0.00711 | 9.13E-05 | -7.8349952 | 2.023107 | -1.05042305 | Tox3 |
| 10561956 | 0.00711 | 9.16E-05 | -7.8305481 | 2.019389 | -1.35697326 | Nol11///Rps12 |
| 10515696 | 0.00711 | 9.18E-05 | -7.8277454 | 2.017045 | -1.02082081 | Szt2 |
| 10491438 | 0.00711 | 9.27E-05 | 7.8160897 | 2.007289 | 1.2198361 | Ttc14 |
| 10358579 | 0.00711 | 9.28E-05 | 7.8151978 | 2.006542 | 1.24502572 | Hmcn1 |
| 10508917 | 0.00711 | 9.32E-05 | -7.8095055 | 2.001772 | -1.1488416 | Aim1l |
| 10544720 | 0.00715 | 9.45E-05 | 7.7927897 | 1.987744 | 1.54254986 | Hnrnpa2b1 |
| 10487405 | 0.00715 | 9.46E-05 | -7.7919625 | 1.987049 | -1.01626354 | Prom2 |
| 10574438 | 0.00718 | 9.53E-05 | 7.7830798 | 1.979582 | 2.27910881 | Cdh5 |
| 10546450 | 0.00721 | 9.66E-05 | 7.766898 | 1.965958 | 2.11752996 | Adamts9 |
| 10358531 | 0.00721 | 9.66E-05 | 7.7664026 | 1.96554 | 1.01229986 | Hmcn1 |
| 10359754 | 0.00721 | 9.67E-05 | 7.7660823 | 1.96527 | 1.40461915 | Mpzl1 |
| 10345913 | 0.00731 | 9.84E-05 | 7.7449411 | 1.947427 | 1.01971149 | Nck2 |
| 10538356 | 0.00732 | 9.87E-05 | 7.7407626 | 1.943895 | 1.17371642 | Chn2 |
| 10357345 | 0.00735 | 9.95E-05 | 7.7316939 | 1.936222 | 1.18520046 | Nckap5 |
| 10579525 | 0.00735 | 1.00E-04 | 7.7259362 | 1.931346 | 2.00521926 | Plvap |
| 10544837 | 0.00738 | 1.01E-04 | 7.7187988 | 1.925297 | 1.42302728 | Tril |
| 10519504 | 0.00741 | 1.03E-04 | 7.6878256 | 1.898983 | 1.76512146 | Sri |
| 10572932 | 0.00741 | 1.03E-04 | 7.6866124 | 1.897951 | 1.36371831 | Naa20 |
| 10455942 | 0.00741 | 1.03E-04 | 7.6860082 | 1.897436 | 1.11571876 | A730017C20Rik |
| 10428103 | 0.00741 | 1.04E-04 | 7.6843144 | 1.895994 | 1.51572114 | Stk3 |
| 10405783 | 0.00741 | 1.04E-04 | 7.6834883 | 1.89529 | 1.977858 | Mir24-1///2010111I01Rik |
| 10409118 | 0.00741 | 1.05E-04 | -7.6723307 | 1.885781 | -1.25226241 | Wnk2 |
| 10574456 | 0.00741 | 1.05E-04 | 7.6702995 | 1.884048 | 1.04004598 | Cklf |
| 10352867 | 0.00741 | 1.05E-04 | 7.6686065 | 1.882604 | 1.01257709 | Plxna2 |
| 10494405 | 0.00741 | 1.05E-04 | -7.6649396 | 1.879474 | -1.08659084 | Hist1h3a///Hist2h3b///Hist1h3i///Hist1h3h///Hist1h3e///Hist1h3b///Hist1h3d///Hist1h3c///Hist1h3f///Hist1h3g///Hist2h3c2///Hist2h3c1///Hist1h3d |
| 10358589 | 0.00741 | 1.06E-04 | 7.6619645 | 1.876934 | 1.57656383 | Hmcn1 |
| 10503502 | 0.00743 | 1.06E-04 | 7.6526862 | 1.869006 | 1.44447832 | Ttpa |
| 10478048 | 0.00743 | 1.06E-04 | 7.6524167 | 1.868776 | 1.51055488 | Lbp |
| 10478160 | 0.00743 | 1.07E-04 | 7.6497938 | 1.866533 | 1.0547436 | Fam83d |
| 10605319 | 0.00743 | 1.07E-04 | 7.6491035 | 1.865942 | 1.25454398 | Gm44504///Ubl4a |
| 10358559 | 0.00745 | 1.07E-04 | 7.6417155 | 1.85962 | 1.165355 | Hmcn1 |
| 10579769 | 0.00748 | 1.08E-04 | -7.6328423 | 1.852019 | -1.45851849 | Gm12070///Gm5523///3000002C10Rik///Gapdh |
| 10579052 | 0.00748 | 1.08E-04 | 7.6325132 | 1.851737 | 1.32747772 | Gm10033 |
| 10358675 | 0.00751 | 1.09E-04 | -7.622917 | 1.843506 | -1.19705049 | Ivns1abp |
| 10535043 | 0.00751 | 1.09E-04 | 7.621502 | 1.842292 | 1.31201933 | Pdgfa |
| 10592251 | 0.00751 | 1.09E-04 | 7.6186773 | 1.839867 | 1.18203389 | Pknox2 |
| 10596267 | 0.00751 | 1.09E-04 | 7.6182882 | 1.839533 | 1.56677233 | Dnajc13 |
| 10358547 | 0.00758 | 1.11E-04 | 7.6038747 | 1.827145 | 2.14324659 | Hmcn1 |
| 10368495 | 0.00759 | 1.11E-04 | -7.6003885 | 1.824145 | -1.02404248 | Rspo3 |
| 10542714 | 0.0076 | 1.11E-04 | 7.5969433 | 1.821179 | 1.39180125 | Lyrm5 |
| 10506397 | 0.0076 | 1.12E-04 | 7.5943721 | 1.818965 | 1.12460391 | Mier1 |
| 10440467 | 0.00761 | 1.12E-04 | 7.5900196 | 1.815215 | 2.46206335 | Rpl21 |
| 10400006 | 0.00762 | 1.13E-04 | -7.5848916 | 1.810795 | -1.19621368 | Ahr |
| 10458560 | 0.00762 | 1.13E-04 | 7.5844451 | 1.81041 | 1.04031253 | Fgf1 |
| 10508490 | 0.00762 | 1.13E-04 | 7.5827073 | 1.808911 | 1.08036343 | Srsf5 |
| 10594840 | 0.00765 | 1.14E-04 | 7.5702145 | 1.798127 | 1.08757072 | Myzap |
| 10386376 | 0.00766 | 1.14E-04 | -7.5672136 | 1.795534 | -1.22275843 | Wnt3a |
| 10446763 | 0.00774 | 1.16E-04 | 7.5456552 | 1.776877 | 1.31165592 | Lbh |
| 10474700 | 0.00781 | 1.18E-04 | 7.5326546 | 1.765601 | 1.04520262 | Thbs1 |
| 10360580 | 0.00785 | 1.19E-04 | 7.525501 | 1.759389 | 1.24371152 | Tfb2m |
| 10580590 | 0.00797 | 1.21E-04 | -7.5047531 | 1.741339 | -1.10026415 | Gm12070///Gm5523///3000002C10Rik///Gapdh |
| 10546092 | 0.00797 | 1.21E-04 | 7.5010412 | 1.738105 | 1.10127654 | Rab7 |
| 10358553 | 0.00797 | 1.21E-04 | 7.4982571 | 1.735678 | 2.04692144 | Hmcn1 |
| 10407543 | 0.00797 | 1.21E-04 | 7.4968845 | 1.734482 | 1.26374989 | Gtpbp4 |
| 10493850 | 0.00803 | 1.23E-04 | -7.4837389 | 1.72301 | -1.90108307 | Sprr2a2///Sprr2a3///Sprr2b///Sprr2a1 |
| 10358459 | 0.00803 | 1.23E-04 | 7.4816914 | 1.721221 | 1.82268126 | BC003331 |
| 10605884 | 0.00804 | 1.24E-04 | 7.4746215 | 1.715042 | 1.66361868 | Ophn1 |
| 10392221 | 0.00809 | 1.25E-04 | 7.4636816 | 1.705469 | 1.86593442 | Pecam1 |
| 10596275 | 0.00809 | 1.25E-04 | 7.4599434 | 1.702195 | 1.28817045 | Dnajc13 |
| 10361270 | 0.00809 | 1.26E-04 | 7.4500386 | 1.693513 | 1.01321692 | Cd46 |
| 10459905 | 0.00809 | 1.26E-04 | 7.4498408 | 1.693339 | 1.23648643 | Setbp1 |
| 10446777 | 0.00809 | 1.27E-04 | 7.4495492 | 1.693084 | 1.13337217 | Ehd3 |
| 10394366 | 0.00809 | 1.27E-04 | 7.4494185 | 1.692969 | 1.5955811 | Atad2b |
| 10486988 | 0.00809 | 1.27E-04 | 7.4487648 | 1.692396 | 1.50362445 | Duoxa1 |
| 10585990 | 0.00812 | 1.27E-04 | 7.4407451 | 1.685356 | 1.02341422 | Myo9a |
| 10358527 | 0.00812 | 1.28E-04 | 7.4370848 | 1.682141 | 1.16805782 | Hmcn1 |
| 10466304 | 0.00812 | 1.28E-04 | -7.4340144 | 1.679443 | -1.05333456 | Dtx4 |
| 10409265 | 0.00812 | 1.29E-04 | 7.4297066 | 1.675656 | 2.07358872 | Auh |
| 10373073 | 0.00818 | 1.30E-04 | 7.4159443 | 1.663543 | 1.34593026 | Arhgef25 |
| 10437236 | 0.00818 | 1.31E-04 | 7.4136205 | 1.661495 | 1.68067282 | B230307C23Rik |
| 10432511 | 0.00818 | 1.31E-04 | -7.4114051 | 1.659543 | -1.33373614 | Racgap1 |
| 10371271 | 0.00818 | 1.31E-04 | 7.4050889 | 1.653973 | 1.36478921 | Zfp781 |
| 10608442 | 0.00818 | 1.32E-04 | -7.4019608 | 1.651213 | -2.10054633 | Gm20871///Gm20858///Gm20857///Gm20819///Sly///Gm20736///D630029K05Rik///1700040F15Rik |
| 10448235 | 0.0082 | 1.32E-04 | 7.3974424 | 1.647225 | 1.30262894 | Zfp945 |
| 10500345 | 0.00821 | 1.33E-04 | -7.3939278 | 1.64412 | -1.17624807 | Terc |
| 10521471 | 0.00821 | 1.33E-04 | -7.3916056 | 1.642069 | -1.62517896 | Ppp2r2c |
| 10522712 | 0.00823 | 1.34E-04 | 7.3831065 | 1.634554 | 1.24127855 | Rest |
| 10363241 | 0.00828 | 1.35E-04 | 7.3740628 | 1.62655 | 1.18477408 | Gcc2 |
| 10575993 | 0.00829 | 1.35E-04 | -7.3707861 | 1.623647 | -1.4004028 | 6430548M08Rik |
| 10401238 | 0.00829 | 1.36E-04 | 7.3659874 | 1.619394 | 1.07433406 | Zfp36l1 |
| 10407211 | 0.00829 | 1.36E-04 | 7.3638909 | 1.617535 | 1.33930923 | Plpp1 |
| 10392318 | 0.00829 | 1.37E-04 | 7.3620518 | 1.615904 | 1.12850524 | Bptf |
| 10527051 | 0.00829 | 1.37E-04 | 7.3603616 | 1.614405 | 1.44285473 | Sdk1 |
| 10403978 | 0.00833 | 1.38E-04 | -7.3512705 | 1.606335 | -2.94004467 | Hist1h2br///Hist1h2bq///Hist1h2bp///Hist1h2bn///Hist1h2bm///Hist1h2bl///Hist1h2bk///Hist1h2bj///Hist1h2bh///Hist1h2bg///Hist1h2bf///Hist1h2be///Hist1h2bb///Hist1h2ba///Hist1h2bc |
| 10414527 | 0.00835 | 1.38E-04 | 7.3462376 | 1.601863 | 1.15997218 | Pnp2///Pnp |
| 10436196 | 0.00836 | 1.39E-04 | 7.3397331 | 1.59608 | 1.3560679 | Cd47 |
| 10427290 | 0.00838 | 1.40E-04 | 7.3324821 | 1.589627 | 1.93240786 | Hoxc8 |
| 10446351 | 0.00838 | 1.40E-04 | 7.3317659 | 1.588989 | 1.03376051 | Fer |
| 10532944 | 0.00838 | 1.40E-04 | 7.330717 | 1.588055 | 1.4157928 | Mlec |
| 10584231 | 0.00838 | 1.41E-04 | 7.3239617 | 1.582037 | 1.23344629 | Pus3 |
| 10536949 | 0.00838 | 1.42E-04 | 7.3207191 | 1.579146 | 1.17831749 | Strip2 |
| 10383502 | 0.00838 | 1.42E-04 | -7.3197818 | 1.578311 | -1.29352034 | Slc16a3 |
| 10416379 | 0.00838 | 1.42E-04 | 7.3189626 | 1.57758 | 1.37841062 | Sucla2 |
| 10530633 | 0.00838 | 1.42E-04 | 7.3187135 | 1.577358 | 1.27363542 | Sgcb |
| 10396148 | 0.00841 | 1.42E-04 | 7.3136008 | 1.572796 | 1.2755068 | Abhd12b |
| 10581575 | 0.00841 | 1.43E-04 | 7.3093495 | 1.569001 | 1.32726077 | Wdr70///Gm1943 |
| 10426924 | 0.00841 | 1.43E-04 | -7.3062121 | 1.566199 | -1.21079183 | Slc4a8 |
| 10389882 | 0.00841 | 1.44E-04 | 7.3043778 | 1.56456 | 1.59883597 | Luc7l3 |
| 10546434 | 0.00848 | 1.46E-04 | 7.2878115 | 1.549743 | 1.44920174 | Adamts9 |
| 10540273 | 0.00851 | 1.47E-04 | 7.2789681 | 1.54182 | 1.34858214 | Ube2v2 |
| 10390768 | 0.00852 | 1.48E-04 | 7.2727185 | 1.536215 | 1.52260929 | Smarce1 |
| 10425799 | 0.00852 | 1.48E-04 | -7.2720259 | 1.535594 | -1.41759012 | Rnu12 |
| 10399691 | 0.00852 | 1.48E-04 | 7.2699165 | 1.533701 | 1.43855891 | Id2 |
| 10472570 | 0.00853 | 1.49E-04 | 7.265212 | 1.529478 | 1.04558772 | Ppig |
| 10542738 | 0.00853 | 1.49E-04 | 7.2605876 | 1.525324 | 1.09626653 | Rassf8 |
| 10565547 | 0.00853 | 1.50E-04 | 7.2572 | 1.52228 | 1.45973877 | Pcf11 |
| 10408077 | 0.00853 | 1.50E-04 | -7.2556439 | 1.520881 | -1.08094476 | Hist1h2ak |
| 10601771 | 0.00853 | 1.50E-04 | 7.2528751 | 1.518391 | 1.3023749 | Armcx1 |
| 10385466 | 0.00853 | 1.51E-04 | 7.2504922 | 1.516248 | 1.48974944 | Sgcd |
| 10358623 | 0.00853 | 1.51E-04 | 7.2491854 | 1.515072 | 2.1260662 | Hmcn1 |
| 10530319 | 0.00861 | 1.52E-04 | 7.2375875 | 1.504628 | 1.15851306 | Atp8a1 |
| 10384940 | 0.00862 | 1.53E-04 | 7.2307799 | 1.498491 | 1.63143859 | Erlec1 |
| 10565152 | 0.00862 | 1.53E-04 | 7.2305026 | 1.498241 | 1.16712337 | Homer2 |
| 10602827 | 0.00862 | 1.54E-04 | 7.2286611 | 1.49658 | 1.26028697 | A830080D01Rik |
| 10396008 | 0.00868 | 1.55E-04 | 7.2177191 | 1.486701 | 1.35795015 | Prpf39 |
| 10448195 | 0.00869 | 1.56E-04 | 7.2121681 | 1.481684 | 1.68611304 | Zfp942 |
| 10395831 | 0.00869 | 1.56E-04 | 7.2099907 | 1.479716 | 1.41638027 | Brms1l |
| 10377405 | 0.0087 | 1.56E-04 | -7.207026 | 1.477034 | -1.1517802 | Aurkb |
| 10359582 | 0.0087 | 1.57E-04 | 7.2062572 | 1.476339 | 1.64125435 | Fmo2 |
| 10436600 | 0.00877 | 1.59E-04 | 7.1907409 | 1.462286 | 1.95723384 | Mir99a |
| 10552874 | 0.00878 | 1.59E-04 | 7.1867614 | 1.458678 | 1.14032342 | Tead2 |
| 10544523 | 0.00878 | 1.59E-04 | -7.1862316 | 1.458197 | -2.0013263 | Rny1 |
| 10369481 | 0.00882 | 1.62E-04 | 7.1704643 | 1.443881 | 1.26124353 | H2afy2 |
| 10491014 | 0.00882 | 1.62E-04 | 7.1660692 | 1.439885 | 1.012678 | Hltf |
| 10408202 | 0.00882 | 1.62E-04 | -7.1650327 | 1.438942 | -1.15648955 | Hist1h3a///Hist2h3b///Hist1h3i///Hist1h3h///Hist1h3e///Hist1h3b///Hist1h3d///Hist1h3c///Hist1h3f///Hist1h3g///Hist2h3c2///Hist2h3c1///Hist1h3d |
| 10358648 | 0.00882 | 1.63E-04 | 7.1614394 | 1.435673 | 1.43449092 | Hmcn1 |
| 10431915 | 0.00882 | 1.63E-04 | 7.159078 | 1.433524 | 1.37961933 | Slc38a4 |
| 10479274 | 0.00882 | 1.64E-04 | -7.1567919 | 1.431443 | -1.18590759 | Cdh4 |
| 10499839 | 0.00882 | 1.64E-04 | 7.155101 | 1.429904 | 1.34153229 | Snapin |
| 10430825 | 0.00882 | 1.64E-04 | -7.1520997 | 1.42717 | -1.12399782 | Cenpm |
| 10412345 | 0.00882 | 1.65E-04 | 7.1508211 | 1.426005 | 1.54725579 | Parp8 |
| 10371607 | 0.00883 | 1.65E-04 | 7.148821 | 1.424182 | 1.73452821 | Dram1 |
| 10406482 | 0.00884 | 1.65E-04 | 7.1453917 | 1.421056 | 1.14617068 | Ccnh |
| 10593024 | 0.00885 | 1.66E-04 | -7.1429087 | 1.418792 | -1.60480291 | Cd3e |
| 10422194 | 0.00887 | 1.66E-04 | 7.1375655 | 1.413916 | 1.53522575 | Rbm26 |
| 10410124 | 0.00887 | 1.67E-04 | 7.1368952 | 1.413305 | 1.33447391 | Ctsl |
| 10516229 | 0.0089 | 1.68E-04 | 7.1285981 | 1.405727 | 1.15291165 | Utp11l |
| 10439021 | 0.0089 | 1.68E-04 | 7.1279082 | 1.405096 | 1.19347005 | Senp5 |
| 10505954 | 0.00902 | 1.71E-04 | 7.1055966 | 1.384678 | 1.15026158 | Tek |
| 10357043 | 0.00907 | 1.73E-04 | 7.0933224 | 1.37342 | 1.36908049 | Bcl2 |
| 10600131 | 0.00907 | 1.73E-04 | -7.0930132 | 1.373136 | -3.88516481 | Xlr4a///Xlr4c///Xlr4b |
| 10565330 | 0.00908 | 1.74E-04 | 7.0898492 | 1.370231 | 1.43083149 | Zfand6 |
| 10353871 | 0.00908 | 1.74E-04 | 7.0890198 | 1.36947 | 1.04758908 | Lman2l |
| 10579894 | 0.00912 | 1.75E-04 | 7.0793794 | 1.36061 | 1.16644955 | Hhip |
| 10350516 | 0.00912 | 1.75E-04 | -7.0787741 | 1.360054 | -1.29484051 | Ptgs2 |
| 10469425 | 0.00915 | 1.76E-04 | 7.0738039 | 1.355482 | 1.3260179 | Arl5b |
| 10358551 | 0.00917 | 1.77E-04 | 7.0694962 | 1.351517 | 1.72301455 | Hmcn1 |
| 10532150 | 0.00922 | 1.78E-04 | 7.0607206 | 1.343432 | 1.41710138 | Fam69a |
| 10354868 | 0.00924 | 1.79E-04 | 7.056326 | 1.33938 | 1.09508517 | Fam126b |
| 10608001 | 0.00925 | 1.80E-04 | 7.0491305 | 1.332741 | 1.52499932 | Eif2s3y |
| 10344981 | 0.00926 | 1.81E-04 | 7.047069 | 1.330838 | 1.99737675 | Pi15 |
| 10410259 | 0.00928 | 1.82E-04 | 7.0391562 | 1.323528 | 1.50103689 | Uqcrb |
| 10484371 | 0.0093 | 1.82E-04 | 7.0357391 | 1.320369 | 1.05997228 | Calcrl |
| 10358650 | 0.0093 | 1.84E-04 | 7.0292176 | 1.314336 | 1.37020947 | Hmcn1 |
| 10421723 | 0.0093 | 1.84E-04 | 7.0269897 | 1.312274 | 1.00036048 | Dnajc15 |
| 10358525 | 0.0093 | 1.85E-04 | 7.0230316 | 1.308608 | 2.19352771 | Hmcn1 |
| 10358561 | 0.0093 | 1.85E-04 | 7.0214371 | 1.307131 | 1.75532079 | Hmcn1 |
| 10500976 | 0.00934 | 1.86E-04 | 7.013487 | 1.299763 | 1.15269476 | Rap1a |
| 10369702 | 0.00935 | 1.87E-04 | 7.0063876 | 1.293176 | 1.28170754 | Tet1 |
| 10372716 | 0.00935 | 1.88E-04 | 7.0043222 | 1.291259 | 1.48308369 | Rap1b |
| 10462918 | 0.00935 | 1.88E-04 | 7.0004509 | 1.287664 | 1.00385932 | Slc35g1 |
| 10511180 | 0.00935 | 1.89E-04 | 6.9994077 | 1.286695 | 1.28788537 | Mxra8 |
| 10400538 | 0.00935 | 1.89E-04 | 6.9957207 | 1.283268 | 1.33180014 | Trappc6b |
| 10381298 | 0.00935 | 1.90E-04 | 6.993232 | 1.280955 | 1.37524665 | Ramp2 |
| 10356764 | 0.00935 | 1.90E-04 | -6.9900078 | 1.277956 | -1.22881055 | 2310007B03Rik |
| 10595768 | 0.00935 | 1.91E-04 | -6.987324 | 1.27546 | -1.01708156 | Pls1 |
| 10506767 | 0.00935 | 1.91E-04 | 6.9843681 | 1.272709 | 1.06876775 | Echdc2 |
| 10454099 | 0.00935 | 1.91E-04 | 6.9843459 | 1.272688 | 1.31188387 | Morf4l1-ps1///Morf4l1 |
| 10378848 | 0.00935 | 1.91E-04 | 6.9829524 | 1.271391 | 1.73428372 | Hsp90aa1 |
| 10597531 | 0.00935 | 1.92E-04 | 6.9822456 | 1.270733 | 1.02783894 | Rbms3 |
| 10358575 | 0.00935 | 1.92E-04 | 6.9813546 | 1.269903 | 1.06315486 | Hmcn1 |
| 10514221 | 0.00935 | 1.92E-04 | 6.9795971 | 1.268267 | 1.16064193 | Plin2 |
| 10520124 | 0.00935 | 1.92E-04 | 6.9788809 | 1.267599 | 1.04480257 | Sumo2 |
| 10432294 | 0.00941 | 1.94E-04 | 6.9688104 | 1.258213 | 1.19019101 | Kmt2d |
| 10349157 | 0.00942 | 1.94E-04 | -6.9660438 | 1.255632 | -1.1307107 | Serpinb2 |
| 10500543 | 0.00942 | 1.95E-04 | 6.9629932 | 1.252785 | 2.3077551 | Hmgb1-rs17///Gm5176///4932431P20Rik///Hmgb1///Gm12568 |
| 10369102 | 0.00942 | 1.95E-04 | 6.962359 | 1.252193 | 1.05605805 | Cep85l |
| 10574159 | 0.00942 | 1.96E-04 | -6.9570537 | 1.247239 | -1.01420908 | Nlrc5 |
| 10357788 | 0.00942 | 1.96E-04 | -6.9545019 | 1.244855 | -1.12112039 | Ppp1r15b |
| 10561842 | 0.00943 | 1.97E-04 | 6.9512167 | 1.241785 | 1.3513708 | Capns1 |
| 10474793 | 0.00944 | 1.98E-04 | -6.9483099 | 1.239067 | -1.0338778 | Pak6 |
| 10567022 | 0.00946 | 1.99E-04 | 6.9392953 | 1.230633 | 1.17535244 | Btbd10 |
| 10407833 | 0.00946 | 2.00E-04 | 6.9339774 | 1.225652 | 1.66422137 | Ggps1 |
| 10574157 | 0.00946 | 2.01E-04 | -6.9312227 | 1.223071 | -1.22508957 | Nlrc5 |
| 10584628 | 0.00946 | 2.01E-04 | -6.930823 | 1.222696 | -1.64208687 | Thy1 |
| 10529758 | 0.0095 | 2.02E-04 | 6.9245744 | 1.216838 | 1.24462285 | Bod1l |
| 10404026 | 0.00953 | 2.03E-04 | -6.9187925 | 1.211412 | -1.29442845 | Hist1h2al///Hist1h2ao///Hist2h2ab///Hist2h2aa2///Hist1h2ai///Hist2h2ac///Hist1h2af///Hist1h2ab///Hist1h2ap///Hist1h2an///Hist1h2ak///Hist1h2ah///Hist1h2ag///Hist1h2ae///Hist1h2ad///Hist1h2ac///Hist1h2aa///Hist3h2a///H2afj///H2afx///Hist2h2aa1///Hist1h3d |
| 10521537 | 0.00957 | 2.04E-04 | 6.9114718 | 1.204537 | 1.85066977 | Cytl1 |
| 10508651 | 0.00961 | 2.06E-04 | 6.9017779 | 1.195423 | 1.07904818 | Sdc3 |
| 10468869 | 0.00974 | 2.10E-04 | 6.8819623 | 1.176758 | 1.15497161 | Prdx3 |
| 10605421 | 0.00975 | 2.10E-04 | 6.8790832 | 1.174042 | 1.19701435 | Cmc4///Mtcp1 |
| 10407814 | 0.00975 | 2.11E-04 | 6.8767355 | 1.171826 | 1.25474855 | Tbce |
| 10556820 | 0.0098 | 2.13E-04 | 6.8666032 | 1.162257 | 1.39008443 | Tmem159 |
| 10378568 | 0.00981 | 2.14E-04 | 6.8594233 | 1.155469 | 1.98659732 | Mir22hg///Mir22 |
| 10358613 | 0.00983 | 2.15E-04 | 6.8536244 | 1.149982 | 2.25168078 | Hmcn1 |
| 10489364 | 0.00985 | 2.16E-04 | 6.8499371 | 1.146491 | 1.35022526 | Oser1 |
| 10604230 | 0.00987 | 2.17E-04 | 6.8446811 | 1.141511 | 1.29508835 | Ap3s1 |
| 10445338 | 0.00987 | 2.17E-04 | 6.8436042 | 1.140491 | 1.6531112 | Enpp5 |
| 10432957 | 0.00987 | 2.18E-04 | -6.8399777 | 1.137052 | -1.15461495 | Itgb7 |
| 10554863 | 0.00987 | 2.18E-04 | 6.8395416 | 1.136639 | 1.23879497 | Sytl2 |
| 10546454 | 0.00987 | 2.19E-04 | 6.8377145 | 1.134906 | 1.81688054 | Adamts9 |
| 10577230 | 0.00994 | 2.21E-04 | 6.8260638 | 1.123846 | 1.12579895 | Erich1 |
| 10601328 | 0.00997 | 2.22E-04 | 6.8219277 | 1.119916 | 2.32566995 | Uprt |
| 10392449 | 0.01004 | 2.25E-04 | 6.8056345 | 1.104414 | 1.04088532 | Wipi1 |
| 10497487 | 0.01004 | 2.26E-04 | 6.8026034 | 1.101526 | 1.04700699 | Naaladl2 |
| 10416411 | 0.01005 | 2.28E-04 | 6.794323 | 1.093632 | 1.00639458 | Esd |
| 10365145 | 0.01005 | 2.30E-04 | 6.7854838 | 1.085196 | 1.01828502 | Tle2 |
| 10474577 | 0.01005 | 2.30E-04 | 6.7851291 | 1.084857 | 1.3825073 | Slc12a6///Katnbl1 |
| 10602307 | 0.01005 | 2.30E-04 | -6.7837757 | 1.083564 | -3.0866553 | Gm15097///Gm15127///Luzp4///Gm15107///Gm15128///Gm15091///Gm10439///Ott |
| 10360542 | 0.01005 | 2.31E-04 | 6.7784945 | 1.078518 | 1.32742659 | AI503316 |
| 10538408 | 0.01005 | 2.31E-04 | 6.7770048 | 1.077094 | 1.06579856 | Mturn |
| 10571241 | 0.01008 | 2.32E-04 | 6.7720997 | 1.072403 | 1.1284086 | Purg |
| 10385761 | 0.01008 | 2.33E-04 | 6.7675992 | 1.068097 | 1.15142623 | Ube2b |
| 10474243 | 0.01009 | 2.34E-04 | 6.7633845 | 1.064062 | 1.32551274 | Cstf3 |
| 10404389 | 0.01011 | 2.36E-04 | -6.7578409 | 1.058751 | -1.24653586 | Irf4 |
| 10354432 | 0.01011 | 2.36E-04 | 6.7578401 | 1.05875 | 1.24826279 | Myo1b |
| 10427807 | 0.01011 | 2.36E-04 | 6.755957 | 1.056945 | 1.23459193 | Sub1 |
| 10407012 | 0.01011 | 2.36E-04 | 6.7545909 | 1.055635 | 1.05354643 | Srek1ip1 |
| 10506004 | 0.01015 | 2.37E-04 | 6.7490227 | 1.050295 | 1.41737668 | Hook1 |
| 10547227 | 0.01019 | 2.40E-04 | 6.7394499 | 1.041104 | 1.92304618 | Ret |
| 10362201 | 0.01019 | 2.40E-04 | 6.7372918 | 1.039031 | 1.40096733 | Ctgf |
| 10466938 | 0.01019 | 2.41E-04 | 6.7352777 | 1.037095 | 1.17371465 | Plgrkt |
| 10513739 | 0.01019 | 2.41E-04 | 6.7352537 | 1.037072 | 1.80782263 | Tnc |
| 10354220 | 0.01019 | 2.41E-04 | 6.7348732 | 1.036706 | 1.2899628 | Mfsd9 |
| 10441973 | 0.0102 | 2.41E-04 | 6.732174 | 1.034111 | 1.23027022 | Tbp |
| 10397083 | 0.01023 | 2.43E-04 | 6.7238179 | 1.026072 | 1.25137084 | Rbm25 |
| 10606858 | 0.01025 | 2.46E-04 | 6.7104373 | 1.013181 | 1.15633584 | Tceal8 |
| 10357242 | 0.01025 | 2.46E-04 | 6.7103513 | 1.013098 | 1.09111995 | Dbi |
| 10528102 | 0.01025 | 2.46E-04 | 6.7094925 | 1.01227 | 1.32233808 | Crot |
| 10498415 | 0.01029 | 2.48E-04 | 6.7008932 | 1.003972 | 1.14000581 | Dhx36 |
| 10468287 | 0.01041 | 2.52E-04 | 6.6860198 | 0.989599 | 1.97045688 | Usmg5 |
| 10530380 | 0.01041 | 2.52E-04 | 6.6854257 | 0.989024 | 1.10581761 | Gnpda2 |
| 10389010 | 0.01041 | 2.53E-04 | 6.6796257 | 0.983411 | 1.04912579 | 5730455P16Rik |
| 10407286 | 0.01041 | 2.53E-04 | 6.6793363 | 0.983131 | 1.12184778 | BC067074 |
| 10509030 | 0.01045 | 2.55E-04 | -6.6717187 | 0.975753 | -1.12984253 | Runx3 |
| 10379689 | 0.01056 | 2.59E-04 | 6.6574886 | 0.96195 | 1.30106493 | Taf15 |
| 10455346 | 0.01059 | 2.60E-04 | 6.653472 | 0.958049 | 1.11608459 | Tcerg1 |
| 10600524 | 0.01061 | 2.62E-04 | 6.645061 | 0.949874 | 1.92472289 | Vbp1 |
| 10596259 | 0.01061 | 2.62E-04 | 6.6443827 | 0.949214 | 1.99097131 | Dnajc13 |
| 10481845 | 0.01061 | 2.62E-04 | 6.6439328 | 0.948777 | 1.11845837 | Mvb12b |
| 10397633 | 0.01061 | 2.64E-04 | 6.6358962 | 0.940956 | 1.42331601 | Flrt2 |
| 10481868 | 0.01061 | 2.64E-04 | 6.635173 | 0.940252 | 3.12629487 | LOC102633596///Dnajb6 |
| 10454807 | 0.01061 | 2.64E-04 | -6.6348734 | 0.939961 | -1.27388824 | Snora74a |
| 10358515 | 0.01061 | 2.65E-04 | 6.6340934 | 0.939201 | 1.81067739 | Hmcn1 |
| 10350247 | 0.01061 | 2.65E-04 | 6.6328871 | 0.938026 | 1.24863393 | Kif21b |
| 10508089 | 0.01061 | 2.65E-04 | 6.6326005 | 0.937747 | 1.16724636 | Mrps15 |
| 10358611 | 0.01061 | 2.65E-04 | 6.6325707 | 0.937718 | 1.66968565 | Hmcn1 |
| 10497066 | 0.01066 | 2.68E-04 | 6.6214605 | 0.926889 | 1.22503939 | Zranb2 |
| 10472538 | 0.01066 | 2.68E-04 | 6.6210458 | 0.926484 | 1.3782071 | Dhrs9 |
| 10454445 | 0.01068 | 2.69E-04 | -6.6172017 | 0.922733 | -1.11208515 | B930094E09Rik |
| 10366052 | 0.01076 | 2.71E-04 | 6.6084673 | 0.914204 | 1.43503276 | Kitl |
| 10427814 | 0.01076 | 2.72E-04 | -6.6064665 | 0.912249 | -1.2430381 | Golph3 |
| 10480121 | 0.01076 | 2.72E-04 | 6.6053135 | 0.911122 | 1.04652094 | Fam188a |
| 10490212 | 0.01081 | 2.74E-04 | 6.5985448 | 0.904503 | 1.2492193 | Ctsz |
| 10376929 | 0.01082 | 2.75E-04 | 6.5918138 | 0.897915 | 1.12307853 | Tvp23b |
| 10360743 | 0.01082 | 2.77E-04 | -6.5856721 | 0.891899 | -1.07278362 | 7120482A17Rik///Rnu6 |
| 10488303 | 0.01082 | 2.77E-04 | 6.5852885 | 0.891523 | 1.02795128 | Crnkl1 |
| 10461439 | 0.01082 | 2.77E-04 | 6.5851305 | 0.891368 | 1.35118565 | Fads1 |
| 10389795 | 0.01082 | 2.78E-04 | 6.5835509 | 0.88982 | 2.39468782 | Stxbp4 |
| 10568553 | 0.01082 | 2.78E-04 | -6.5825336 | 0.888823 | -1.09247722 | Chst15 |
| 10354832 | 0.01084 | 2.79E-04 | 6.5791461 | 0.885501 | 1.08620201 | Ppil3 |
| 10491056 | 0.01086 | 2.80E-04 | 6.5730469 | 0.879517 | 1.27970453 | Tbl1xr1 |
| 10483000 | 0.01086 | 2.81E-04 | 6.5689257 | 0.87547 | 1.18500144 | Itgb6 |
| 10581902 | 0.01086 | 2.82E-04 | 6.56864 | 0.87519 | 1.42319045 | Cfdp1 |
| 10567010 | 0.01086 | 2.82E-04 | 6.568269 | 0.874825 | 1.11789515 | Dkk3 |
| 10409709 | 0.01088 | 2.83E-04 | 6.5648208 | 0.871438 | 1.0013803 | Mir7-1 |
| 10466800 | 0.01089 | 2.84E-04 | 6.5608017 | 0.867488 | 1.0934356 | Pgm5 |
| 10607183 | 0.01092 | 2.86E-04 | 6.5537635 | 0.860565 | 1.06624187 | Lhfpl1 |
| 10458906 | 0.01095 | 2.87E-04 | 6.5495351 | 0.856403 | 1.20527869 | Ppic |
| 10364051 | 0.01095 | 2.87E-04 | 6.5468412 | 0.85375 | 1.01303653 | Snrpd3 |
| 10464202 | 0.01095 | 2.88E-04 | 6.5463726 | 0.853289 | 1.033513 | Vwa2 |
| 10571601 | 0.01095 | 2.88E-04 | 6.5447207 | 0.851661 | 1.64056938 | Pdlim3 |
| 10456184 | 0.01096 | 2.89E-04 | -6.5410701 | 0.848064 | -1.15594621 | Apcdd1 |
| 10565567 | 0.01097 | 2.91E-04 | 6.5353871 | 0.842461 | 1.35819604 | 4632427E13Rik |
| 10479228 | 0.01097 | 2.91E-04 | 6.5345814 | 0.841666 | 2.49765008 | Zfp971 |
| 10605370 | 0.01097 | 2.91E-04 | 6.5328383 | 0.839946 | 1.35046896 | Mpp1 |
| 10401114 | 0.01097 | 2.91E-04 | 6.5326743 | 0.839784 | 1.34216674 | Rab15 |
| 10384486 | 0.01097 | 2.92E-04 | 6.5314023 | 0.838529 | 1.63848844 | Etaa1 |
| 10344789 | 0.01099 | 2.93E-04 | 6.5266131 | 0.833801 | 1.39763521 | Cspp1 |
| 10436253 | 0.01099 | 2.94E-04 | 6.5243074 | 0.831524 | 1.02275571 | Senp7 |
| 10406530 | 0.01101 | 2.95E-04 | 6.5211877 | 0.828442 | 1.18998843 | Tmem167 |
| 10468722 | 0.01103 | 2.95E-04 | 6.5185072 | 0.825793 | 1.07195907 | Gfra1 |
| 10578619 | 0.0111 | 2.98E-04 | 6.5078295 | 0.81523 | 1.13714537 | Cdkn2aip |
| 10561345 | 0.01111 | 2.99E-04 | 6.5055374 | 0.812961 | 1.01313602 | Zfp780b |
| 10598236 | 0.01113 | 3.00E-04 | 6.5014804 | 0.808943 | 1.3036249 | Nudt11 |
| 10496796 | 0.01113 | 3.00E-04 | 6.5011691 | 0.808634 | 1.4252836 | Ssx2ip |
| 10534456 | 0.01124 | 3.04E-04 | 6.4870715 | 0.794654 | 1.15730955 | Hip1 |
| 10454369 | 0.01124 | 3.05E-04 | 6.4860227 | 0.793613 | 1.12702552 | Fhod3 |
| 10359339 | 0.01133 | 3.09E-04 | 6.4710897 | 0.778774 | 1.01135302 | Rabgap1l |
| 10352194 | 0.0115 | 3.16E-04 | 6.4485631 | 0.756336 | 1.16399766 | Cdc42bpa |
| 10551760 | 0.0115 | 3.17E-04 | 6.4465773 | 0.754355 | 1.47017686 | Zfp84 |
| 10375893 | 0.0115 | 3.17E-04 | 6.4463508 | 0.754129 | 1.28514568 | Sar1b |
| 10528207 | 0.0115 | 3.17E-04 | 6.4445072 | 0.752289 | 1.30861155 | Cd36 |
| 10554693 | 0.01154 | 3.19E-04 | 6.4369145 | 0.744707 | 1.07033414 | Stard5///Stard5 |
| 10482177 | 0.01169 | 3.27E-04 | 6.414106 | 0.721886 | 1.36330003 | Strbp |
| 10528484 | 0.01175 | 3.28E-04 | 6.408355 | 0.716121 | 1.29028239 | Srpk2 |
| 10388520 | 0.01178 | 3.30E-04 | 6.4045444 | 0.712299 | 1.03058404 | Glod4 |
| 10519392 | 0.01181 | 3.31E-04 | 6.3998849 | 0.707623 | 1.1746814 | Krit1 |
| 10490246 | 0.01181 | 3.31E-04 | 6.3993413 | 0.707077 | 1.6285951 | Gm14326///Gm14403 |
| 10482323 | 0.01181 | 3.33E-04 | 6.395424 | 0.703144 | 1.63892519 | Ppp6c |
| 10394498 | 0.01181 | 3.33E-04 | 6.3944181 | 0.702133 | 1.39629722 | Wdr35 |
| 10472757 | 0.01181 | 3.33E-04 | 6.3940526 | 0.701766 | 1.24650065 | Cybrd1 |
| 10357155 | 0.01182 | 3.34E-04 | 6.390014 | 0.697708 | 1.3753469 | Inhbb///Inhbb |
| 10598839 | 0.01185 | 3.36E-04 | 6.3858182 | 0.693489 | 1.62833969 | Rp2 |
| 10394381 | 0.01185 | 3.36E-04 | 6.3855127 | 0.693182 | 1.11131244 | Atad2b |
| 10410209 | 0.01185 | 3.36E-04 | 6.3845188 | 0.692182 | 1.0112462 | Rybp |
| 10508721 | 0.01193 | 3.39E-04 | -6.3749793 | 0.68258 | -1.01433894 | Snora44 |
| 10485633 | 0.01193 | 3.41E-04 | 6.3689468 | 0.676502 | 2.06334623 | Gm10796 |
| 10424467 | 0.01193 | 3.42E-04 | 6.3679374 | 0.675485 | 1.00476184 | Phf20l1 |
| 10373902 | 0.01194 | 3.43E-04 | 6.3644692 | 0.671988 | 1.23369826 | Gatsl3 |
| 10505630 | 0.01199 | 3.44E-04 | 6.3595499 | 0.667025 | 1.33901962 | Snapc3 |
| 10410295 | 0.012 | 3.46E-04 | 6.3553573 | 0.662793 | 1.33414584 | Zfp595 |
| 10488291 | 0.012 | 3.46E-04 | 6.3540242 | 0.661447 | 1.62606399 | Rbbp9 |
| 10457862 | 0.01211 | 3.51E-04 | 6.3394637 | 0.646729 | 1.24706132 | Rprd1a |
| 10546066 | 0.01211 | 3.52E-04 | 6.33826 | 0.64551 | 1.16196837 | Isy1 |
| 10469046 | 0.01211 | 3.52E-04 | 6.338091 | 0.645339 | 1.61307058 | Phyh |
| 10509023 | 0.0122 | 3.57E-04 | 6.3240772 | 0.631145 | 1.00899125 | Syf2 |
| 10542079 | 0.01221 | 3.59E-04 | -6.3181535 | 0.625137 | -1.24929569 | Foxm1 |
| 10447897 | 0.01221 | 3.59E-04 | 6.3170052 | 0.623972 | 1.05192677 | Wtap |
| 10362837 | 0.01221 | 3.60E-04 | 6.3155897 | 0.622535 | 1.57015047 | Sec63 |
| 10442236 | 0.01221 | 3.60E-04 | 6.315567 | 0.622512 | 1.84255865 | Gm10509///Zfp983 |
| 10476560 | 0.01227 | 3.64E-04 | 6.3036601 | 0.610418 | 1.15372263 | Ism1 |
| 10431802 | 0.0123 | 3.66E-04 | 6.2981852 | 0.60485 | 1.21913413 | Twf1 |
| 10375216 | 0.0123 | 3.67E-04 | 6.2954006 | 0.602017 | 1.04766801 | Pank3 |
| 10397068 | 0.0123 | 3.67E-04 | 6.2946136 | 0.601216 | 1.34362417 | Rbm25 |
| 10475456 | 0.0123 | 3.68E-04 | 6.2921211 | 0.598679 | 1.16971022 | Duox1 |
| 10417676 | 0.0123 | 3.69E-04 | 6.2883836 | 0.594873 | 1.11581608 | Thoc7 |
| 10585982 | 0.0123 | 3.70E-04 | 6.2879333 | 0.594415 | 1.28879139 | Myo9a |
| 10467688 | 0.01235 | 3.71E-04 | 6.2826834 | 0.589065 | 1.38451347 | Exosc1 |
| 10474006 | 0.01236 | 3.72E-04 | 6.28003 | 0.58636 | 1.11199786 | Phf21a |
| 10360972 | 0.01239 | 3.74E-04 | 6.2759691 | 0.582218 | 1.16531298 | Kcnk2 |
| 10464169 | 0.01239 | 3.74E-04 | -6.2759581 | 0.582207 | -1.07091098 | 1700010L13Rik |
| 10475437 | 0.01243 | 3.76E-04 | 6.2698777 | 0.576001 | 1.2486939 | Sord |
| 10344801 | 0.01248 | 3.79E-04 | 6.2630326 | 0.569009 | 1.45640782 | Cspp1 |
| 10416793 | 0.01263 | 3.84E-04 | 6.248002 | 0.553634 | 1.19567715 | Uchl3 |
| 10603833 | 0.01263 | 3.85E-04 | 6.2450167 | 0.550577 | 1.80428543 | Usmg5 |
| 10509901 | 0.01263 | 3.86E-04 | 6.2446344 | 0.550185 | 1.33446129 | Mfap2 |
| 10361265 | 0.01263 | 3.86E-04 | 6.2445582 | 0.550107 | 1.71073125 | Mir205 |
| 10511755 | 0.01264 | 3.87E-04 | 6.2418085 | 0.54729 | 1.17379664 | Wwp1 |
| 10497711 | 0.01264 | 3.88E-04 | -6.2394501 | 0.544873 | -1.1771231 | Mrpl47 |
| 10511617 | 0.01264 | 3.88E-04 | 6.238479 | 0.543877 | 1.21926478 | Fam92a |
| 10400649 | 0.01276 | 3.93E-04 | 6.2254962 | 0.530556 | 1.50239739 | Pole2 |
| 10364038 | 0.01277 | 3.94E-04 | 6.2228725 | 0.527861 | 1.30340393 | Upb1 |
| 10584317 | 0.01277 | 3.94E-04 | 6.2227289 | 0.527714 | 1.31129432 | Esam |
| 10461898 | 0.01279 | 3.95E-04 | 6.2196027 | 0.524502 | 1.0963112 | Rfk |
| 10546706 | 0.01283 | 3.97E-04 | 6.2154429 | 0.520225 | 1.33506729 | Rybp |
| 10450646 | 0.01283 | 3.97E-04 | 6.2142921 | 0.519042 | 1.20218204 | Rbx1 |
| 10529977 | 0.01285 | 3.99E-04 | 6.2109646 | 0.515619 | 1.15935949 | Ppargc1a |
| 10528120 | 0.01287 | 4.00E-04 | 6.2079247 | 0.512491 | 1.05409255 | Dmtf1 |
| 10490672 | 0.01287 | 4.01E-04 | 6.2061858 | 0.510701 | 1.25677444 | Arfrp1 |
| 10396645 | 0.01287 | 4.02E-04 | 6.2038355 | 0.508281 | 1.24866684 | Zbtb1 |
| 10455015 | 0.01287 | 4.03E-04 | -6.2014617 | 0.505836 | -1.15789093 | Vaultrc5 |
| 10474411 | 0.01287 | 4.03E-04 | 6.1994859 | 0.5038 | 1.73447035 | Lin7c |
| 10396193 | 0.0129 | 4.05E-04 | 6.1964778 | 0.5007 | 1.10597574 | Psma3 |
| 10517250 | 0.01292 | 4.06E-04 | -6.1929978 | 0.497112 | -1.21381769 | Extl1 |
| 10372478 | 0.01292 | 4.06E-04 | 6.1925771 | 0.496678 | 1.02287211 | Rab21 |
| 10396161 | 0.01292 | 4.07E-04 | 6.1911486 | 0.495204 | 1.62662133 | Tmx1 |
| 10411915 | 0.01292 | 4.07E-04 | 6.1902154 | 0.494242 | 1.46202222 | Ppwd1 |
| 10407940 | 0.01304 | 4.15E-04 | -6.1704806 | 0.473856 | -1.59262106 | Trgv2///Tcrg-V6///Tcrg-V4///Trgv2 |
| 10410351 | 0.01304 | 4.16E-04 | 6.1694878 | 0.472829 | 1.92055801 | Zfp729b |
| 10552440 | 0.01304 | 4.16E-04 | 6.1687517 | 0.472067 | 1.56287112 | Zfp719 |
| 10441539 | 0.01304 | 4.16E-04 | 6.1682328 | 0.471531 | 3.22435052 | Rnaset2a///Rnaset2b///Rnaset2b |
| 10538459 | 0.01304 | 4.16E-04 | 6.1679123 | 0.471199 | 2.09940377 | Aqp1 |
| 10506274 | 0.01304 | 4.16E-04 | -6.1672822 | 0.470547 | -1.23751644 | Dnajc6 |
| 10599222 | 0.01304 | 4.17E-04 | 6.1661637 | 0.46939 | 1.55654341 | Ube2a |
| 10397507 | 0.01304 | 4.19E-04 | 6.1601673 | 0.463181 | 1.11536879 | Gstz1 |
| 10407042 | 0.01304 | 4.20E-04 | 6.1597057 | 0.462703 | 1.2124981 | Dimt1 |
| 10571978 | 0.01304 | 4.20E-04 | 6.159415 | 0.462402 | 1.97973624 | Cbr4 |
| 10456887 | 0.01304 | 4.20E-04 | -6.1583494 | 0.461298 | -1.13627877 | Loxhd1 |
| 10462237 | 0.01304 | 4.20E-04 | 6.1582551 | 0.461201 | 1.01544012 | Smarca2 |
| 10433782 | 0.01304 | 4.20E-04 | 6.1576762 | 0.460601 | 1.06041587 | Efcab1 |
| 10570764 | 0.01305 | 4.23E-04 | 6.1521168 | 0.454839 | 1.01284743 | Alg11 |
| 10596261 | 0.01306 | 4.24E-04 | 6.1488529 | 0.451454 | 2.09363363 | Dnajc13 |
| 10395005 | 0.01307 | 4.25E-04 | 6.1462712 | 0.448776 | 1.34011741 | Kidins220 |
| 10489041 | 0.01307 | 4.25E-04 | 6.1461494 | 0.44865 | 1.24743656 | Pdcd10 |
| 10506118 | 0.01309 | 4.27E-04 | 6.1421225 | 0.44447 | 1.19559218 | Usp1 |
| 10475247 | 0.01324 | 4.34E-04 | 6.1258249 | 0.427533 | 1.13484877 | Tmem62 |
| 10492355 | 0.01334 | 4.39E-04 | 6.1142701 | 0.415502 | 1.77213576 | Mme |
| 10604187 | 0.01336 | 4.40E-04 | 6.1114854 | 0.412601 | 1.04066327 | Lamp2 |
| 10511382 | 0.01339 | 4.42E-04 | 6.1073193 | 0.408257 | 1.15913218 | Nsmaf |
| 10489239 | 0.0134 | 4.43E-04 | -6.1058852 | 0.406761 | -1.09669017 | Snhg17 |
| 10515803 | 0.01345 | 4.46E-04 | -6.0987156 | 0.399279 | -1.24891346 | Cfap57 |
| 10504817 | 0.01345 | 4.47E-04 | 6.0974508 | 0.397959 | 1.56828087 | Tgfbr1 |
| 10353219 | 0.01348 | 4.48E-04 | 6.0949305 | 0.395327 | 3.35174985 | Sumo2 |
| 10413265 | 0.01348 | 4.49E-04 | 6.0930827 | 0.393396 | 1.07811088 | Duxbl2///Duxbl1 |
| 10357917 | 0.01349 | 4.50E-04 | 6.0908828 | 0.391097 | 1.0091903 | Tmem183a |
| 10523060 | 0.01351 | 4.52E-04 | 6.0852813 | 0.385241 | 1.11943107 | Gm9958 |
| 10528268 | 0.01351 | 4.53E-04 | 6.0836052 | 0.383488 | 1.45191286 | Ptpn12 |
| 10410302 | 0.01352 | 4.54E-04 | 6.0813352 | 0.381113 | 1.20298059 | Zfp953 |
| 10485635 | 0.01362 | 4.60E-04 | 6.0679909 | 0.367137 | 1.29399682 | Eif3m |
| 10357003 | 0.01368 | 4.63E-04 | 6.0612824 | 0.360102 | 1.76994631 | Rnf152 |
| 10579049 | 0.01368 | 4.64E-04 | 6.060741 | 0.359534 | 1.56922334 | Gm10033 |
| 10391178 | 0.01372 | 4.65E-04 | 6.057157 | 0.355772 | 1.00899475 | Dnajc7 |
| 10498775 | 0.01381 | 4.70E-04 | 6.0463727 | 0.344444 | 1.56968096 | Golim4 |
| 10574098 | 0.01382 | 4.71E-04 | -6.0444565 | 0.342429 | -1.01528246 | Nlrc5 |
| 10534142 | 0.01393 | 4.78E-04 | 6.0312309 | 0.328511 | 1.18783949 | Sbds |
| 10358672 | 0.01393 | 4.78E-04 | 6.0310942 | 0.328367 | 1.55139616 | Hmcn1 |
| 10537353 | 0.01395 | 4.79E-04 | 6.0274553 | 0.324533 | 1.01470309 | Ttc26 |
| 10596257 | 0.01395 | 4.80E-04 | 6.0265596 | 0.323589 | 1.87362793 | Dnajc13 |
| 10381122 | 0.01398 | 4.81E-04 | 6.0235716 | 0.32044 | 1.28418354 | Fkbp10 |
| 10486026 | 0.01403 | 4.84E-04 | 6.0190086 | 0.315627 | 1.0844011 | Zfp770 |
| 10556491 | 0.01404 | 4.85E-04 | 6.0166065 | 0.313093 | 1.37268184 | Far1 |
| 10471715 | 0.01404 | 4.85E-04 | 6.0156708 | 0.312105 | 1.79302163 | Mrrf |
| 10363475 | 0.01404 | 4.86E-04 | -6.0150391 | 0.311438 | -1.51621081 | Prf1 |
| 10525381 | 0.01404 | 4.86E-04 | 6.0148127 | 0.311199 | 1.35802001 | Vps29 |
| 10553057 | 0.01404 | 4.88E-04 | 6.0097196 | 0.305822 | 1.07849244 | Mamstr |
| 10455472 | 0.01404 | 4.89E-04 | 6.0081023 | 0.304113 | 1.03815139 | Dcp2 |
| 10607694 | 0.01404 | 4.89E-04 | 6.0076266 | 0.303611 | 1.16408878 | Syap1 |
| 10353034 | 0.01404 | 4.91E-04 | -6.0030482 | 0.298772 | -1.46104267 | Snord87 |
| 10381708 | 0.01404 | 4.92E-04 | -6.0025179 | 0.298211 | -1.18792596 | Fmnl1 |
| 10565193 | 0.01404 | 4.92E-04 | 6.0020958 | 0.297765 | 1.07588397 | Hdgfrp3 |
| 10417124 | 0.01411 | 4.96E-04 | 5.9933823 | 0.288547 | 1.11568787 | B930095G15Rik |
| 10579012 | 0.01411 | 4.96E-04 | 5.9932146 | 0.28837 | 1.09648212 | Csgalnact1 |
| 10572730 | 0.01411 | 4.97E-04 | 5.991159 | 0.286193 | 1.47932214 | Zfp617 |
| 10536273 | 0.01411 | 4.99E-04 | 5.987783 | 0.282618 | 1.72376648 | Casd1 |
| 10407467 | 0.01411 | 4.99E-04 | 5.9876165 | 0.282442 | 1.22353394 | Akr1e1 |
| 10591228 | 0.01417 | 5.05E-04 | 5.9757179 | 0.269828 | 1.24682502 | Zfp26 |
| 10607774 | 0.01417 | 5.07E-04 | 5.972752 | 0.266681 | 1.12902312 | Mospd2 |
| 10460108 | 0.01423 | 5.10E-04 | 5.9674949 | 0.261099 | 1.20779081 | Gnpnat1 |
| 10585972 | 0.01425 | 5.11E-04 | 5.9655103 | 0.258991 | 1.22292371 | Myo9a |
| 10565486 | 0.01427 | 5.12E-04 | 5.9627461 | 0.256054 | 1.34068596 | Eed |
| 10435031 | 0.01435 | 5.16E-04 | 5.9544838 | 0.247268 | 1.28595914 | Ubxn7 |
| 10434233 | 0.01437 | 5.18E-04 | 5.9506991 | 0.243241 | 1.15854007 | Ufd1l |
| 10398451 | 0.01437 | 5.18E-04 | -5.9506698 | 0.24321 | -2.32221516 | Rps25///Rps25 |
| 10399038 | 0.01442 | 5.21E-04 | 5.9457912 | 0.238015 | 1.14610239 | Zfp386 |
| 10433114 | 0.01445 | 5.23E-04 | 5.9425031 | 0.234512 | 1.23279402 | Itga5 |
| 10353296 | 0.01445 | 5.23E-04 | 5.9423543 | 0.234354 | 1.59289765 | Tceb1 |
| 10586724 | 0.01448 | 5.25E-04 | 5.9381333 | 0.229855 | 1.00992052 | Ice2 |
| 10596273 | 0.01451 | 5.29E-04 | 5.9316214 | 0.222909 | 1.61155919 | Dnajc13 |
| 10535095 | 0.01451 | 5.30E-04 | 5.9299064 | 0.221079 | 1.23361097 | Zfand2a |
| 10556957 | 0.01452 | 5.30E-04 | 5.9283055 | 0.21937 | 1.13127089 | BC030336 |
| 10473045 | 0.01456 | 5.34E-04 | 5.9214656 | 0.212065 | 1.08383082 | Rbm45 |
| 10382516 | 0.01456 | 5.35E-04 | 5.9206732 | 0.211218 | 1.31090033 | Kctd2 |
| 10498210 | 0.01456 | 5.36E-04 | 5.91889 | 0.209313 | 1.01148989 | Nbea |
| 10499130 | 0.01456 | 5.36E-04 | -5.9184759 | 0.20887 | -1.55847765 | Rnu73b |
| 10457331 | 0.01456 | 5.37E-04 | 5.9170203 | 0.207314 | 1.76345624 | Mzt1 |
| 10551423 | 0.01458 | 5.39E-04 | 5.9124993 | 0.202479 | 1.95280297 | Zfp626 |
| 10551426 | 0.01468 | 5.45E-04 | 5.9014004 | 0.190598 | 1.00988831 | Zfp850///Gm10046///Zfp607a///Zfp780b///Zfp607b///Zfp974///Zfp60///Zfp59 |
| 10358635 | 0.01468 | 5.46E-04 | 5.9010888 | 0.190264 | 1.00651446 | Hmcn1 |
| 10529819 | 0.01471 | 5.47E-04 | -5.8982488 | 0.187221 | -1.22598077 | Fgfbp1 |
| 10587012 | 0.01483 | 5.54E-04 | 5.8865291 | 0.174651 | 1.20329721 | Ccpg1 |
| 10525397 | 0.01483 | 5.56E-04 | 5.8833013 | 0.171186 | 1.00772434 | Arpc3 |
| 10605181 | 0.01486 | 5.60E-04 | 5.8751105 | 0.162386 | 1.05405122 | Renbp |
| 10490894 | 0.01496 | 5.67E-04 | 5.8640977 | 0.150539 | 1.27262318 | E2f5 |
| 10443940 | 0.01496 | 5.68E-04 | 5.8615277 | 0.147772 | 1.21751767 | Zfp955b///Zfp955a |
| 10532310 | 0.01496 | 5.69E-04 | 5.8610172 | 0.147222 | 1.80404452 | 4930522L14Rik///Zfp950///A630089N07Rik///Arid4b///Zfp932 |
| 10546586 | 0.015 | 5.71E-04 | 5.8572011 | 0.143111 | 1.00099378 | Tmf1 |
| 10399908 | 0.01501 | 5.72E-04 | 5.8557439 | 0.141541 | 1.01738076 | Prkar2b |
| 10536376 | 0.01511 | 5.77E-04 | 5.8461422 | 0.131187 | 1.12662155 | Mios |
| 10576639 | 0.01513 | 5.78E-04 | 5.8445121 | 0.129428 | 1.05323387 | Nrp1 |
| 10420986 | 0.01514 | 5.80E-04 | 5.8414251 | 0.126095 | 1.983213 | Rpl21 |
| 10346255 | 0.01514 | 5.80E-04 | 5.8411966 | 0.125849 | 1.01484571 | Ormdl1 |
| 10472893 | 0.01514 | 5.80E-04 | 5.8411055 | 0.12575 | 1.15591824 | Zak |
| 10478196 | 0.01514 | 5.81E-04 | 5.8402197 | 0.124794 | 1.25207341 | Top1 |
| 10562578 | 0.01514 | 5.84E-04 | 5.8351972 | 0.119368 | 1.52880517 | Pop4 |
| 10450904 | 0.01514 | 5.84E-04 | 5.8346494 | 0.118776 | 2.17620963 | Scoc |
| 10606376 | 0.01514 | 5.85E-04 | 5.8329881 | 0.116981 | 1.1086478 | 2610002M06Rik |
| 10396177 | 0.01518 | 5.87E-04 | 5.8296905 | 0.113416 | 1.36341865 | Actr10 |
| 10387257 | 0.01519 | 5.90E-04 | 5.8257038 | 0.109104 | 1.01709782 | Alox8 |
| 10547740 | 0.01521 | 5.92E-04 | 5.8222292 | 0.105344 | 1.72585044 | C1s1 |
| 10571399 | 0.01521 | 5.93E-04 | 5.8213098 | 0.104348 | 1.88372056 | Zdhhc2 |
| 10392910 | 0.01521 | 5.93E-04 | 5.8203622 | 0.103322 | 1.06566742 | Hid1 |
| 10491780 | 0.01525 | 5.96E-04 | 5.8164317 | 0.099066 | 1.32851657 | Hspa4l |
| 10346310 | 0.01525 | 5.96E-04 | 5.8160566 | 0.09866 | 1.26394967 | Mob4 |
| 10422728 | 0.01525 | 5.96E-04 | 5.8152532 | 0.097789 | 1.24618011 | Dab2 |
| 10581073 | 0.01525 | 5.97E-04 | 5.8136954 | 0.096101 | 1.01994792 | Dync1li2 |
| 10527441 | 0.01525 | 5.98E-04 | 5.8130064 | 0.095355 | 1.16422729 | Arpc1b |
| 10401781 | 0.01526 | 5.99E-04 | 5.8111714 | 0.093366 | 1.41273582 | Sptlc2 |
| 10501860 | 0.01526 | 5.99E-04 | 5.8111055 | 0.093294 | 1.74365684 | Fnbp1l |
| 10433776 | 0.0153 | 6.02E-04 | 5.8064439 | 0.08824 | 1.23448899 | Snai2 |
| 10581538 | 0.0153 | 6.02E-04 | 5.806075 | 0.08784 | 2.35946557 | Nqo1 |
| 10606735 | 0.0153 | 6.02E-04 | 5.8053401 | 0.087043 | 1.04508186 | Armcx2 |
| 10520815 | 0.01536 | 6.08E-04 | 5.7960245 | 0.076931 | 1.09701015 | Slc4a1ap |
| 10508727 | 0.01536 | 6.09E-04 | 5.7948512 | 0.075657 | 1.33747826 | Dnajc8 |
| 10490931 | 0.01546 | 6.16E-04 | 5.7843757 | 0.064269 | 1.17526261 | Ythdf3 |
| 10597288 | 0.01546 | 6.16E-04 | 5.7839227 | 0.063777 | 1.27346574 | Mlh1 |
| 10498584 | 0.01554 | 6.21E-04 | -5.776896 | 0.056129 | -1.42956129 | Rarres1 |
| 10607302 | 0.01555 | 6.22E-04 | 5.7755201 | 0.054631 | 1.03348629 | Gnl3l |
| 10440471 | 0.01556 | 6.22E-04 | 5.7741783 | 0.05317 | 1.0490939 | Mrpl39 |
| 10405916 | 0.01557 | 6.23E-04 | 5.7731652 | 0.052066 | 1.18264311 | Rslcan18///Zfp738///Zfp65///Zfp729a///Zfp748///Zfp87 |
| 10574151 | 0.01572 | 6.33E-04 | -5.7580509 | 0.035585 | -1.16189792 | Nlrc5 |
| 10431637 | 0.01572 | 6.33E-04 | 5.7577936 | 0.035304 | 1.54452544 | Cpne8 |
| 10405566 | 0.01575 | 6.36E-04 | -5.7539808 | 0.031141 | -1.02978266 | Slc25a48 |
| 10604974 | 0.01582 | 6.41E-04 | 5.7463376 | 0.02279 | 1.56459231 | Cetn2 |
| 10600593 | 0.01582 | 6.41E-04 | 5.7457077 | 0.022101 | 1.68747069 | Gm6793///Hnrnpa3 |
| 10498160 | 0.01582 | 6.41E-04 | 5.7456264 | 0.022013 | 1.79093402 | Ufm1 |
| 10428353 | 0.01583 | 6.42E-04 | 5.7444738 | 0.020752 | 1.14707655 | Lrp12 |
| 10442238 | 0.01591 | 6.47E-04 | 5.7368233 | 0.012383 | 1.73722914 | Gm10509///Zfp983///Zfp51 |
| 10358379 | 0.01591 | 6.48E-04 | 5.7352176 | 0.010625 | 1.08680148 | Trove2 |
| 10492078 | 0.01591 | 6.50E-04 | 5.7325719 | 0.007728 | 1.38392085 | Alg5 |
| 10545458 | 0.01597 | 6.54E-04 | 5.7266455 | 0.001235 | 1.28748978 | Tcf7l1 |
| 10533633 | 0.01597 | 6.54E-04 | 5.7265007 | 0.001077 | 1.00113977 | Diablo |
| 10599346 | 0.01597 | 6.54E-04 | 5.7263177 | 0.000876 | 1.25561193 | LOC100862456///Polr2k |
| 10427293 | 0.01597 | 6.56E-04 | 5.7242871 | -0.00135 | 1.16649074 | Hoxc6 |
| 10473547 | 0.016 | 6.58E-04 | 5.721696 | -0.004191 | 1.27623338 | Srp9 |
| 10344809 | 0.0161 | 6.65E-04 | 5.7116682 | -0.015195 | 1.0892737 | Cspp1 |
| 10504730 | 0.01617 | 6.69E-04 | 5.7051886 | -0.022314 | 1.36790904 | Anp32b |
| 10462231 | 0.01617 | 6.70E-04 | -5.7038762 | -0.023756 | -1.15111459 | Dmrt2 |
| 10574135 | 0.01624 | 6.74E-04 | -5.6988402 | -0.029294 | -1.485399 | Nlrc5 |
| 10365817 | 0.01624 | 6.74E-04 | 5.6978388 | -0.030396 | 1.09375574 | Ntn4 |
| 10537026 | 0.01625 | 6.75E-04 | 5.696907 | -0.031421 | 1.20992155 | Cpa4 |
| 10574133 | 0.01632 | 6.79E-04 | -5.6914003 | -0.037482 | -1.07028672 | Nlrc5 |
| 10502522 | 0.01637 | 6.82E-04 | 5.6867644 | -0.042587 | 1.19409342 | Hs2st1 |
| 10583905 | 0.01637 | 6.82E-04 | 5.6866696 | -0.042692 | 1.62878472 | Sept7 |
| 10400326 | 0.01637 | 6.83E-04 | 5.6860556 | -0.043368 | 1.70487995 | Eapp |
| 10565811 | 0.0164 | 6.86E-04 | -5.6819192 | -0.047927 | -2.14274563 | Snord15b |
| 10516221 | 0.0164 | 6.87E-04 | 5.680906 | -0.049044 | 1.3504709 | D130007C19Rik |
| 10368486 | 0.0164 | 6.87E-04 | 5.6806681 | -0.049306 | 1.05732782 | Rnf146 |
| 10419854 | 0.0164 | 6.88E-04 | 5.6794993 | -0.050595 | 2.30397802 | Slc7a8 |
| 10400668 | 0.01656 | 6.98E-04 | 5.6647333 | -0.066894 | 1.1192586 | Nemf |
| 10373036 | 0.01658 | 7.00E-04 | 5.6628566 | -0.068968 | 1.15076599 | Os9 |
| 10546760 | 0.01661 | 7.02E-04 | 5.6600826 | -0.072034 | 2.12125106 | Ddx3x |
| 10598664 | 0.01665 | 7.06E-04 | 5.654508 | -0.078199 | 1.60653107 | Atp6ap2 |
| 10537882 | 0.01665 | 7.09E-04 | 5.6505022 | -0.082632 | 1.17157359 | Cul1 |
| 10463121 | 0.01665 | 7.09E-04 | 5.6503151 | -0.082839 | 1.05923137 | Zfp518a |
| 10603567 | 0.01665 | 7.10E-04 | 5.6493614 | -0.083895 | 2.22582488 | Dynlt3 |
| 10493995 | 0.01665 | 7.10E-04 | 5.6487448 | -0.084577 | 1.05752613 | S100a10 |
| 10349174 | 0.01666 | 7.11E-04 | 5.6472945 | -0.086183 | 1.00507394 | Serpinb8 |
| 10389484 | 0.01668 | 7.13E-04 | 5.6450339 | -0.088687 | 1.32323332 | Rps6kb1 |
| 10384956 | 0.01674 | 7.17E-04 | 5.6405228 | -0.093685 | 1.83645756 | Chac2 |
| 10581729 | 0.01676 | 7.18E-04 | 5.638264 | -0.096189 | 1.06263966 | Ddx19a |
| 10405280 | 0.01676 | 7.20E-04 | 5.6365855 | -0.09805 | 1.13703288 | Arl10 |
| 10596281 | 0.01676 | 7.20E-04 | 5.6359901 | -0.098711 | 1.13419002 | Dnajc13 |
| 10379989 | 0.01676 | 7.23E-04 | 5.6323179 | -0.102784 | 1.13865357 | Ska2 |
| 10346695 | 0.01676 | 7.23E-04 | 5.6322001 | -0.102915 | 1.18297956 | Nbeal1 |
| 10382049 | 0.01676 | 7.24E-04 | 5.630917 | -0.104339 | 1.17091217 | Ddx42 |
| 10401309 | 0.01681 | 7.28E-04 | 5.6255977 | -0.110244 | 1.26163031 | Synj2bp-cox16///Cox16 |
| 10586064 | 0.01684 | 7.32E-04 | 5.6210821 | -0.11526 | 1.29759742 | Anp32a |
| 10345445 | 0.01684 | 7.35E-04 | 5.6172712 | -0.119495 | 1.07924548 | Arid5a |
| 10480329 | 0.01686 | 7.37E-04 | 5.6135745 | -0.123606 | 1.03583442 | Dnajc1 |
| 10541729 | 0.01705 | 7.48E-04 | -5.6001885 | -0.138508 | -1.14084095 | Cdca3 |
| 10379215 | 0.01705 | 7.48E-04 | 5.5996023 | -0.139161 | 2.07330187 | Ift20 |
| 10437712 | 0.01707 | 7.50E-04 | 5.5978752 | -0.141086 | 1.19197427 | Zc3h7a |
| 10351206 | 0.01708 | 7.51E-04 | 5.5964697 | -0.142652 | 1.0812235 | Selp |
| 10393662 | 0.0171 | 7.53E-04 | 5.5943781 | -0.144984 | 1.10432742 | Nptx1 |
| 10587639 | 0.01718 | 7.57E-04 | 5.5886174 | -0.15141 | 1.06426599 | Nt5e |
| 10563114 | 0.01718 | 7.59E-04 | -5.5870696 | -0.153137 | -1.00112849 | Snord32a///Rpl13a |
| 10545135 | 0.01718 | 7.60E-04 | -5.5855906 | -0.154788 | -1.38325762 | Il12rb2 |
| 10590968 | 0.01718 | 7.60E-04 | 5.5848831 | -0.155578 | 1.04757082 | Ankrd49 |
| 10410650 | 0.01718 | 7.62E-04 | 5.582476 | -0.158266 | 1.71283346 | Zfp825 |
| 10409031 | 0.01718 | 7.62E-04 | 5.5823265 | -0.158433 | 1.38186703 | Dek |
| 10396936 | 0.01718 | 7.62E-04 | 5.5822636 | -0.158503 | 1.89893204 | Smoc1 |
| 10351583 | 0.01718 | 7.63E-04 | 5.5811058 | -0.159796 | 1.1587379 | Pfdn2 |
| 10386394 | 0.01718 | 7.64E-04 | 5.58103 | -0.159881 | 1.09629888 | Zfp867 |
| 10432661 | 0.01718 | 7.64E-04 | -5.5802547 | -0.160747 | -1.11254182 | Galnt6 |
| 10558285 | 0.01718 | 7.64E-04 | 5.5799309 | -0.161108 | 1.35149777 | Zranb1 |
| 10362314 | 0.01718 | 7.66E-04 | 5.5776629 | -0.163642 | 1.02115028 | Ptprk |
| 10608424 | 0.01719 | 7.68E-04 | -5.5760681 | -0.165425 | -2.50967959 | Gm20831///Ssty1 |
| 10411373 | 0.01719 | 7.68E-04 | 5.5751445 | -0.166457 | 1.42058975 | Hexb |
| 10437942 | 0.01719 | 7.69E-04 | 5.5742219 | -0.167488 | 2.3012954 | Ube2v2 |
| 10486552 | 0.0172 | 7.71E-04 | 5.5714769 | -0.170558 | 1.03418339 | Lrrc57 |
| 10388682 | 0.01724 | 7.75E-04 | 5.566763 | -0.175831 | 1.00505722 | Taok1 |
| 10587104 | 0.01724 | 7.75E-04 | 5.5665672 | -0.17605 | 1.50069035 | Arpp19 |
| 10455919 | 0.01726 | 7.77E-04 | 5.5647081 | -0.178131 | 1.0365916 | Adamts19///Adamts19 |
| 10409986 | 0.01726 | 7.78E-04 | 5.5639096 | -0.179025 | 1.32424347 | 4933434E20Rik |
| 10367708 | 0.01731 | 7.85E-04 | 5.5546702 | -0.189374 | 1.29468189 | Pcmt1///Pcmt1 |
| 10358677 | 0.01731 | 7.86E-04 | 5.5539663 | -0.190163 | 1.13882994 | Swt1 |
| 10554325 | 0.01733 | 7.87E-04 | -5.5522779 | -0.192056 | -1.060339 | Ticrr |
| 10381311 | 0.01733 | 7.90E-04 | 5.5496411 | -0.195013 | 1.15207072 | Wnk4 |
| 10409970 | 0.01733 | 7.91E-04 | 5.5478824 | -0.196985 | 1.84880718 | Zfp935 |
| 10476834 | 0.01733 | 7.92E-04 | 5.5471496 | -0.197807 | 1.52612779 | Xrn2 |
| 10415119 | 0.01733 | 7.92E-04 | 5.5464778 | -0.198561 | 1.03813679 | Pabpn1 |
| 10369431 | 0.01734 | 7.95E-04 | -5.5438716 | -0.201486 | -1.28480053 | Adamts14 |
| 10606263 | 0.01734 | 7.95E-04 | 5.5438496 | -0.201511 | 1.28349002 | Atrx |
| 10419223 | 0.01734 | 7.95E-04 | 5.5437092 | -0.201668 | 1.36593878 | Fermt2 |
| 10411839 | 0.01734 | 7.95E-04 | 5.5434073 | -0.202007 | 1.05858469 | Srek1 |
| 10600504 | 0.01736 | 7.98E-04 | 5.5402239 | -0.205581 | 1.43693268 | Fundc2 |
| 10440238 | 0.0174 | 8.00E-04 | 5.5372704 | -0.208898 | 1.51027154 | Nsun3 |
| 10486710 | 0.0174 | 8.01E-04 | 5.5366745 | -0.209568 | 1.22718042 | Lcmt2 |
| 10564159 | 0.01742 | 8.03E-04 | -5.5338893 | -0.212697 | -1.46412405 | Snord116l2///Snord116l1///Snord116 |
| 10523758 | 0.01748 | 8.09E-04 | -5.5273053 | -0.2201 | -1.21581542 | Lrrc8b |
| 10355403 | 0.01755 | 8.13E-04 | 5.5223232 | -0.225705 | 1.17945714 | Fn1 |
| 10481092 | 0.01756 | 8.14E-04 | 5.5211115 | -0.227069 | 1.0244351 | Agpat2 |
| 10357137 | 0.01757 | 8.15E-04 | 5.5197339 | -0.22862 | 1.10703196 | Gli2 |
| 10379677 | 0.01762 | 8.19E-04 | -5.5153393 | -0.23357 | -1.03913832 | Rasl10b |
| 10424413 | 0.01764 | 8.21E-04 | -5.5135316 | -0.235606 | -1.11173901 | Hmgb1-rs17///Gm5176///4932431P20Rik///Hmgb1///Gm12568 |
| 10398455 | 0.01766 | 8.23E-04 | 5.510983 | -0.238479 | 1.54354478 | Ppp2r5c |
| 10600403 | 0.01768 | 8.26E-04 | 5.5073331 | -0.242594 | 1.0408098 | Fam50a |
| 10420348 | 0.01768 | 8.27E-04 | 5.506321 | -0.243735 | 1.43373965 | Zmym5 |
| 10346634 | 0.01768 | 8.28E-04 | 5.5060811 | -0.244006 | 1.10862919 | Nop58 |
| 10442206 | 0.01774 | 8.31E-04 | 5.5018491 | -0.24878 | 1.65846878 | Zfp51 |
| 10519747 | 0.01774 | 8.32E-04 | 5.5011149 | -0.249609 | 1.15364268 | Sema3e |
| 10397818 | 0.01784 | 8.38E-04 | 5.4945788 | -0.256989 | 1.35962711 | Cpsf2 |
| 10410709 | 0.01784 | 8.40E-04 | 5.4925954 | -0.25923 | 1.20608769 | Rfesd |
| 10497501 | 0.01784 | 8.41E-04 | 5.491616 | -0.260336 | 1.20308507 | Naaladl2 |
| 10452867 | 0.01784 | 8.41E-04 | 5.4913652 | -0.26062 | 1.04043586 | Dpy30 |
| 10462136 | 0.01784 | 8.41E-04 | 5.4912672 | -0.260731 | 1.89935995 | Cycs |
| 10467003 | 0.01784 | 8.41E-04 | 5.4911888 | -0.260819 | 2.31108514 | Ppp1r2-ps3///Ppp1r2 |
| 10458428 | 0.01784 | 8.41E-04 | 5.4908615 | -0.261189 | 1.89319091 | Uxt |
| 10353288 | 0.01785 | 8.43E-04 | 5.4885087 | -0.263848 | 1.15708297 | Ube2w |
| 10468653 | 0.01785 | 8.44E-04 | 5.4881989 | -0.264199 | 1.52503098 | Ccdc186 |
| 10508019 | 0.01792 | 8.48E-04 | 5.4836066 | -0.269392 | 1.35344873 | Gnl2 |
| 10487643 | 0.01798 | 8.52E-04 | -5.4790679 | -0.274528 | -1.10473496 | Rpl27a |
| 10585444 | 0.01798 | 8.53E-04 | 5.4776738 | -0.276106 | 1.04336219 | Ireb2 |
| 10522396 | 0.01798 | 8.54E-04 | 5.4767103 | -0.277197 | 1.27848111 | Ociad1 |
| 10434942 | 0.01798 | 8.55E-04 | 5.476229 | -0.277741 | 1.19659532 | Dlg1 |
| 10484463 | 0.01798 | 8.56E-04 | 5.474516 | -0.279681 | 2.10009843 | Serping1 |
| 10529260 | 0.01798 | 8.57E-04 | 5.4734761 | -0.280859 | 1.27142932 | Prr14l |
| 10420899 | 0.01798 | 8.58E-04 | 5.4732455 | -0.28112 | 1.00833296 | Gulo |
| 10527528 | 0.01798 | 8.58E-04 | 5.4731372 | -0.281243 | 2.25092255 | Smarce1 |
| 10587746 | 0.01798 | 8.58E-04 | 5.4728869 | -0.281526 | 1.58477137 | Tmem41b |
| 10534883 | 0.01801 | 8.60E-04 | -5.4710474 | -0.283611 | -1.04136998 | Irs3 |
| 10479077 | 0.01801 | 8.60E-04 | 5.4704959 | -0.284235 | 1.01509497 | Vapb |
| 10375820 | 0.01803 | 8.62E-04 | 5.4681212 | -0.286927 | 1.1453367 | Clk4 |
| 10538979 | 0.01807 | 8.67E-04 | -5.4633533 | -0.292333 | -1.36198324 | Cd8b1 |
| 10587655 | 0.01816 | 8.72E-04 | 5.457504 | -0.298969 | 1.17757037 | Zfp949 |
| 10599120 | 0.01818 | 8.75E-04 | 5.454607 | -0.302258 | 1.07553129 | Dock11 |
| 10590597 | 0.01818 | 8.76E-04 | 5.4533262 | -0.303713 | 1.10197438 | Sacm1l |
| 10399588 | 0.01818 | 8.77E-04 | 5.4530015 | -0.304081 | 1.64051432 | Zfp125 |
| 10456709 | 0.01822 | 8.80E-04 | 5.4490215 | -0.308603 | 1.15806171 | Gm6133///Rpl17///Hk1 |
| 10563829 | 0.01826 | 8.87E-04 | 5.4417721 | -0.316844 | 1.18240962 | Mrps33 |
| 10434224 | 0.01829 | 8.90E-04 | 5.439472 | -0.319461 | 1.15490928 | Gnb1l |
| 10593492 | 0.01833 | 8.93E-04 | 5.4360606 | -0.323343 | 1.10998669 | Zc3h12c |
| 10585048 | 0.0184 | 8.97E-04 | 5.4313957 | -0.328654 | 1.26057491 | Cadm1 |
| 10440288 | 0.01849 | 9.05E-04 | 5.4236853 | -0.33744 | 1.31698337 | Zfp654 |
| 10574161 | 0.01849 | 9.06E-04 | -5.4225132 | -0.338776 | -1.115708 | Nlrc5 |
| 10411552 | 0.01852 | 9.10E-04 | 5.4191039 | -0.342664 | 1.30911425 | Bdp1 |
| 10501208 | 0.01853 | 9.10E-04 | 5.4183177 | -0.343561 | 1.3884932 | Gstm6 |
| 10602977 | 0.01853 | 9.11E-04 | 5.4177694 | -0.344186 | 1.32348188 | Scml2 |
| 10585249 | 0.01856 | 9.14E-04 | 5.4150068 | -0.347339 | 1.07050173 | Ppp2r1b |
| 10488147 | 0.01857 | 9.15E-04 | 5.4138769 | -0.348628 | 1.14955474 | Flrt3 |
| 10583326 | 0.01858 | 9.17E-04 | 5.4119972 | -0.350774 | 1.35576967 | Slc36a4 |
| 10541094 | 0.0186 | 9.21E-04 | 5.4075762 | -0.355824 | 1.30496341 | Zfp637 |
| 10482139 | 0.0186 | 9.22E-04 | 5.4071589 | -0.3563 | 1.32090428 | Pdcl |
| 10502823 | 0.01871 | 9.31E-04 | 5.3976676 | -0.367151 | 1.06450792 | Dnajb4 |
| 10363599 | 0.01874 | 9.35E-04 | 5.3942757 | -0.371031 | 1.05070072 | Rufy2 |
| 10360983 | 0.01874 | 9.36E-04 | 5.3929613 | -0.372536 | 1.46122538 | Gm3837///Pdcd5 |
| 10596148 | 0.01874 | 9.36E-04 | 5.392544 | -0.373013 | 1.5021381 | Trf |
| 10463140 | 0.01874 | 9.38E-04 | 5.3911409 | -0.37462 | 1.01927838 | Lcor |
| 10344990 | 0.01877 | 9.44E-04 | 5.3852449 | -0.381372 | 1.0303507 | Crispld1 |
| 10522250 | 0.01877 | 9.46E-04 | 5.3830155 | -0.383927 | 1.03477379 | Tmem33 |
| 10439790 | 0.01877 | 9.50E-04 | -5.3793092 | -0.388176 | -1.00573101 | Trat1 |
| 10595288 | 0.01877 | 9.50E-04 | 5.3789237 | -0.388618 | 1.25303576 | Tmem30a |
| 10579296 | 0.01877 | 9.51E-04 | 5.3787776 | -0.388785 | 1.1187777 | 2810428I15Rik |
| 10422512 | 0.01878 | 9.51E-04 | 5.3782021 | -0.389445 | 1.14134582 | Ggact |
| 10424213 | 0.01882 | 9.56E-04 | 5.3738974 | -0.394383 | 1.04019768 | Zhx2 |
| 10411059 | 0.01892 | 9.65E-04 | 5.3650197 | -0.404575 | 1.03332241 | Zfyve16 |
| 10394699 | 0.01892 | 9.65E-04 | 5.3648848 | -0.40473 | 1.52392652 | Rock2 |
| 10405994 | 0.01892 | 9.65E-04 | 5.3647586 | -0.404875 | 1.54382887 | Med10 |
| 10463070 | 0.01892 | 9.66E-04 | 5.3644947 | -0.405178 | 1.02900949 | Entpd1 |
| 10492220 | 0.01894 | 9.67E-04 | 5.3630759 | -0.406808 | 1.57606491 | Selt |
| 10600911 | 0.01899 | 9.71E-04 | 5.3598687 | -0.410494 | 1.31787708 | Yipf6 |
| 10467921 | 0.01899 | 9.72E-04 | 5.3583748 | -0.412212 | 1.25888347 | Chuk |
| 10558118 | 0.01899 | 9.73E-04 | -5.3576867 | -0.413003 | -1.11810137 | Btbd16 |
| 10516435 | 0.01899 | 9.74E-04 | 5.3569083 | -0.413898 | 1.11568634 | Zmym4 |
| 10586880 | 0.01899 | 9.74E-04 | 5.3563069 | -0.414589 | 1.47410862 | Zfp280d |
| 10494016 | 0.01902 | 9.78E-04 | 5.3530841 | -0.418297 | 1.95793975 | Them5 |
| 10469720 | 0.01902 | 9.81E-04 | 5.350342 | -0.421452 | 1.0648026 | Acbd5 |
| 10394532 | 0.01902 | 9.82E-04 | 5.3494033 | -0.422532 | 1.22328029 | Gm5434///Ube2f |
| 10495659 | 0.0191 | 9.91E-04 | 5.3413397 | -0.431819 | 1.17847611 | Cnn3 |
| 10507914 | 0.01911 | 9.93E-04 | 5.3389753 | -0.434544 | 1.42373367 | Sf3a3 |
| 10377255 | 0.01916 | 9.97E-04 | 5.3354892 | -0.438563 | 1.11393604 | Stx8 |
| 10446928 | 0.0192 | 1.00E-03 | 5.3296871 | -0.445256 | 1.03598752 | Ltbp1 |
| 10570771 | 0.0192 | 1.00E-03 | 5.3290507 | -0.44599 | 1.40979921 | Vps36 |
| 10585533 | 0.01925 | 1.01E-03 | 5.3220804 | -0.454038 | 1.92255053 | LOC102633596///Dnajb6 |
| 10361918 | 0.01929 | 1.02E-03 | -5.3175145 | -0.459314 | -1.2428368 | Il20ra |
| 10425207 | 0.01931 | 1.02E-03 | 5.3154046 | -0.461753 | 1.34407886 | H1f0 |
| 10589955 | 0.01931 | 1.02E-03 | -5.3141553 | -0.463197 | -1.1109818 | Gm9757 |
| 10503551 | 0.01931 | 1.02E-03 | 5.3134584 | -0.464003 | 1.32230379 | Usp45 |
| 10604248 | 0.01934 | 1.03E-03 | 5.3100679 | -0.467925 | 1.48962714 | Thoc2 |
| 10503570 | 0.01934 | 1.03E-03 | 5.3097399 | -0.468304 | 1.06094877 | Pnisr |
| 10439471 | 0.01934 | 1.03E-03 | 5.3055302 | -0.473176 | 1.09936122 | Poglut1 |
| 10578922 | 0.01934 | 1.03E-03 | 5.3036726 | -0.475327 | 1.15749503 | Klhl2 |
| 10551287 | 0.01935 | 1.03E-03 | -5.3024172 | -0.47678 | -1.02974273 | Cyp2a12 |
| 10491261 | 0.01935 | 1.03E-03 | 5.3018119 | -0.477481 | 1.15326293 | Sec62 |
| 10502191 | 0.01939 | 1.04E-03 | 5.2995089 | -0.480149 | 1.1651402 | Ostc |
| 10592585 | 0.01939 | 1.04E-03 | 5.2982617 | -0.481594 | 1.02451605 | Sc5d |
| 10515744 | 0.0194 | 1.04E-03 | -5.2958394 | -0.484401 | -1.0449676 | Cdc20 |
| 10572906 | 0.01942 | 1.04E-03 | -5.2936959 | -0.486886 | -1.10883566 | Mcm5 |
| 10352348 | 0.01944 | 1.05E-03 | 5.2907262 | -0.49033 | 1.67475643 | Cnih4 |
| 10593605 | 0.01944 | 1.05E-03 | 5.2903873 | -0.490723 | 1.05170391 | Cul5 |
| 10390175 | 0.01944 | 1.05E-03 | -5.2901906 | -0.490951 | -1.02700188 | Ngfr |
| 10455238 | 0.01944 | 1.05E-03 | 5.2893982 | -0.49187 | 1.2618207 | Ndfip1 |
| 10536818 | 0.01949 | 1.05E-03 | 5.2866439 | -0.495066 | 1.62585809 | Calu |
| 10496735 | 0.01956 | 1.06E-03 | 5.2795593 | -0.50329 | 1.00652136 | Gm6525///Rpl36a |
| 10462507 | 0.01958 | 1.06E-03 | 5.2758596 | -0.507589 | 1.10856051 | Papss2 |
| 10571288 | 0.01958 | 1.07E-03 | 5.2729588 | -0.51096 | 1.2747321 | Gtf2e2 |
| 10428714 | 0.01963 | 1.07E-03 | 5.2674895 | -0.51732 | 1.10823447 | Slc22a22 |
| 10419170 | 0.01972 | 1.09E-03 | 5.2578718 | -0.528515 | 1.74974639 | Txndc16 |
| 10431612 | 0.01972 | 1.09E-03 | 5.2570566 | -0.529465 | 1.43648473 | Rabl2 |
| 10553568 | 0.01987 | 1.11E-03 | 5.24261 | -0.546309 | 1.4697775 | Tubgcp5 |
| 10372457 | 0.01994 | 1.11E-03 | 5.2346213 | -0.555637 | 1.27613539 | Tbc1d15 |
| 10491182 | 0.01994 | 1.12E-03 | 5.2335249 | -0.556918 | 1.01442672 | Eif5a2 |
| 10561837 | 0.02005 | 1.12E-03 | 5.2269169 | -0.564642 | 1.08199185 | Zfp146 |
| 10482181 | 0.02007 | 1.13E-03 | 5.224837 | -0.567074 | 1.19365179 | Strbp |
| 10371275 | 0.0201 | 1.13E-03 | -5.2227473 | -0.569519 | -1.12711427 | Rpl23a |
| 10499996 | 0.02011 | 1.13E-03 | 5.2212868 | -0.571228 | 1.25278871 | Snx27 |
| 10447490 | 0.02017 | 1.14E-03 | 5.2179126 | -0.575177 | 1.07750717 | Pja2 |
| 10540544 | 0.02025 | 1.14E-03 | 5.2120926 | -0.581994 | 1.42049001 | Thumpd3 |
| 10417689 | 0.02032 | 1.15E-03 | 5.2069491 | -0.588022 | 1.04236016 | Psmd6 |
| 10542750 | 0.02034 | 1.15E-03 | 5.2046638 | -0.590702 | 1.36173495 | Med21 |
| 10346808 | 0.02037 | 1.15E-03 | 5.2031053 | -0.59253 | 1.03670427 | Rpl17///Hk1 |
| 10586246 | 0.02037 | 1.16E-03 | 5.2026002 | -0.593122 | 1.03056772 | Dennd4a |
| 10354286 | 0.02037 | 1.16E-03 | 5.2023242 | -0.593446 | 1.01104572 | Kdelc1 |
| 10410264 | 0.02038 | 1.16E-03 | 5.1999827 | -0.596193 | 1.41248042 | Mterf3 |
| 10345141 | 0.02038 | 1.16E-03 | 5.1999083 | -0.59628 | 1.11615323 | Lmbrd1 |
| 10408037 | 0.02046 | 1.17E-03 | -5.1939568 | -0.603267 | -1.03985592 | Nkapl |
| 10523905 | 0.02046 | 1.17E-03 | 5.1930813 | -0.604295 | 1.14490191 | Mtf2 |
| 10598107 | 0.02046 | 1.17E-03 | 5.1921809 | -0.605353 | 1.55696162 | Taf1a |
| 10490273 | 0.02056 | 1.18E-03 | 5.1855388 | -0.613158 | 1.61037464 | Gm4724///Gm14410///Gm14305///Gm14308///Gm14295///0610010B08Rik///Gm14434///Gm14432///Gm14391///Gm14430///Gm6710 |
| 10380887 | 0.02061 | 1.18E-03 | -5.182674 | -0.616527 | -1.13232988 | Tcap |
| 10491455 | 0.02063 | 1.19E-03 | 5.1800629 | -0.619598 | 1.55599641 | Fxr1 |
| 10552264 | 0.02066 | 1.19E-03 | 5.1785443 | -0.621385 | 1.12949374 | Zfp939 |
| 10419038 | 0.02066 | 1.19E-03 | 5.1741834 | -0.626517 | 1.13581231 | Ghitm |
| 10597266 | 0.02068 | 1.20E-03 | -5.1721734 | -0.628884 | -1.02959171 | Als2cl |
| 10368041 | 0.02069 | 1.20E-03 | 5.1695203 | -0.632009 | 1.01145971 | Abracl///Abracl |
| 10345666 | 0.02069 | 1.20E-03 | 5.1684102 | -0.633317 | 1.10193707 | Pdcl3 |
| 10410877 | 0.02069 | 1.20E-03 | 5.1677633 | -0.634079 | 1.05208248 | Polr3g |
| 10526866 | 0.0207 | 1.20E-03 | -5.1657189 | -0.636489 | -1.0661214 | Gm5294 |
| 10354732 | 0.0207 | 1.20E-03 | 5.1656826 | -0.636531 | 1.83461104 | Hspd1 |
| 10506743 | 0.02082 | 1.22E-03 | 5.1558621 | -0.648113 | 1.37779797 | 0610037L13Rik |
| 10458293 | 0.02082 | 1.22E-03 | 5.1528372 | -0.651684 | 1.00820621 | Dnajc18 |
| 10358603 | 0.02082 | 1.22E-03 | 5.1516891 | -0.653039 | 1.42471296 | Hmcn1 |
| 10409990 | 0.02082 | 1.23E-03 | 5.1486053 | -0.656681 | 1.63003626 | 6720489N17Rik |
| 10412207 | 0.02082 | 1.23E-03 | 5.1482251 | -0.65713 | 1.74555069 | Gpx8 |
| 10360695 | 0.02082 | 1.23E-03 | 5.1473148 | -0.658206 | 1.28576983 | Nvl |
| 10399224 | 0.02082 | 1.23E-03 | 5.1470162 | -0.658559 | 1.15953083 | 1110002L01Rik |
| 10400350 | 0.02083 | 1.23E-03 | 5.1460132 | -0.659744 | 1.21832246 | Cfl2 |
| 10490294 | 0.0209 | 1.24E-03 | 5.139986 | -0.666868 | 1.24002364 | Gm4724///Gm14410///Gm14305///Gm14308///0610010B08Rik///Gm14434///Gm14432///Gm14326///Gm14391///Gm14393///Zfp970///Gm14430///Zfp971///Gm6710///Gm14322///Gm14403///Zfp931///Gm14325///Gm14440///Gm14327 |
| 10413813 | 0.02094 | 1.25E-03 | 5.1361341 | -0.671424 | 1.0659906 | Galnt15 |
| 10385842 | 0.02094 | 1.25E-03 | 5.1356767 | -0.671965 | 1.29426657 | Rad50 |
| 10411359 | 0.02094 | 1.25E-03 | -5.1352584 | -0.67246 | -1.02479692 | Plp2 |
| 10468531 | 0.02094 | 1.25E-03 | 5.1349925 | -0.672775 | 1.58859201 | Nutf2-ps1///Nutf2 |
| 10543213 | 0.02094 | 1.25E-03 | 5.1347038 | -0.673117 | 1.32593938 | Bmt2 |
| 10442219 | 0.02094 | 1.25E-03 | 5.1341968 | -0.673717 | 1.09580988 | Zfp52 |
| 10472289 | 0.02096 | 1.25E-03 | 5.1329519 | -0.67519 | 1.57222558 | Tank |
| 10606056 | 0.02096 | 1.25E-03 | 5.1325128 | -0.67571 | 1.20127412 | Vma21 |
| 10492180 | 0.02096 | 1.25E-03 | 5.131086 | -0.677399 | 1.57046516 | Rnf13 |
| 10536595 | 0.02096 | 1.25E-03 | 5.1306197 | -0.677951 | 1.99429213 | Lsm8 |
| 10439762 | 0.02098 | 1.26E-03 | 5.1288753 | -0.680016 | 2.28896613 | Ahcy///Gm4737 |
| 10467344 | 0.021 | 1.26E-03 | 5.1258383 | -0.683613 | 1.03919903 | Noc3l |
| 10608138 | 0.021 | 1.26E-03 | 5.1253729 | -0.684165 | 1.04142694 | Ddx3y |
| 10537184 | 0.02108 | 1.27E-03 | 5.1206949 | -0.689709 | 1.07215844 | Cald1 |
| 10420362 | 0.0211 | 1.27E-03 | 5.1187275 | -0.692041 | 1.53293817 | Gjb2 |
| 10344817 | 0.0211 | 1.27E-03 | 5.1186799 | -0.692097 | 1.55353669 | Cspp1 |
| 10450206 | 0.02113 | 1.28E-03 | 5.1152714 | -0.69614 | 1.0453561 | Rnf5 |
| 10401335 | 0.02116 | 1.28E-03 | 5.1127288 | -0.699157 | 1.78533339 | Med6 |
| 10399540 | 0.02124 | 1.29E-03 | 5.1070189 | -0.705935 | 1.18881078 | Pqlc3 |
| 10404063 | 0.02129 | 1.29E-03 | -5.1024594 | -0.711351 | -1.11206179 | Hist1h2ab |
| 10608422 | 0.02129 | 1.29E-03 | 5.101705 | -0.712248 | 1.71075839 | Rmi1 |
| 10607679 | 0.02134 | 1.30E-03 | 5.0981944 | -0.71642 | 1.55891976 | Txlng |
| 10539769 | 0.02138 | 1.31E-03 | 5.0935139 | -0.721986 | 1.32569481 | Nfu1 |
| 10436304 | 0.02138 | 1.31E-03 | 5.0900252 | -0.726137 | 1.38382604 | Abi3bp |
| 10394770 | 0.02138 | 1.31E-03 | 5.0898676 | -0.726325 | 1.0951602 | Odc1 |
| 10594277 | 0.02154 | 1.33E-03 | -5.079529 | -0.738637 | -1.15971188 | Paqr5 |
| 10440050 | 0.02158 | 1.34E-03 | 5.0743221 | -0.744844 | 1.13909042 | Tbc1d23 |
| 10394990 | 0.02161 | 1.34E-03 | 5.0718459 | -0.747798 | 1.40966274 | Mboat2 |
| 10468929 | 0.02174 | 1.35E-03 | 5.0627149 | -0.758696 | 1.07574859 | Nmt2 |
| 10596263 | 0.02174 | 1.36E-03 | 5.0613088 | -0.760376 | 1.11341554 | Dnajc13 |
| 10490872 | 0.02191 | 1.37E-03 | 5.0531585 | -0.770116 | 1.07175604 | Lrrcc1 |
| 10468253 | 0.02195 | 1.37E-03 | 5.0503156 | -0.773516 | 1.12355306 | Nt5c2 |
| 10350684 | 0.02196 | 1.37E-03 | 5.049105 | -0.774964 | 1.25234642 | Arpc5///Arpc5 |
| 10491922 | 0.02196 | 1.38E-03 | 5.0472958 | -0.777129 | 1.16223891 | Naa15 |
| 10525877 | 0.02199 | 1.38E-03 | 5.0438256 | -0.781283 | 1.47205351 | Zfp664 |
| 10450055 | 0.02199 | 1.38E-03 | 5.0434256 | -0.781761 | 1.08216235 | Pfdn6 |
| 10400095 | 0.02203 | 1.39E-03 | 5.0404134 | -0.785368 | 1.35756189 | Ifrd1 |
| 10563641 | 0.0221 | 1.39E-03 | 5.0367418 | -0.789767 | 2.01786299 | Gm9392///Rap1a |
| 10498168 | 0.02212 | 1.40E-03 | 5.0353177 | -0.791473 | 1.46882134 | Exosc8 |
| 10419132 | 0.02213 | 1.40E-03 | -5.0343633 | -0.792617 | -2.03741969 | Gm17026///Gm10375///Gm10377///BC061237///1700001F09Rik |
| 10586477 | 0.0222 | 1.41E-03 | 5.0295779 | -0.798355 | 1.52941978 | Ppib |
| 10595573 | 0.02222 | 1.41E-03 | 5.0264304 | -0.80213 | 1.00012979 | Snx14 |
| 10371830 | 0.02222 | 1.41E-03 | 5.0259646 | -0.802689 | 1.34616475 | Actr6 |
| 10367770 | 0.02222 | 1.41E-03 | 5.0251991 | -0.803608 | 1.0024281 | Gm9930 |
| 10486057 | 0.02222 | 1.41E-03 | 5.0251521 | -0.803664 | 1.20197787 | Rab5b |
| 10491605 | 0.02225 | 1.42E-03 | 5.0222691 | -0.807124 | 1.25361894 | 4932438A13Rik |
| 10577838 | 0.02225 | 1.42E-03 | 5.0217728 | -0.80772 | 1.0874722 | Ddhd2 |
| 10545583 | 0.02225 | 1.42E-03 | 5.0209561 | -0.808701 | 1.62246103 | Pole4 |
| 10464415 | 0.02225 | 1.42E-03 | 5.0183373 | -0.811845 | 1.13060899 | Fam45a |
| 10394800 | 0.02225 | 1.43E-03 | 5.0177311 | -0.812573 | 1.2409156 | Bnip3l |
| 10585301 | 0.02228 | 1.43E-03 | 5.013186 | -0.818034 | 1.1184903 | Rdx |
| 10585988 | 0.02232 | 1.44E-03 | 5.0089406 | -0.823138 | 1.60483556 | Myo9a |
| 10538755 | 0.02232 | 1.44E-03 | 5.0086152 | -0.823529 | 1.19720209 | Smarcad1 |
| 10372726 | 0.02232 | 1.44E-03 | -5.0079659 | -0.82431 | -1.3482575 | Cdc5l |
| 10593508 | 0.02232 | 1.44E-03 | 5.0074736 | -0.824902 | 1.11113228 | Ddx10 |
| 10544570 | 0.0224 | 1.45E-03 | 5.0022516 | -0.831185 | 2.11643563 | Pcnp |
| 10432180 | 0.0224 | 1.45E-03 | 5.0022207 | -0.831222 | 1.18199368 | Ccnt1 |
| 10350331 | 0.02241 | 1.45E-03 | 5.0015567 | -0.832021 | 1.14269968 | Zfp281 |
| 10440564 | 0.02242 | 1.45E-03 | 4.9997271 | -0.834223 | 1.42717364 | Ltn1 |
| 10388734 | 0.02255 | 1.47E-03 | 4.9922505 | -0.843229 | 1.01890772 | Eral1 |
| 10502638 | 0.02263 | 1.47E-03 | -4.988146 | -0.848176 | -1.0835863 | Clca2 |
| 10397189 | 0.02267 | 1.48E-03 | 4.9844259 | -0.852662 | 1.27992699 | Ptgr2 |
| 10449775 | 0.02271 | 1.49E-03 | -4.9814784 | -0.856218 | -1.05108908 | Notch3 |
| 10451213 | 0.02271 | 1.49E-03 | 4.981071 | -0.85671 | 1.1267185 | Rsph9 |
| 10534426 | 0.02274 | 1.49E-03 | 4.9785172 | -0.859792 | 1.31219538 | Fzd9 |
| 10405074 | 0.02274 | 1.49E-03 | 4.9781091 | -0.860285 | 1.04053664 | Nol8 |
| 10549256 | 0.02276 | 1.50E-03 | 4.974343 | -0.864832 | 2.15716339 | Kras |
| 10354372 | 0.02276 | 1.50E-03 | -4.973822 | -0.865462 | -1.67072741 | Myl6///Myl6 |
| 10596271 | 0.02276 | 1.50E-03 | 4.9702276 | -0.869804 | 1.01801182 | Dnajc13 |
| 10433228 | 0.02278 | 1.51E-03 | 4.9683973 | -0.872016 | 1.05367761 | Cluap1 |
| 10358627 | 0.02287 | 1.52E-03 | 4.9618061 | -0.879986 | 1.8718217 | Hmcn1 |
| 10536429 | 0.02287 | 1.52E-03 | 4.9612699 | -0.880635 | 1.17446565 | Tmem106b |
| 10384448 | 0.02287 | 1.52E-03 | 4.9588196 | -0.8836 | 1.92984163 | LOC102637269///Gm4184///Sec61g |
| 10568464 | 0.02287 | 1.53E-03 | 4.9575477 | -0.885139 | 1.06476712 | Ate1 |
| 10536407 | 0.02287 | 1.53E-03 | 4.9574538 | -0.885253 | 1.13470656 | Phf14 |
| 10440284 | 0.02287 | 1.53E-03 | 4.9573286 | -0.885404 | 1.85597731 | 4930453N24Rik |
| 10498309 | 0.02287 | 1.53E-03 | 4.9569934 | -0.88581 | 1.14857875 | Pfn2 |
| 10518805 | 0.0229 | 1.53E-03 | 4.9544673 | -0.888868 | 1.24104454 | Vamp3 |
| 10407390 | 0.0229 | 1.53E-03 | 4.9535546 | -0.889973 | 1.01581809 | Ptbp1 |
| 10519945 | 0.0229 | 1.53E-03 | 4.9532529 | -0.890339 | 1.13723528 | Tmem60 |
| 10597973 | 0.023 | 1.54E-03 | 4.9479588 | -0.896752 | 1.74475006 | Lztfl1 |
| 10497490 | 0.02301 | 1.55E-03 | 4.9470202 | -0.897889 | 1.52707417 | Naaladl2 |
| 10595604 | 0.02305 | 1.55E-03 | 4.9448238 | -0.900552 | 1.06912214 | Syncrip |
| 10416155 | 0.02305 | 1.55E-03 | 4.9435032 | -0.902153 | 1.26030924 | Kctd9 |
| 10388377 | 0.02305 | 1.55E-03 | 4.9427505 | -0.903066 | 1.21266105 | Srr |
| 10460259 | 0.02305 | 1.55E-03 | -4.9425317 | -0.903331 | -1.0718637 | Aldh3b2 |
| 10344750 | 0.02316 | 1.57E-03 | 4.9354677 | -0.911901 | 1.02991904 | Sgk3 |
| 10440246 | 0.02317 | 1.57E-03 | 4.934794 | -0.912719 | 1.01055618 | Arl13b |
| 10408204 | 0.02318 | 1.57E-03 | -4.9324567 | -0.915556 | -1.0146181 | Hist1h2ae |
| 10606513 | 0.02318 | 1.57E-03 | 4.9324526 | -0.915561 | 1.05375909 | Chm |
| 10446693 | 0.02321 | 1.58E-03 | 4.9303527 | -0.918111 | 1.21679141 | Wdr43 |
| 10446207 | 0.02335 | 1.59E-03 | 4.9227025 | -0.927407 | 1.23173946 | Clpp |
| 10475912 | 0.02335 | 1.59E-03 | 4.9223614 | -0.927822 | 1.37733775 | Tmem87b |
| 10409684 | 0.02338 | 1.59E-03 | 4.920154 | -0.930506 | 1.16486725 | 2210016F16Rik |
| 10591241 | 0.0234 | 1.60E-03 | 4.9187024 | -0.932271 | 1.02216144 | Zfp426 |
| 10439651 | 0.02341 | 1.60E-03 | 4.9174154 | -0.933837 | 1.07504429 | Cd200 |
| 10465895 | 0.02349 | 1.61E-03 | 4.9114034 | -0.941153 | 1.03036231 | Fads2 |
| 10552276 | 0.02349 | 1.61E-03 | 4.9111419 | -0.941472 | 1.16188461 | Ube2h |
| 10479268 | 0.02349 | 1.61E-03 | 4.9106576 | -0.942061 | 1.26121039 | Wtap |
| 10527598 | 0.02351 | 1.62E-03 | 4.9083646 | -0.944853 | 1.29836621 | Pomp |
| 10592856 | 0.02351 | 1.62E-03 | 4.9078408 | -0.945491 | 1.24349182 | Ccdc84 |
| 10526754 | 0.02352 | 1.62E-03 | 4.9069494 | -0.946577 | 1.14850727 | Ap4m1 |
| 10499168 | 0.02354 | 1.62E-03 | 4.9052632 | -0.948631 | 1.07224328 | Kirrel |
| 10607497 | 0.02357 | 1.63E-03 | 4.9027996 | -0.951633 | 1.2811889 | Suclg2 |
| 10586616 | 0.02366 | 1.64E-03 | 4.8961049 | -0.959796 | 1.01415541 | Vps13c |
| 10473384 | 0.02376 | 1.66E-03 | 4.8880227 | -0.96966 | 1.11319965 | Slc43a3 |
| 10362394 | 0.02376 | 1.66E-03 | 4.8875808 | -0.970199 | 1.24975743 | Hddc2 |
| 10423068 | 0.02376 | 1.66E-03 | 4.8875386 | -0.970251 | 1.23605867 | Rad1 |
| 10372917 | 0.02377 | 1.66E-03 | 4.8865153 | -0.971501 | 1.12464485 | Tmem5 |
| 10523206 | 0.02389 | 1.67E-03 | 4.8814892 | -0.977641 | 1.20868184 | Uso1 |
| 10514173 | 0.02393 | 1.68E-03 | 4.8760314 | -0.984313 | 2.86660357 | Gm4705///Rpl34-ps1///Rpl34 |
| 10574718 | 0.02407 | 1.69E-03 | 4.869126 | -0.992761 | 1.08908236 | Tmem208 |
| 10583402 | 0.02407 | 1.69E-03 | 4.8684435 | -0.993596 | 1.24696804 | Zfp317 |
| 10366238 | 0.02407 | 1.69E-03 | 4.8683183 | -0.993749 | 1.23754078 | Ppp1r12a |
| 10410355 | 0.02408 | 1.70E-03 | 4.86769 | -0.994518 | 1.25219171 | Zfp729a |
| 10485445 | 0.02424 | 1.72E-03 | -4.8570807 | -1.007515 | -1.12046976 | Ehf |
| 10596277 | 0.02427 | 1.72E-03 | 4.854532 | -1.010639 | 1.25300415 | Dnajc13 |
| 10538282 | 0.02427 | 1.72E-03 | 4.8527709 | -1.012799 | 1.2618023 | Cbx3 |
| 10476395 | 0.02427 | 1.73E-03 | 4.8525639 | -1.013053 | 1.09625813 | Bmp2 |
| 10553336 | 0.02427 | 1.73E-03 | 4.8524787 | -1.013157 | 1.04484178 | Zdhhc13 |
| 10571384 | 0.02427 | 1.73E-03 | 4.8511865 | -1.014742 | 1.01715385 | Micu3 |
| 10397179 | 0.02435 | 1.74E-03 | 4.8454594 | -1.021771 | 1.25016218 | Dnal1 |
| 10388337 | 0.02439 | 1.75E-03 | 4.8426217 | -1.025255 | 1.2195385 | Pafah1b1 |
| 10467907 | 0.02439 | 1.75E-03 | 4.8415078 | -1.026623 | 1.4248943 | Erlin1 |
| 10515012 | 0.02439 | 1.75E-03 | 4.8414462 | -1.026698 | 1.19446109 | Prpf38a |
| 10463263 | 0.02439 | 1.75E-03 | 4.8394111 | -1.029198 | 2.40297082 | Lztfl1 |
| 10586816 | 0.02439 | 1.75E-03 | 4.8385259 | -1.030286 | 1.01116388 | Sltm |
| 10584758 | 0.02439 | 1.75E-03 | 4.8383872 | -1.030456 | 1.11367255 | Gm10767///Fam103a1///Fam103a1 |
| 10561516 | 0.02443 | 1.76E-03 | 4.8346113 | -1.035097 | 1.16163435 | Nfkbib |
| 10501778 | 0.02443 | 1.77E-03 | 4.8324818 | -1.037715 | 1.1111555 | Ptbp2 |
| 10381304 | 0.02443 | 1.77E-03 | 4.8318706 | -1.038466 | 1.09230589 | Vps25 |
| 10365302 | 0.02448 | 1.77E-03 | 4.8285772 | -1.042517 | 1.54372829 | A230046K03Rik |
| 10504008 | 0.02462 | 1.79E-03 | 4.8225671 | -1.049913 | 1.80178547 | Chmp5 |
| 10467368 | 0.02464 | 1.79E-03 | 4.8191605 | -1.054108 | 1.22445444 | Ctdspl2 |
| 10488029 | 0.02469 | 1.80E-03 | -4.8164758 | -1.057415 | -1.77092021 | Zfand1 |
| 10418720 | 0.02469 | 1.80E-03 | 4.8149686 | -1.059272 | 1.22603072 | Mettl6 |
| 10518774 | 0.02469 | 1.80E-03 | 4.8148715 | -1.059392 | 1.35441314 | Park7 |
| 10496629 | 0.02469 | 1.80E-03 | 4.8145598 | -1.059776 | 1.14809411 | Sep15 |
| 10601760 | 0.02469 | 1.80E-03 | 4.8140395 | -1.060417 | 1.18548036 | Hnrnph2 |
| 10349138 | 0.02469 | 1.81E-03 | 4.8131408 | -1.061525 | 1.67153238 | Serpinb11 |
| 10514924 | 0.02469 | 1.81E-03 | 4.8129631 | -1.061744 | 1.02452532 | Tomm22 |
| 10369661 | 0.02474 | 1.82E-03 | 4.8087421 | -1.066948 | 1.02252133 | Ccar1 |
| 10395163 | 0.02474 | 1.82E-03 | 4.8062004 | -1.070083 | 1.15863206 | Lamb1 |
| 10538318 | 0.02481 | 1.83E-03 | 4.8026045 | -1.07452 | 1.29233925 | Tax1bp1 |
| 10578361 | 0.02484 | 1.83E-03 | 4.8001069 | -1.077603 | 1.51941298 | Asah1 |
| 10607116 | 0.02484 | 1.84E-03 | 4.7999456 | -1.077803 | 1.14739236 | Ammecr1 |
| 10360128 | 0.02484 | 1.84E-03 | 4.7993066 | -1.078592 | 1.43263757 | Rpl27 |
| 10486396 | 0.02486 | 1.84E-03 | 4.7969161 | -1.081544 | 1.00466482 | Ehd4 |
| 10579852 | 0.02486 | 1.84E-03 | 4.7966789 | -1.081836 | 1.17242895 | Mmaa |
| 10578069 | 0.02495 | 1.86E-03 | 4.79011 | -1.089953 | 1.78992681 | Gtpbp10///Gtpbp10 |
| 10499847 | 0.02506 | 1.87E-03 | 4.7834884 | -1.098142 | 1.12404602 | Chtop |
| 10456423 | 0.02507 | 1.87E-03 | 4.7824305 | -1.099451 | 1.02091043 | Seh1l |
| 10581824 | 0.02509 | 1.88E-03 | -4.7801184 | -1.102312 | -1.67287647 | Fa2h |
| 10452516 | 0.02511 | 1.88E-03 | 4.7786548 | -1.104124 | 1.10913525 | Ankrd12 |
| 10408850 | 0.02511 | 1.88E-03 | 4.7780792 | -1.104836 | 1.12974168 | Nedd9 |
| 10371616 | 0.02525 | 1.90E-03 | 4.7713324 | -1.113192 | 1.00807246 | Chpt1 |
| 10355017 | 0.02531 | 1.91E-03 | 4.7681479 | -1.117139 | 1.57648459 | Sumo1 |
| 10452874 | 0.02531 | 1.91E-03 | 4.7676038 | -1.117813 | 2.0863575 | Atp6v1f |
| 10594774 | 0.02533 | 1.91E-03 | -4.7652247 | -1.120763 | -1.46637487 | Ccnb2 |
| 10524018 | 0.02533 | 1.91E-03 | 4.7648668 | -1.121206 | 1.02210177 | Gm4705///Rpl34-ps1///Rpl34 |
| 10578138 | 0.02539 | 1.92E-03 | 4.7607954 | -1.126256 | 1.31143908 | Dctn6 |
| 10554819 | 0.02546 | 1.94E-03 | 4.7546215 | -1.133919 | 1.09317424 | Me3 |
| 10452556 | 0.02546 | 1.94E-03 | 4.7540337 | -1.134649 | 1.10746155 | Rab12 |
| 10451884 | 0.02547 | 1.94E-03 | 4.7534995 | -1.135312 | 1.08702589 | Camkv///Rpl21///Cep170 |
| 10590245 | 0.02548 | 1.94E-03 | 4.7520087 | -1.137164 | 1.40179839 | Slc25a38 |
| 10506870 | 0.02551 | 1.95E-03 | 4.7498257 | -1.139875 | 1.85554732 | Txndc12 |
| 10355147 | 0.02553 | 1.95E-03 | 4.7481803 | -1.14192 | 1.01664125 | Mettl21a |
| 10469575 | 0.02554 | 1.95E-03 | 4.747505 | -1.142759 | 1.83933832 | Gm13363///Ptp4a1///Gm8783 |
| 10458663 | 0.02554 | 1.95E-03 | 4.7471285 | -1.143227 | 1.25237451 | Dpysl3 |
| 10406939 | 0.02565 | 1.97E-03 | 4.7419236 | -1.149698 | 1.33558667 | Camkv///Rpl21 |
| 10503534 | 0.02568 | 1.97E-03 | 4.7400268 | -1.152057 | 1.19922965 | Ccnc |
| 10433633 | 0.02568 | 1.97E-03 | 4.7390352 | -1.15329 | 1.08312554 | Mkl2 |
| 10374197 | 0.02568 | 1.97E-03 | 4.7389945 | -1.153341 | 1.35186645 | Ramp3 |
| 10544815 | 0.02571 | 1.98E-03 | 4.7369806 | -1.155846 | 1.39358758 | Hibadh |
| 10493794 | 0.02578 | 1.99E-03 | 4.7323525 | -1.161607 | 1.08452846 | S100a14 |
| 10576152 | 0.02579 | 1.99E-03 | 4.7312121 | -1.163027 | 1.07098873 | Trappc2l |
| 10542321 | 0.02583 | 2.00E-03 | 4.7282409 | -1.166728 | 1.29198759 | Ddx47 |
| 10414288 | 0.02583 | 2.00E-03 | 4.7271729 | -1.168058 | 1.46665525 | Psmc6 |
| 10571415 | 0.02583 | 2.00E-03 | 4.7271549 | -1.168081 | 1.37914414 | Vps37a |
| 10371432 | 0.02583 | 2.00E-03 | 4.7269533 | -1.168332 | 1.01555392 | Rtcb |
| 10583203 | 0.02583 | 2.00E-03 | 4.7261913 | -1.169281 | 1.09863814 | Phxr4 |
| 10585956 | 0.02591 | 2.01E-03 | 4.7220059 | -1.174498 | 1.91550421 | Myo9a |
| 10408162 | 0.02611 | 2.04E-03 | 4.7085381 | -1.191301 | 1.05615073 | Zfp322a |
| 10577544 | 0.02615 | 2.05E-03 | 4.7062344 | -1.194179 | 1.0783929 | Polb |
| 10491601 | 0.02624 | 2.06E-03 | 4.7009522 | -1.200779 | 1.71533942 | 4932438A13Rik |
| 10503359 | 0.0264 | 2.09E-03 | 4.6919872 | -1.211991 | 1.88296178 | Rbm12b2 |
| 10486029 | 0.02643 | 2.09E-03 | 4.690215 | -1.214208 | 1.30076101 | Dph6 |
| 10523001 | 0.02651 | 2.10E-03 | 4.6855071 | -1.220102 | 1.04757864 | Mob1b |
| 10547471 | 0.02653 | 2.11E-03 | 4.6836529 | -1.222425 | 1.19926784 | B4galnt3 |
| 10368527 | 0.02665 | 2.12E-03 | 4.6788104 | -1.228492 | 1.24843381 | Hint3 |
| 10558057 | 0.02673 | 2.13E-03 | 4.6740955 | -1.234403 | 1.30258479 | Wdr11 |
| 10465278 | 0.02675 | 2.13E-03 | 4.6729502 | -1.23584 | 1.12427085 | Cdc42ep2 |
| 10482144 | 0.02681 | 2.14E-03 | 4.6701371 | -1.239369 | 1.00029804 | Rc3h2 |
| 10439881 | 0.02686 | 2.15E-03 | 4.6654846 | -1.245208 | 1.03045387 | Dubr///Dubr |
| 10585276 | 0.02686 | 2.15E-03 | -4.6647298 | -1.246156 | -1.14904292 | Pou2af1 |
| 10441339 | 0.02686 | 2.15E-03 | 4.6646749 | -1.246224 | 1.74249297 | A630089N07Rik |
| 10431732 | 0.02686 | 2.16E-03 | 4.6636231 | -1.247545 | 1.54565827 | Zcrb1 |
| 10531633 | 0.02686 | 2.16E-03 | 4.6634418 | -1.247773 | 1.13543926 | Hnrnpd |
| 10480145 | 0.02693 | 2.17E-03 | 4.6594403 | -1.252799 | 1.10968227 | Rsu1 |
| 10482467 | 0.02695 | 2.17E-03 | 4.6582888 | -1.254246 | 1.12649018 | Orc4 |
| 10416169 | 0.027 | 2.18E-03 | 4.6558001 | -1.257373 | 1.19486917 | Gnrh1 |
| 10492558 | 0.0271 | 2.19E-03 | 4.6506302 | -1.263873 | 1.07154508 | Smc4 |
| 10412260 | 0.02714 | 2.20E-03 | 4.6485891 | -1.266441 | 1.02920659 | Fst |
| 10508465 | 0.02715 | 2.20E-03 | 4.6470022 | -1.268437 | 1.0271764 | Marcksl1 |
| 10454546 | 0.02723 | 2.21E-03 | 4.6419999 | -1.274734 | 1.14802381 | Map3k2 |
| 10490773 | 0.02736 | 2.23E-03 | 4.6369818 | -1.281054 | 2.18474746 | Hnrnph2 |
| 10501762 | 0.02741 | 2.24E-03 | 4.6340266 | -1.284777 | 1.04781564 | Snx7 |
| 10531919 | 0.02742 | 2.24E-03 | 4.6327932 | -1.286332 | 1.15950146 | Hsd17b11 |
| 10363000 | 0.02742 | 2.24E-03 | 4.6321046 | -1.2872 | 1.08593695 | Gpx4 |
| 10455578 | 0.02745 | 2.24E-03 | 4.6304976 | -1.289226 | 1.01514401 | Commd10 |
| 10432509 | 0.02745 | 2.25E-03 | 4.6280991 | -1.292251 | 1.11635089 | Uxt |
| 10407416 | 0.02746 | 2.26E-03 | 4.6264789 | -1.294294 | 1.44925943 | Calml3 |
| 10569278 | 0.02747 | 2.26E-03 | 4.625995 | -1.294904 | 1.04431985 | Dusp8 |
| 10408450 | 0.02752 | 2.27E-03 | 4.622884 | -1.29883 | 1.1703411 | Sox4 |
| 10474588 | 0.02752 | 2.27E-03 | 4.6227811 | -1.29896 | 1.58649986 | Emc7 |
| 10417757 | 0.02752 | 2.27E-03 | 4.6224578 | -1.299368 | 1.13211597 | Vps4b |
| 10476775 | 0.02759 | 2.27E-03 | 4.6201417 | -1.302291 | 1.93933077 | Naa20 |
| 10445898 | 0.02762 | 2.28E-03 | 4.6181643 | -1.304788 | 1.22198575 | Rab5a |
| 10397346 | 0.02762 | 2.28E-03 | -4.6179591 | -1.305047 | -1.31855266 | Fos |
| 10608482 | 0.02765 | 2.29E-03 | -4.6155113 | -1.308139 | -2.52308446 | Gm20831///Ssty1 |
| 10595205 | 0.02769 | 2.29E-03 | 4.612852 | -1.311498 | 1.10152049 | Carnmt1 |
| 10415784 | 0.02773 | 2.30E-03 | 4.610672 | -1.314253 | 1.95982577 | Trim13 |
| 10458213 | 0.02781 | 2.31E-03 | 4.606675 | -1.319306 | 1.40645255 | Etf1 |
| 10351119 | 0.02781 | 2.31E-03 | 4.6065321 | -1.319487 | 1.29781694 | Vamp4 |
| 10598507 | 0.02781 | 2.31E-03 | 4.606266 | -1.319823 | 1.10321999 | Slc38a5 |
| 10418879 | 0.02782 | 2.32E-03 | 4.604746 | -1.321746 | 1.01590642 | Mapk8 |
| 10463951 | 0.02784 | 2.32E-03 | 4.6031362 | -1.323782 | 1.4569731 | Smc3 |
| 10482687 | 0.02786 | 2.33E-03 | 4.6013451 | -1.326048 | 1.1359649 | Arl5a |
| 10545479 | 0.028 | 2.34E-03 | 4.5946833 | -1.334481 | 1.3145616 | Tmsb10 |
| 10400191 | 0.028 | 2.35E-03 | 4.5941062 | -1.335212 | 1.1526355 | Strn3 |
| 10404538 | 0.02804 | 2.35E-03 | 4.5920438 | -1.337824 | 1.00961068 | Prpf4b |
| 10369647 | 0.02806 | 2.36E-03 | 4.5901169 | -1.340266 | 1.77645365 | Ddx50 |
| 10453049 | 0.02809 | 2.36E-03 | 4.5887732 | -1.341968 | 1.49308673 | Cdc42ep3 |
| 10554034 | 0.02809 | 2.36E-03 | 4.5886255 | -1.342155 | 1.03816049 | Cers3 |
| 10599893 | 0.02809 | 2.36E-03 | 4.5881427 | -1.342767 | 1.15502335 | Fmr1 |
| 10547793 | 0.02811 | 2.37E-03 | -4.5855678 | -1.346031 | -1.02901703 | Rnu7 |
| 10536041 | 0.02811 | 2.37E-03 | -4.5848604 | -1.346928 | -1.1578687 | Gm3259///Gm6367///C87414///AA792892 |
| 10526743 | 0.02811 | 2.37E-03 | 4.5847642 | -1.34705 | 1.27486849 | Cops6 |
| 10514072 | 0.02813 | 2.38E-03 | 4.582411 | -1.350034 | 1.2903539 | Zdhhc21 |
| 10388996 | 0.02825 | 2.39E-03 | 4.5779163 | -1.355736 | 1.57188499 | Crlf3 |
| 10367436 | 0.02831 | 2.40E-03 | 4.5749131 | -1.359548 | 1.88329893 | Cd63 |
| 10602795 | 0.02832 | 2.40E-03 | 4.5738343 | -1.360918 | 1.29165757 | Eif1ax |
| 10606876 | 0.02832 | 2.40E-03 | 4.5737224 | -1.36106 | 1.20872376 | Morf4l2 |
| 10349694 | 0.02833 | 2.41E-03 | 4.5728288 | -1.362194 | 1.01030043 | Pm20d1 |
| 10568714 | 0.02837 | 2.42E-03 | -4.5690419 | -1.367004 | -1.23762737 | Mki67 |
| 10593413 | 0.02837 | 2.42E-03 | 4.5686431 | -1.36751 | 1.23800977 | 2310030G06Rik |
| 10570614 | 0.02848 | 2.45E-03 | -4.5582806 | -1.380684 | -1.27336296 | Defb6 |
| 10468309 | 0.02857 | 2.46E-03 | 4.5555292 | -1.384184 | 1.18731543 | Sh3pxd2a |
| 10521555 | 0.02857 | 2.46E-03 | 4.555084 | -1.38475 | 1.088284 | Lyar |
| 10399036 | 0.02857 | 2.46E-03 | 4.5546346 | -1.385322 | 1.20393492 | Uevld |
| 10420439 | 0.02857 | 2.46E-03 | 4.5535901 | -1.386652 | 1.22311009 | Zdhhc20 |
| 10452815 | 0.0286 | 2.47E-03 | 4.5512451 | -1.389637 | 1.39774476 | Xdh |
| 10471953 | 0.02862 | 2.48E-03 | 4.5495949 | -1.391738 | 1.0319194 | Acvr2a |
| 10541216 | 0.02862 | 2.48E-03 | 4.5485878 | -1.39302 | 1.07839871 | Kdm5a |
| 10515836 | 0.02866 | 2.49E-03 | -4.5452408 | -1.397284 | -1.01524866 | Ccnb1 |
| 10428690 | 0.02866 | 2.49E-03 | 4.544691 | -1.397984 | 1.4342339 | Mrpl13 |
| 10414514 | 0.02866 | 2.49E-03 | 4.5431331 | -1.399969 | 1.47180824 | Pnp |
| 10606315 | 0.02876 | 2.51E-03 | 4.5365281 | -1.40839 | 1.14918233 | Taf9b |
| 10442643 | 0.02876 | 2.52E-03 | 4.5364147 | -1.408534 | 1.32755723 | Nme3 |
| 10585201 | 0.02876 | 2.52E-03 | 4.5356474 | -1.409513 | 1.01390551 | Timm8b |
| 10457465 | 0.02876 | 2.52E-03 | 4.5352508 | -1.410019 | 1.00887986 | Esco1 |
| 10349016 | 0.02876 | 2.52E-03 | 4.5351212 | -1.410184 | 1.00593813 | 2310035C23Rik |
| 10406193 | 0.02879 | 2.52E-03 | 4.5333478 | -1.412447 | 1.21273085 | Ccdc127 |
| 10596982 | 0.02885 | 2.53E-03 | 4.5310681 | -1.415356 | 1.54923728 | Tma7 |
| 10582997 | 0.02898 | 2.55E-03 | -4.5258613 | -1.422003 | -1.33150397 | Casp4 |
| 10603373 | 0.02899 | 2.55E-03 | 4.5253241 | -1.422689 | 1.03736824 | Pqbp1 |
| 10511881 | 0.02899 | 2.55E-03 | 4.5240687 | -1.424292 | 1.34315574 | Manea |
| 10443459 | 0.02899 | 2.55E-03 | 4.5239901 | -1.424393 | 1.19608237 | Srsf3 |
| 10366310 | 0.02904 | 2.57E-03 | 4.5187717 | -1.43106 | 1.01911295 | Osbpl8 |
| 10511541 | 0.02907 | 2.58E-03 | 4.5169149 | -1.433434 | 1.05642021 | Dpy19l4 |
| 10472589 | 0.02915 | 2.59E-03 | 4.5130084 | -1.438429 | 1.38762054 | Phospho2 |
| 10553993 | 0.02915 | 2.59E-03 | 4.5127849 | -1.438715 | 1.45026547 | Snrpa1 |
| 10566326 | 0.02915 | 2.59E-03 | 4.5125773 | -1.438981 | 1.03909067 | Trim12a |
| 10469083 | 0.02915 | 2.60E-03 | 4.5103107 | -1.44188 | 1.65372457 | Upf2 |
| 10380501 | 0.02915 | 2.60E-03 | 4.5093884 | -1.44306 | 1.42074528 | Dlx3 |
| 10517328 | 0.02915 | 2.60E-03 | 4.5078989 | -1.444967 | 1.10746602 | Tmem50a |
| 10408677 | 0.02915 | 2.61E-03 | 4.5071042 | -1.445984 | 1.20704925 | Lyrm4 |
| 10506786 | 0.02915 | 2.61E-03 | 4.5066712 | -1.446538 | 1.56039716 | Zcchc11 |
| 10344803 | 0.02944 | 2.66E-03 | 4.4914034 | -1.466099 | 1.62686282 | Cspp1 |
| 10595189 | 0.02944 | 2.66E-03 | 4.4913018 | -1.466229 | 1.06194856 | Slc17a5 |
| 10580370 | 0.02944 | 2.66E-03 | 4.4906271 | -1.467094 | 1.32340518 | Dnaja2 |
| 10583347 | 0.02951 | 2.67E-03 | 4.4875385 | -1.471056 | 1.58383913 | Chordc1 |
| 10487277 | 0.02955 | 2.68E-03 | 4.4856515 | -1.473477 | 1.47917467 | Trpm7 |
| 10367973 | 0.02969 | 2.70E-03 | 4.47721 | -1.484315 | 1.00793935 | Aig1 |
| 10596543 | 0.02978 | 2.72E-03 | 4.4721711 | -1.49079 | 1.46845891 | Rad54l2 |
| 10466441 | 0.02978 | 2.72E-03 | 4.4719731 | -1.491045 | 1.25263434 | Vps13a |
| 10521589 | 0.02978 | 2.72E-03 | 4.4718147 | -1.491248 | 1.5009802 | Cir1 |
| 10357472 | 0.02978 | 2.72E-03 | 4.4716099 | -1.491512 | 1.4181247 | Cxcr4 |
| 10490611 | 0.02978 | 2.73E-03 | -4.4699392 | -1.493659 | -1.19397399 | Ptk6 |
| 10344707 | 0.02978 | 2.73E-03 | 4.4698181 | -1.493815 | 1.0219265 | Pcmtd1 |
| 10366707 | 0.02978 | 2.73E-03 | -4.4691416 | -1.494685 | -1.28474845 | Avpr1a |
| 10407907 | 0.02978 | 2.73E-03 | 4.4690542 | -1.494797 | 1.71136643 | Rala |
| 10524079 | 0.0298 | 2.73E-03 | 4.4678377 | -1.496362 | 1.35387109 | Gm15446///Zfp932 |
| 10436708 | 0.02982 | 2.74E-03 | 4.4657923 | -1.498992 | 1.07232763 | Usp16 |
| 10397683 | 0.02983 | 2.75E-03 | 4.4646407 | -1.500474 | 1.19986735 | Ttc8 |
| 10550383 | 0.02984 | 2.75E-03 | -4.4634994 | -1.501942 | -1.07213931 | Dact3 |
| 10469312 | 0.02985 | 2.75E-03 | 4.4623887 | -1.503371 | 1.10359546 | Pter |
| 10585417 | 0.03 | 2.78E-03 | 4.4556687 | -1.512022 | 1.08109527 | Idh3a |
| 10492598 | 0.03001 | 2.78E-03 | 4.4552901 | -1.51251 | 1.51345293 | Nmd3 |
| 10413803 | 0.03003 | 2.78E-03 | 4.4544269 | -1.513622 | 1.20394487 | Btd |
| 10362794 | 0.03005 | 2.79E-03 | 4.452595 | -1.515982 | 1.11222212 | Ppil6 |
| 10395389 | 0.03005 | 2.79E-03 | 4.4523005 | -1.516361 | 1.01683367 | Sostdc1 |
| 10552314 | 0.03005 | 2.79E-03 | 4.4520024 | -1.516745 | 1.03677638 | 2610021A01Rik///Zfp141 |
| 10389245 | 0.03005 | 2.79E-03 | 4.4519514 | -1.516811 | 1.09404994 | Tada2a |
| 10572253 | 0.03005 | 2.79E-03 | 4.4514951 | -1.517399 | 1.13749925 | Sugp1 |
| 10436773 | 0.03005 | 2.79E-03 | 4.4512491 | -1.517716 | 1.27095842 | Gm35427///Gm35174///Gm35004///Gm34826///Gm34733///Gm9789 |
| 10365933 | 0.03015 | 2.81E-03 | 4.4454987 | -1.525128 | 1.02790249 | Eea1 |
| 10572813 | 0.03015 | 2.81E-03 | 4.4448388 | -1.525979 | 1.52164326 | Usmg5 |
| 10457429 | 0.03015 | 2.82E-03 | 4.4440972 | -1.526936 | 1.39163638 | Rock1 |
| 10479979 | 0.03016 | 2.82E-03 | 4.4431928 | -1.528102 | 1.2436651 | Slc25a36 |
| 10555892 | 0.03023 | 2.83E-03 | 4.4400855 | -1.532111 | 1.19454636 | Twf1 |
| 10413695 | 0.03023 | 2.83E-03 | 4.4398524 | -1.532411 | 1.15598167 | Pbrm1 |
| 10413434 | 0.03027 | 2.84E-03 | 4.4379163 | -1.53491 | 1.09917728 | Fam208a |
| 10521587 | 0.03027 | 2.84E-03 | 4.4372034 | -1.53583 | 1.1108388 | Dnaja1 |
| 10604799 | 0.03027 | 2.84E-03 | 4.4369757 | -1.536124 | 1.52578943 | Atp11c |
| 10549473 | 0.03027 | 2.84E-03 | 4.4369667 | -1.536136 | 1.03208809 | Caprin2 |
| 10357124 | 0.03027 | 2.84E-03 | 4.4366435 | -1.536553 | 1.09060385 | Tsn |
| 10590654 | 0.03029 | 2.84E-03 | 4.4357114 | -1.537756 | 1.07617318 | Aasdhppt |
| 10381872 | 0.0303 | 2.85E-03 | 4.434635 | -1.539146 | 1.0189154 | Tlk2 |
| 10395328 | 0.03048 | 2.88E-03 | 4.4265448 | -1.549596 | 1.65373749 | Snx13 |
| 10445875 | 0.03056 | 2.89E-03 | 4.4228139 | -1.554419 | 1.1405437 | Gm7334///Btg3 |
| 10429564 | 0.03058 | 2.89E-03 | -4.4215969 | -1.555993 | -1.02106242 | Ly6a |
| 10421672 | 0.03058 | 2.90E-03 | 4.4211821 | -1.556529 | 1.07944833 | Gpalpp1 |
| 10503134 | 0.0306 | 2.90E-03 | 4.4203295 | -1.557632 | 1.50917394 | Sdcbp |
| 10594636 | 0.03068 | 2.91E-03 | 4.4172207 | -1.561653 | 1.42334993 | Ppp1r2-ps3///Ppp1r2 |
| 10412251 | 0.0307 | 2.91E-03 | 4.4164151 | -1.562696 | 1.18872035 | Ndufs4 |
| 10544148 | 0.03073 | 2.92E-03 | 4.4148568 | -1.564712 | 1.5290837 | Kdm7a |
| 10497831 | 0.03078 | 2.92E-03 | -4.4131642 | -1.566903 | -1.66290524 | Ccna2 |
| 10349733 | 0.03083 | 2.93E-03 | 4.4113665 | -1.56923 | 1.27067718 | Nucks1 |
| 10546606 | 0.03088 | 2.94E-03 | 4.4096432 | -1.571462 | 2.20578822 | Uba3 |
| 10358849 | 0.03088 | 2.94E-03 | 4.4095433 | -1.571591 | 1.02184168 | Dhx9 |
| 10501629 | 0.03088 | 2.94E-03 | 4.4092679 | -1.571948 | 1.13377898 | Cdc14a |
| 10587266 | 0.03089 | 2.95E-03 | 4.4074295 | -1.574329 | 1.16887579 | Gclc |
| 10467256 | 0.03089 | 2.95E-03 | 4.4072013 | -1.574625 | 1.23189185 | Rpl10 |
| 10454809 | 0.03089 | 2.95E-03 | 4.4067975 | -1.575148 | 1.6023208 | Matr3 |
| 10463153 | 0.03096 | 2.96E-03 | 4.4023361 | -1.580929 | 1.13685646 | Morf4l1-ps1///Morf4l1 |
| 10351623 | 0.03101 | 2.97E-03 | 4.3996323 | -1.584434 | 1.0805936 | F11r |
| 10497399 | 0.03101 | 2.97E-03 | 4.3995004 | -1.584605 | 1.10009117 | Pde7a |
| 10606160 | 0.03107 | 2.99E-03 | 4.395588 | -1.589678 | 1.04153506 | Rfwd2 |
| 10359446 | 0.03118 | 3.01E-03 | 4.39064 | -1.596099 | 1.37251335 | Suco |
| 10512851 | 0.03118 | 3.01E-03 | 4.3889168 | -1.598335 | 1.54531486 | Erp44 |
| 10358359 | 0.03125 | 3.03E-03 | 4.3858537 | -1.602313 | 1.3295533 | Cdc73 |
| 10565514 | 0.03129 | 3.03E-03 | 4.3838649 | -1.604896 | 1.18832742 | Tmem126a |
| 10359713 | 0.03132 | 3.04E-03 | 4.3819006 | -1.607448 | 1.30968586 | Sft2d2 |
| 10408094 | 0.03132 | 3.04E-03 | -4.3815488 | -1.607905 | -1.2443433 | Hist1h2al///Hist1h2ao///Hist2h2ab///Hist2h2aa2///Hist1h2ai///Hist2h2ac///Hist1h2af///Hist1h2ab///Hist1h2ap///Hist1h2an///Hist1h2ak///Hist1h2ah///Hist1h2ag///Hist1h2ae///Hist1h2ad///Hist1h2ac///Hist1h2aa///Hist3h2a///H2afj///H2afx///Hist2h2aa1///Hist1h3d |
| 10468881 | 0.03139 | 3.05E-03 | 4.378448 | -1.611934 | 1.03031854 | Zfp950///Arid4b |
| 10361979 | 0.03139 | 3.05E-03 | 4.3782618 | -1.612176 | 1.69468991 | Bclaf1 |
| 10477353 | 0.03139 | 3.06E-03 | 4.3767074 | -1.614197 | 1.73393049 | Mapre1 |
| 10486324 | 0.03147 | 3.07E-03 | -4.3732248 | -1.618726 | -1.00003692 | Gm13999 |
| 10351043 | 0.0315 | 3.08E-03 | -4.3720125 | -1.620302 | -1.19289392 | Snord47 |
| 10379779 | 0.03164 | 3.10E-03 | 4.365591 | -1.628659 | 1.13396257 | Ddx52 |
| 10606436 | 0.03164 | 3.10E-03 | 4.365474 | -1.628811 | 1.34206943 | Hmgn5 |
| 10498319 | 0.03182 | 3.13E-03 | 4.3592675 | -1.636893 | 1.5864991 | Serp1 |
| 10491083 | 0.03197 | 3.16E-03 | 4.3506326 | -1.648148 | 1.09015532 | Nceh1 |
| 10604424 | 0.03198 | 3.16E-03 | 4.3498256 | -1.649201 | 1.36506309 | Zfp280c |
| 10455335 | 0.032 | 3.17E-03 | -4.348855 | -1.650467 | -1.09308819 | Pou4f3 |
| 10399666 | 0.03201 | 3.17E-03 | 4.3483044 | -1.651185 | 1.01217686 | Gm9257///9030624G23Rik |
| 10430811 | 0.03201 | 3.17E-03 | 4.347967 | -1.651625 | 1.43007417 | Nhp2l1 |
| 10447038 | 0.0321 | 3.19E-03 | 4.3425021 | -1.658756 | 1.64864024 | Cebpzos |
| 10558436 | 0.03224 | 3.22E-03 | 4.3353606 | -1.668082 | 1.01464355 | BC005624 |
| 10436849 | 0.0323 | 3.23E-03 | 4.3328897 | -1.671311 | 1.14555786 | Ifnar1 |
| 10450431 | 0.03239 | 3.25E-03 | -4.3286942 | -1.676795 | -1.03467432 | Ly6g5b///Csnk2b |
| 10441787 | 0.03241 | 3.25E-03 | 4.3279645 | -1.677749 | 1.19533533 | Airn |
| 10601312 | 0.03241 | 3.25E-03 | 4.3278165 | -1.677942 | 1.12256304 | Chic1 |
| 10465831 | 0.03244 | 3.26E-03 | -4.3267468 | -1.679341 | -1.04173419 | 5730408K05Rik |
| 10471675 | 0.03252 | 3.27E-03 | 4.3233587 | -1.683773 | 1.26293608 | Glo1 |
| 10447349 | 0.03255 | 3.28E-03 | 4.3207293 | -1.687213 | 1.63931711 | Cript |
| 10404630 | 0.03258 | 3.28E-03 | 4.3196542 | -1.68862 | 1.04174151 | Riok1 |
| 10507885 | 0.03258 | 3.29E-03 | 4.3193081 | -1.689073 | 1.05102681 | Mycbp |
| 10545697 | 0.03263 | 3.30E-03 | 4.3156252 | -1.693895 | 1.0095552 | Dguok |
| 10532157 | 0.03276 | 3.32E-03 | 4.3109483 | -1.70002 | 1.08237177 | Tmed5 |
| 10396064 | 0.03279 | 3.32E-03 | 4.3099871 | -1.70128 | 1.22291906 | Txndc9 |
| 10577534 | 0.03284 | 3.34E-03 | 4.3073328 | -1.704758 | 1.33188698 | Vdac3 |
| 10407192 | 0.03284 | 3.34E-03 | 4.3069234 | -1.705295 | 1.16608352 | Slc38a9 |
| 10378114 | 0.03289 | 3.36E-03 | 4.302158 | -1.711543 | 1.24545764 | Ube2g1 |
| 10424113 | 0.03289 | 3.36E-03 | 4.3016982 | -1.712146 | 1.39292747 | Mal2 |
| 10373610 | 0.03291 | 3.36E-03 | -4.3003885 | -1.713864 | -1.365287 | Olfr767 |
| 10536061 | 0.03291 | 3.37E-03 | 4.3002502 | -1.714045 | 2.73870782 | 2610021A01Rik///Zfp141 |
| 10365408 | 0.03291 | 3.37E-03 | 4.2997778 | -1.714665 | 1.11147317 | Ric8b |
| 10449999 | 0.03296 | 3.38E-03 | 4.2961387 | -1.71944 | 1.58428921 | Zfp101 |
| 10406426 | 0.03296 | 3.39E-03 | 4.2954245 | -1.720378 | 1.32248499 | Cetn3 |
| 10514383 | 0.03296 | 3.39E-03 | 4.2949476 | -1.721004 | 1.00427121 | Larp7 |
| 10398124 | 0.03307 | 3.42E-03 | 4.2864676 | -1.73214 | 1.09504608 | Gskip |
| 10558263 | 0.03308 | 3.43E-03 | -4.2859718 | -1.732792 | -1.05733752 | Gm10584 |
| 10530827 | 0.03308 | 3.43E-03 | 4.2851061 | -1.733929 | 1.14372196 | Spink2 |
| 10581473 | 0.03316 | 3.44E-03 | 4.2816217 | -1.738509 | 1.15382997 | Slc7a6os |
| 10419354 | 0.03316 | 3.45E-03 | 4.2813343 | -1.738887 | 1.34093776 | Map1lc3b///Map1lc3b |
| 10418210 | 0.03319 | 3.45E-03 | 4.2805215 | -1.739955 | 1.02680994 | Tmem254b///Tmem254c///Tmem254a |
| 10565072 | 0.03321 | 3.46E-03 | 4.2779248 | -1.74337 | 1.11421463 | Sec11a |
| 10523579 | 0.03331 | 3.47E-03 | 4.2750801 | -1.747112 | 1.14271842 | Arhgap24 |
| 10587683 | 0.03339 | 3.49E-03 | -4.2719862 | -1.751183 | -1.94483336 | Bcl2a1d///Bcl2a1c///Bcl2a1b///Bcl2a1a |
| 10576692 | 0.03341 | 3.49E-03 | 4.2703072 | -1.753393 | 1.03123647 | Insr |
| 10421685 | 0.0335 | 3.51E-03 | -4.2660699 | -1.758973 | -1.00268161 | Serp2 |
| 10579799 | 0.03351 | 3.52E-03 | 4.2642816 | -1.761328 | 1.60666925 | Tmem184c |
| 10407358 | 0.03358 | 3.53E-03 | 4.2624017 | -1.763805 | 1.39717653 | Paip1 |
| 10407766 | 0.03372 | 3.56E-03 | 4.2555199 | -1.772876 | 1.08799965 | Lgals8 |
| 10345981 | 0.03372 | 3.56E-03 | 4.25538 | -1.77306 | 1.03428967 | Ercc5 |
| 10485514 | 0.03374 | 3.57E-03 | 4.2532155 | -1.775915 | 1.2074844 | Caprin1 |
| 10546430 | 0.03374 | 3.57E-03 | 4.2530446 | -1.77614 | 1.18548722 | Adamts9 |
| 10522676 | 0.03387 | 3.60E-03 | 4.2454262 | -1.786193 | 1.09224979 | Srp72 |
| 10518455 | 0.03387 | 3.61E-03 | 4.2444844 | -1.787437 | 1.53571583 | Agtrap |
| 10568361 | 0.03388 | 3.62E-03 | 4.2422922 | -1.790332 | 1.46614124 | Yipf5 |
| 10545339 | 0.03401 | 3.64E-03 | 4.2386889 | -1.795091 | 1.18355644 | Mrpl35 |
| 10504499 | 0.03403 | 3.64E-03 | 4.2369462 | -1.797394 | 1.00714139 | Zcchc7 |
| 10605081 | 0.03403 | 3.65E-03 | 4.2358079 | -1.798898 | 1.05762225 | Bcap31 |
| 10395142 | 0.03409 | 3.66E-03 | 4.233826 | -1.801518 | 1.03336647 | Sh3yl1 |
| 10503926 | 0.03413 | 3.67E-03 | 4.2308865 | -1.805405 | 1.09587143 | Rars2 |
| 10464479 | 0.03416 | 3.69E-03 | 4.2277157 | -1.809598 | 1.00962509 | Ppp6r3 |
| 10418004 | 0.03417 | 3.69E-03 | 4.2269341 | -1.810632 | 1.32401489 | Ap3m1 |
| 10403229 | 0.03427 | 3.71E-03 | 4.2233501 | -1.815375 | 1.38069472 | Itgb8 |
| 10550059 | 0.03428 | 3.71E-03 | 4.2221664 | -1.816942 | 1.00322362 | Rnf225 |
| 10435963 | 0.03428 | 3.72E-03 | 4.2204455 | -1.81922 | 1.83993471 | Atg3 |
| 10490078 | 0.03428 | 3.73E-03 | 4.2179857 | -1.822477 | 1.59446592 | Sumo1 |
| 10524082 | 0.03429 | 3.74E-03 | 4.2172515 | -1.823449 | 2.00148358 | Gm15446///Zfp932 |
| 10399897 | 0.03432 | 3.74E-03 | 4.2154098 | -1.825889 | 1.03408648 | Hbp1 |
| 10386159 | 0.03432 | 3.75E-03 | 4.2147681 | -1.826739 | 1.29883312 | Zfp672 |
| 10449914 | 0.03433 | 3.75E-03 | 4.2136134 | -1.828269 | 1.02000216 | Zfp871 |
| 10426301 | 0.03435 | 3.76E-03 | 4.2126919 | -1.82949 | 1.50324271 | Tcea1 |
| 10495574 | 0.03444 | 3.77E-03 | 4.2091554 | -1.834177 | 1.28643153 | Sass6 |
| 10432799 | 0.03446 | 3.79E-03 | 4.2062512 | -1.838028 | 1.41898198 | Krt71 |
| 10367772 | 0.03448 | 3.79E-03 | 4.2049313 | -1.839779 | 1.33351972 | Samd5 |
| 10440568 | 0.03459 | 3.83E-03 | 4.1975579 | -1.849562 | 1.42808302 | Ltn1 |
| 10492381 | 0.03459 | 3.83E-03 | 4.1973237 | -1.849873 | 2.20429666 | Gmps |
| 10469943 | 0.03462 | 3.85E-03 | -4.1943113 | -1.853873 | -1.00448151 | Fam166a |
| 10453918 | 0.03466 | 3.85E-03 | 4.1928045 | -1.855874 | 1.13163944 | 3110002H16Rik |
| 10379535 | 0.03468 | 3.86E-03 | -4.1915805 | -1.8575 | -1.01129067 | Ccl8 |
| 10493137 | 0.03474 | 3.87E-03 | -4.1888128 | -1.861177 | -1.32935951 | Iqgap3 |
| 10514255 | 0.03474 | 3.87E-03 | 4.1887135 | -1.861309 | 1.17427798 | Mllt3 |
| 10477604 | 0.03474 | 3.87E-03 | 4.1885305 | -1.861552 | 1.01877736 | Itch |
| 10374983 | 0.03476 | 3.88E-03 | 4.1875528 | -1.862851 | 1.0329218 | Asb3 |
| 10569996 | 0.03477 | 3.88E-03 | 4.1868286 | -1.863814 | 1.15635193 | Rpl21 |
| 10429568 | 0.03495 | 3.92E-03 | -4.1788675 | -1.874399 | -1.16398507 | Ly6c2///Ly6c1 |
| 10571621 | 0.03501 | 3.93E-03 | 4.1765589 | -1.877471 | 1.68856402 | Ufsp2 |
| 10556246 | 0.03512 | 3.96E-03 | 4.1711812 | -1.884629 | 1.04574467 | Zfp143 |
| 10467091 | 0.03512 | 3.97E-03 | 4.169443 | -1.886943 | 1.13207111 | Atad1 |
| 10599377 | 0.03512 | 3.97E-03 | 4.1691127 | -1.887383 | 1.44676835 | Stag2 |
| 10578539 | 0.03512 | 3.97E-03 | 4.1686961 | -1.887938 | 1.44818896 | Slc25a4 |
| 10441489 | 0.03515 | 3.99E-03 | 4.1653464 | -1.8924 | 1.04507241 | Gtf2h5 |
| 10586484 | 0.03515 | 3.99E-03 | 4.16513 | -1.892688 | 1.05175189 | Fam96a |
| 10507784 | 0.03521 | 4.01E-03 | 4.1618663 | -1.897038 | 1.03454578 | Ppt1 |
| 10552406 | 0.0353 | 4.02E-03 | -4.1583314 | -1.90175 | -1.71525443 | Nkg7 |
| 10491623 | 0.03533 | 4.03E-03 | 4.1573191 | -1.9031 | 1.14753287 | 4932438A13Rik |
| 10608282 | 0.03539 | 4.05E-03 | -4.1537605 | -1.907846 | -1.70265522 | Gm20917///Gm20877///Gm20865///Gm20854///Gm20852///Gm20815///Gm20809///Gm20806///Gm20747///Gm21943///Gm20738///Ssty2 |
| 10509568 | 0.03539 | 4.05E-03 | 4.1534754 | -1.908227 | 1.18261713 | Camk2n1 |
| 10374793 | 0.03539 | 4.05E-03 | 4.1533189 | -1.908436 | 1.02194591 | Pnpt1 |
| 10452892 | 0.0354 | 4.05E-03 | 4.1524792 | -1.909556 | 1.0115785 | Fam98a |
| 10473008 | 0.03545 | 4.06E-03 | 4.1510389 | -1.911478 | 1.49160724 | Gm6793///Hnrnpa3 |
| 10380260 | 0.03557 | 4.11E-03 | 4.1427021 | -1.922608 | 1.34456394 | Trim25 |
| 10380238 | 0.03557 | 4.11E-03 | 4.1422263 | -1.923244 | 1.09577982 | Mrps23 |
| 10445214 | 0.03557 | 4.11E-03 | 4.1409924 | -1.924892 | 1.07505033 | Mut |
| 10413710 | 0.03579 | 4.16E-03 | 4.132394 | -1.936385 | 1.17877838 | Nt5dc2 |
| 10357454 | 0.0358 | 4.16E-03 | 4.1318951 | -1.937053 | 1.14091817 | Dars |
| 10492671 | 0.0358 | 4.16E-03 | 4.1315941 | -1.937455 | 1.07568327 | Ppid |
| 10362442 | 0.03586 | 4.19E-03 | -4.1263622 | -1.944454 | -1.13849804 | Trdn |
| 10406541 | 0.03591 | 4.21E-03 | -4.122775 | -1.949256 | -1.69749575 | Gm8624///Rps23 |
| 10472764 | 0.03592 | 4.21E-03 | 4.1221104 | -1.950145 | 1.07175044 | Dync1i2 |
| 10416437 | 0.03604 | 4.24E-03 | -4.116557 | -1.957583 | -1.14692832 | Lcp1 |
| 10446804 | 0.03609 | 4.26E-03 | 4.1136988 | -1.961413 | 1.03417383 | Slc30a6 |
| 10482731 | 0.0361 | 4.27E-03 | 4.1117258 | -1.964057 | 1.44162344 | Prpf40a |
| 10506571 | 0.0361 | 4.27E-03 | -4.1112251 | -1.964728 | -1.42301867 | Dhcr24 |
| 10601449 | 0.0361 | 4.28E-03 | 4.1107361 | -1.965384 | 1.12224985 | Sh3bgrl |
| 10427148 | 0.03612 | 4.29E-03 | 4.1083993 | -1.968517 | 1.01523983 | Zfp740 |
| 10587486 | 0.03619 | 4.30E-03 | 4.105595 | -1.972277 | 1.18374228 | BC085271///Set |
| 10373778 | 0.03628 | 4.33E-03 | 4.101707 | -1.977494 | 1.09654952 | Morc2a |
| 10427628 | 0.03631 | 4.34E-03 | -4.0991041 | -1.980987 | -1.13905777 | Il7r |
| 10396795 | 0.03631 | 4.34E-03 | 4.0990403 | -1.981072 | 1.25821654 | Eif2s1 |
| 10585980 | 0.03638 | 4.36E-03 | 4.0954515 | -1.985891 | 1.42550699 | Myo9a |
| 10423963 | 0.03643 | 4.38E-03 | 4.0921894 | -1.990272 | 1.25337164 | Eny2 |
| 10495625 | 0.03645 | 4.39E-03 | 4.091076 | -1.991768 | 1.10548672 | Dpyd |
| 10475981 | 0.03645 | 4.39E-03 | 4.0907817 | -1.992163 | 1.07927258 | Chchd5 |
| 10367717 | 0.03651 | 4.40E-03 | 4.0882007 | -1.995631 | 1.74049063 | Ginm1 |
| 10475866 | 0.03652 | 4.40E-03 | 4.0876308 | -1.996397 | 1.00786762 | Bcl2l11 |
| 10380065 | 0.03668 | 4.44E-03 | -4.0819371 | -2.004052 | -1.03296841 | Rnu3b4///Rnu3b3///Rnu3b2///Rnu3b1 |
| 10368011 | 0.03682 | 4.46E-03 | 4.0782545 | -2.009005 | 1.0627059 | Vta1 |
| 10388461 | 0.03701 | 4.50E-03 | 4.0715261 | -2.018061 | 1.70153039 | Gm12338///Cox7c |
| 10603953 | 0.03703 | 4.50E-03 | -4.0707047 | -2.019167 | -1.10117517 | Gm4297///Gm10230///Gm14632///Gm16430///Gm16405///Gm14819///Gm10486///Gm10487///Gm10096///Gm10147///Gm10058///Gm14525///Gm2030///Gm16404///Slx///Gm6121///Gm5934///Gm5169///Gm5168///Slxl1///Gm4836 |
| 10549402 | 0.03711 | 4.52E-03 | 4.0678468 | -2.023016 | 1.07847634 | Ergic2 |
| 10400137 | 0.03714 | 4.53E-03 | 4.0666101 | -2.024682 | 1.57846352 | Dnajb9 |
| 10346960 | 0.03718 | 4.54E-03 | 4.0641053 | -2.028057 | 1.02008098 | Ccnyl1 |
| 10462363 | 0.03718 | 4.54E-03 | 4.0639031 | -2.028329 | 1.12430665 | Jak2 |
| 10583034 | 0.03723 | 4.55E-03 | 4.0618701 | -2.031069 | 1.05065983 | Dcun1d5 |
| 10459153 | 0.03727 | 4.57E-03 | -4.0591712 | -2.034707 | -1.09930124 | Cdx1 |
| 10475264 | 0.03733 | 4.59E-03 | 4.0556629 | -2.039438 | 1.29041324 | Ccndbp1 |
| 10475435 | 0.03737 | 4.60E-03 | -4.0542083 | -2.0414 | -1.73519927 | Rps12 |
| 10369867 | 0.03737 | 4.60E-03 | 4.0540368 | -2.041632 | 1.14752695 | Tfam |
| 10395984 | 0.03739 | 4.60E-03 | 4.0533796 | -2.042518 | 1.20547022 | Fam179b |
| 10376392 | 0.03739 | 4.61E-03 | -4.0515926 | -2.044929 | -1.03146618 | Olfr325 |
| 10357051 | 0.03744 | 4.62E-03 | 4.0500852 | -2.046964 | 1.03024155 | Kdsr |
| 10389606 | 0.03746 | 4.63E-03 | -4.0494161 | -2.047867 | -1.11450227 | Prr11 |
| 10350646 | 0.03748 | 4.63E-03 | 4.0487597 | -2.048753 | 1.02433132 | Edem3 |
| 10455007 | 0.0375 | 4.64E-03 | 4.04697 | -2.051169 | 1.1282046 | Zmat2 |
| 10490802 | 0.03752 | 4.66E-03 | 4.0439654 | -2.055226 | 1.60287165 | Zc2hc1a |
| 10412667 | 0.03756 | 4.67E-03 | 4.0413618 | -2.058742 | 1.09324924 | Ptprg |
| 10445145 | 0.03763 | 4.69E-03 | -4.0381208 | -2.063121 | -1.33649268 | Olfr118 |
| 10400321 | 0.03767 | 4.71E-03 | 4.0351263 | -2.067169 | 1.08028133 | Sptssa |
| 10407327 | 0.03768 | 4.72E-03 | 4.0334743 | -2.069402 | 1.68023401 | Emb |
| 10423049 | 0.0377 | 4.73E-03 | 4.0327675 | -2.070358 | 1.01364232 | Prlr |
| 10520965 | 0.03789 | 4.77E-03 | 4.0255563 | -2.080112 | 1.05348943 | Yes1 |
| 10518361 | 0.03812 | 4.82E-03 | 4.0184571 | -2.089723 | 1.08431237 | Gm13034///Smarca5-ps///Smarca5 |
| 10371904 | 0.03815 | 4.83E-03 | 4.0168536 | -2.091895 | 1.11469689 | 1110012L19Rik |
| 10594661 | 0.03817 | 4.83E-03 | 4.0155843 | -2.093614 | 1.05385439 | Tpm1 |
| 10378802 | 0.03836 | 4.87E-03 | 4.0097428 | -2.10153 | 1.23909044 | Blmh |
| 10465980 | 0.03858 | 4.92E-03 | 4.0011845 | -2.113137 | 1.36682755 | Sdhaf2 |
| 10427428 | 0.03859 | 4.93E-03 | 4.0003688 | -2.114243 | 1.18654869 | AW549877 |
| 10502714 | 0.03859 | 4.93E-03 | 4.0003655 | -2.114248 | 1.17818341 | Rpf1 |
| 10451860 | 0.03859 | 4.93E-03 | 3.9996628 | -2.115202 | 1.05154693 | Pot1b |
| 10360522 | 0.03874 | 4.97E-03 | 3.9936211 | -2.123403 | 1.02120579 | Adss |
| 10358607 | 0.03874 | 4.97E-03 | 3.9932902 | -2.123852 | 1.07741017 | Hmcn1 |
| 10369615 | 0.03874 | 4.98E-03 | 3.9929748 | -2.124281 | 1.13822416 | Srgn |
| 10591035 | 0.03878 | 4.98E-03 | 3.9919659 | -2.125651 | 1.01504855 | Cep295 |
| 10512093 | 0.03878 | 4.99E-03 | 3.9916076 | -2.126138 | 1.58350538 | Ndufb6 |
| 10365574 | 0.03894 | 5.03E-03 | -3.9854094 | -2.134559 | -1.21839594 | Pmch |
| 10566405 | 0.03894 | 5.03E-03 | 3.9846422 | -2.135602 | 1.02713747 | Lamtor3 |
| 10389816 | 0.03913 | 5.08E-03 | 3.9775415 | -2.145257 | 1.17339839 | Tom1l1 |
| 10526452 | 0.03919 | 5.10E-03 | -3.9745204 | -2.149367 | -1.01048222 | Upk3bl |
| 10409278 | 0.03919 | 5.10E-03 | 3.9744323 | -2.149487 | 1.25664288 | Nfil3 |
| 10353754 | 0.03931 | 5.13E-03 | 3.9702696 | -2.155153 | 1.13121001 | Zfp451 |
| 10428755 | 0.03932 | 5.13E-03 | 3.9695919 | -2.156076 | 1.18827091 | Zhx1 |
| 10405781 | 0.03938 | 5.15E-03 | 3.9672468 | -2.159269 | 1.47228667 | Mir27b///2010111I01Rik |
| 10412844 | 0.03941 | 5.16E-03 | 3.9658093 | -2.161226 | 1.15587092 | Top2b |
| 10608212 | 0.03944 | 5.16E-03 | -3.9648378 | -2.16255 | -1.19295332 | Gm20871///Gm20858///Gm20857///Gm20819///Sly///Gm20736///4921509O09Rik///D630029K05Rik///1700040F15Rik///D630029K05Rik///1700040F15Rik///Pmel |
| 10494839 | 0.03945 | 5.17E-03 | 3.9641141 | -2.163536 | 1.0139939 | Csde1 |
| 10366572 | 0.03955 | 5.18E-03 | -3.962068 | -2.166323 | -2.2368772 | Iltifb///Il22 |
| 10377987 | 0.03962 | 5.20E-03 | 3.9598694 | -2.169319 | 1.04970909 | Rabep1 |
| 10402440 | 0.03968 | 5.22E-03 | -3.9558781 | -2.17476 | -1.05066656 | Gsc |
| 10564183 | 0.03972 | 5.23E-03 | -3.9544759 | -2.176672 | -1.01262402 | Snord116l2///Snord116l1///Snord116 |
| 10596043 | 0.03987 | 5.27E-03 | 3.9492802 | -2.183759 | 1.36620814 | Nck1 |
| 10590298 | 0.03987 | 5.27E-03 | 3.9482322 | -2.185189 | 1.12317765 | Eif1b |
| 10376402 | 0.03987 | 5.28E-03 | -3.9478064 | -2.18577 | -1.2687819 | Olfr322 |
| 10424746 | 0.03988 | 5.29E-03 | 3.9463831 | -2.187713 | 1.3072894 | Zfp623 |
| 10459481 | 0.03989 | 5.29E-03 | 3.9456888 | -2.18866 | 1.14062681 | Lman1 |
| 10518346 | 0.0399 | 5.29E-03 | 3.9452809 | -2.189217 | 1.3369424 | Zfp534///Zfp991///Zfp987///Zfp992///Zfp986///Znf41-ps///Zfp991 |
| 10367282 | 0.03995 | 5.30E-03 | 3.9440884 | -2.190845 | 1.1526101 | Cnpy2 |
| 10558295 | 0.03997 | 5.31E-03 | 3.9430826 | -2.192218 | 1.13340902 | Zranb1 |
| 10503168 | 0.03997 | 5.31E-03 | -3.9427435 | -2.192681 | -1.12526306 | Chd7 |
| 10419343 | 0.03999 | 5.32E-03 | 3.9416301 | -2.194201 | 1.28033948 | Atg14 |
| 10503283 | 0.04004 | 5.33E-03 | 3.9396268 | -2.196937 | 1.0565216 | 1110037F02Rik |
| 10496023 | 0.04037 | 5.41E-03 | 3.9289281 | -2.211558 | 1.79889605 | Casp6 |
| 10449991 | 0.04045 | 5.43E-03 | 3.9255674 | -2.216154 | 1.11948931 | Zfp81 |
| 10588049 | 0.04045 | 5.44E-03 | 3.924204 | -2.218019 | 1.30048376 | Copb2 |
| 10491486 | 0.0405 | 5.45E-03 | 3.9229538 | -2.21973 | 1.11001243 | Atp11b |
| 10602592 | 0.0406 | 5.47E-03 | 3.9199884 | -2.223788 | 1.27872433 | Hsd17b10 |
| 10411464 | 0.04065 | 5.48E-03 | 3.918388 | -2.225979 | 1.19224474 | Fcho2 |
| 10345546 | 0.04065 | 5.50E-03 | -3.9159448 | -2.229324 | -1.10418673 | Vwa3b |
| 10502405 | 0.04071 | 5.52E-03 | 3.9139458 | -2.232061 | 1.12282863 | Metap1 |
| 10444756 | 0.04071 | 5.52E-03 | -3.9129991 | -2.233358 | -1.00345107 | Atp6v1g2 |
| 10385036 | 0.04073 | 5.53E-03 | 3.9120608 | -2.234643 | 1.11423854 | Fgf18 |
| 10413670 | 0.04073 | 5.53E-03 | 3.9118477 | -2.234935 | 1.2608652 | Pbrm1 |
| 10498519 | 0.04087 | 5.56E-03 | 3.907608 | -2.240744 | 1.57682298 | Ssr3 |
| 10510254 | 0.04095 | 5.58E-03 | 3.9056462 | -2.243433 | 1.08413121 | Fv1 |
| 10456414 | 0.04097 | 5.58E-03 | 3.9049394 | -2.244401 | 1.01997552 | Psmg2 |
| 10508972 | 0.04102 | 5.60E-03 | -3.902668 | -2.247516 | -1.03470303 | Gm5589 |
| 10499705 | 0.04102 | 5.60E-03 | 3.9022726 | -2.248058 | 1.0364068 | Hax1 |
| 10413853 | 0.04103 | 5.61E-03 | 3.9014275 | -2.249217 | 1.04715979 | Parg |
| 10600797 | 0.04108 | 5.62E-03 | 3.8996121 | -2.251707 | 1.16205129 | Apoo-ps///Apoo |
| 10554569 | 0.0411 | 5.63E-03 | 3.8984205 | -2.253341 | 1.87105102 | Gm10767///Fam103a1///Fam103a1 |
| 10405888 | 0.0412 | 5.65E-03 | -3.89553 | -2.257307 | -1.5890303 | Cbx3 |
| 10588691 | 0.04129 | 5.67E-03 | 3.8933183 | -2.260342 | 1.02752008 | Nat6///Hyal1 |
| 10412711 | 0.04134 | 5.68E-03 | 3.8914667 | -2.262884 | 1.95760379 | Uqcrb |
| 10374415 | 0.04148 | 5.72E-03 | 3.8857242 | -2.270769 | 1.18413701 | Ppp3r1 |
| 10475211 | 0.04158 | 5.76E-03 | 3.881543 | -2.276514 | 1.37744812 | Haus2 |
| 10490291 | 0.04159 | 5.76E-03 | 3.8809153 | -2.277376 | 1.70813138 | Gm4724///Gm14410///Gm14305///Gm14308///Gm14295///0610010B08Rik///Gm14434///Gm14432///Gm14391///Gm14430///Gm6710 |
| 10433904 | 0.0416 | 5.76E-03 | 3.8803746 | -2.278119 | 1.10311328 | Yars2 |
| 10591472 | 0.04165 | 5.78E-03 | 3.8786361 | -2.280509 | 1.06490271 | Cdc37 |
| 10369704 | 0.04166 | 5.78E-03 | 3.8780333 | -2.281338 | 1.06338 | Hnrnph3///Aff3 |
| 10462100 | 0.04171 | 5.80E-03 | 3.8752651 | -2.285144 | 1.18873093 | Sarnp |
| 10541484 | 0.04171 | 5.81E-03 | 3.8749469 | -2.285581 | 1.32265108 | M6pr-ps///M6pr |
| 10503150 | 0.04188 | 5.84E-03 | 3.8700746 | -2.292283 | 1.33237461 | Rab2a |
| 10475199 | 0.0419 | 5.85E-03 | 3.8694925 | -2.293084 | 1.51377458 | Snap23 |
| 10423941 | 0.04203 | 5.88E-03 | 3.86483 | -2.299501 | 1.59945923 | Emc2 |
| 10545184 | 0.04203 | 5.88E-03 | -3.8646908 | -2.299693 | -1.38283815 | Igkv4-59///Igkv4-70///Igkc///Igkv4-59///Igkv4-80 |
| 10412549 | 0.04208 | 5.91E-03 | -3.8618775 | -2.303566 | -1.10836375 | Gm3173///Gm3739///Gm3696///Gm3667///Gm3194///Gm3558///Gm10408///Gm3500///Gm10409///Gm3383///Gm3264///Gm3636///Gm3020///Gm3002///Gm10340///Gm2897///Gm2237///Gm10406///Gm3317///2610042L04Rik///Gm5796///Gm16440///D830030K20Rik///Gm3696///Gm3667///Gm10413///Gm3264///Gm3636///Gm2897///Gm2237///Gm5795 |
| 10477370 | 0.04221 | 5.95E-03 | 3.8563102 | -2.311234 | 1.05367743 | Tomm20 |
| 10546631 | 0.04222 | 5.95E-03 | 3.8556985 | -2.312077 | 1.01028719 | Frmd4b |
| 10475405 | 0.0423 | 5.98E-03 | 3.8523625 | -2.316675 | 1.6910858 | Eif3j2///Eif3j1 |
| 10453857 | 0.04234 | 6.00E-03 | -3.8493429 | -2.320838 | -1.21186686 | Gata6 |
| 10538857 | 0.04249 | 6.05E-03 | 3.8439101 | -2.32833 | 1.29228733 | Serbp1 |
| 10382139 | 0.04256 | 6.07E-03 | 3.8417015 | -2.331378 | 1.51087695 | Psmd12 |
| 10440600 | 0.04278 | 6.13E-03 | 3.8337053 | -2.342416 | 1.46936941 | Cct8 |
| 10546452 | 0.04282 | 6.16E-03 | 3.8296547 | -2.348011 | 1.46162682 | Adamts9 |
| 10539933 | 0.04294 | 6.19E-03 | 3.8261219 | -2.352893 | 1.03222462 | Txnrd3 |
| 10601326 | 0.04311 | 6.25E-03 | 3.8184269 | -2.363532 | 1.33426729 | Uprt |
| 10608371 | 0.04312 | 6.26E-03 | -3.8180312 | -2.36408 | -2.38990885 | Gm20831///Ssty1 |
| 10582925 | 0.04321 | 6.29E-03 | 3.814379 | -2.369132 | 1.21122586 | Alkbh8 |
| 10473779 | 0.04323 | 6.29E-03 | 3.8136634 | -2.370123 | 1.01076848 | Celf1 |
| 10497817 | 0.04342 | 6.34E-03 | 3.8080216 | -2.377932 | 1.08500072 | Anxa5 |
| 10377982 | 0.04346 | 6.35E-03 | 3.8071761 | -2.379103 | 1.36336421 | Kif1c |
| 10544219 | 0.04354 | 6.37E-03 | 3.8044033 | -2.382943 | 1.10560702 | Braf |
| 10504690 | 0.04359 | 6.39E-03 | -3.8021176 | -2.38611 | -1.03139991 | Tmod1 |
| 10499198 | 0.04359 | 6.39E-03 | -3.8020212 | -2.386243 | -1.18556909 | Gm12191///Gm5481///Rpl30 |
| 10494402 | 0.04364 | 6.41E-03 | 3.799973 | -2.389081 | 1.02087887 | Hist1h3a///Hist2h3b///Hist1h3i///Hist1h3h///Hist1h3e///Hist1h3b///Hist1h3d///Hist1h3c///Hist1h3f///Hist1h3g///Hist2h3c2///Hist2h3c1///Hist1h3d |
| 10435821 | 0.04387 | 6.46E-03 | 3.7944145 | -2.396786 | 1.10053941 | Naa50 |
| 10599321 | 0.0439 | 6.48E-03 | 3.7921027 | -2.399992 | 1.03380485 | Zbtb33 |
| 10374466 | 0.04392 | 6.49E-03 | 3.79097 | -2.401563 | 1.19458062 | Rab1a |
| 10415576 | 0.04392 | 6.51E-03 | 3.7876508 | -2.406168 | 1.04190021 | Zmym2 |
| 10555785 | 0.04392 | 6.52E-03 | -3.7876083 | -2.406227 | -1.31561405 | Olfr598 |
| 10355246 | 0.04392 | 6.52E-03 | 3.7874479 | -2.406449 | 1.21065093 | Acadl |
| 10565479 | 0.04396 | 6.53E-03 | 3.7863694 | -2.407946 | 1.68794338 | l7Rn6 |
| 10536494 | 0.04411 | 6.56E-03 | 3.7824468 | -2.41339 | 1.22059542 | Cav2 |
| 10571840 | 0.04412 | 6.57E-03 | 3.7814193 | -2.414816 | 1.31941578 | Hpgd///Hpgd |
| 10395538 | 0.04415 | 6.58E-03 | 3.7802522 | -2.416437 | 1.39338707 | Pnpla8 |
| 10407916 | 0.04415 | 6.58E-03 | 3.7800022 | -2.416784 | 1.11938199 | Yae1d1 |
| 10532277 | 0.0442 | 6.60E-03 | -3.7784261 | -2.418973 | -1.19441756 | Vmn2r12///Vmn2r13 |
| 10458583 | 0.04421 | 6.60E-03 | 3.7780059 | -2.419556 | 1.10238452 | Yipf5 |
| 10350489 | 0.04442 | 6.64E-03 | 3.7730087 | -2.426498 | 1.28087601 | Uchl5 |
| 10361760 | 0.04457 | 6.68E-03 | 3.7688455 | -2.432284 | 1.63369613 | Timm8a1 |
| 10539653 | 0.04472 | 6.72E-03 | 3.7641179 | -2.438858 | 1.45507981 | Tprkb |
| 10373396 | 0.0448 | 6.74E-03 | 3.7616952 | -2.442228 | 1.0568397 | Myl6///Myl6 |
| 10586759 | 0.04484 | 6.75E-03 | 3.7604647 | -2.443939 | 1.01370258 | Bnip2 |
| 10425757 | 0.04495 | 6.78E-03 | 3.7571513 | -2.44855 | 1.14298052 | Smdt1///Smdt1 |
| 10384349 | 0.04501 | 6.82E-03 | 3.7535923 | -2.453504 | 1.01171637 | Polr2c |
| 10547521 | 0.04501 | 6.82E-03 | 3.7533244 | -2.453877 | 1.27945894 | Atp6v1e1 |
| 10559820 | 0.04513 | 6.87E-03 | 3.7474571 | -2.462048 | 1.10473249 | Zfp954 |
| 10601616 | 0.04517 | 6.89E-03 | 3.746001 | -2.464077 | 1.00851078 | Diaph2 |
| 10512279 | 0.04517 | 6.89E-03 | 3.7459327 | -2.464172 | 1.22297325 | Cntfr |
| 10403816 | 0.04529 | 6.92E-03 | -3.7422469 | -2.469308 | -1.21218997 | Tcrg-V6///Tcrg-V4 |
| 10348932 | 0.0453 | 6.93E-03 | 3.7416463 | -2.470145 | 1.03093607 | Gin1 |
| 10421840 | 0.04579 | 7.07E-03 | 3.7266568 | -2.491054 | 1.13511988 | Wbp4 |
| 10580391 | 0.04592 | 7.11E-03 | 3.7222089 | -2.497264 | 1.43052538 | Itfg1 |
| 10500614 | 0.04592 | 7.11E-03 | 3.7221202 | -2.497388 | 1.2170489 | Man1a2 |
| 10559883 | 0.04592 | 7.11E-03 | 3.7220901 | -2.49743 | 1.25559274 | Vmn2r51///Vmn2r36///Vmn2r34///Vmn2r28///Vmn2r48///Vmn2r35///Vmn2r33///Vmn2r43///Vmn2r29///Vmn2r32///Vmn2r42///Vmn2r30///Vmn2r37 |
| 10439642 | 0.04595 | 7.13E-03 | 3.7197264 | -2.500731 | 1.11224856 | Slc35a5 |
| 10483648 | 0.046 | 7.15E-03 | 3.7181565 | -2.502924 | 1.11349543 | Ola1 |
| 10454966 | 0.04607 | 7.17E-03 | 3.7156925 | -2.506367 | 1.5254671 | Ik |
| 10529801 | 0.04625 | 7.23E-03 | 3.7092967 | -2.515307 | 1.07354952 | Fbxl5 |
| 10607467 | 0.04654 | 7.32E-03 | 3.7006528 | -2.527399 | 1.19592423 | Sat1 |
| 10494662 | 0.04663 | 7.34E-03 | 3.6982203 | -2.530804 | 1.44092584 | Ywhah |
| 10498386 | 0.04664 | 7.34E-03 | 3.6977541 | -2.531456 | 1.27634595 | Igsf10 |
| 10455738 | 0.04697 | 7.43E-03 | 3.6887802 | -2.544024 | 1.39101159 | Snx2 |
| 10542200 | 0.04724 | 7.51E-03 | 3.6811749 | -2.554683 | 1.2140217 | Gabarapl1 |
| 10356001 | 0.04728 | 7.52E-03 | 3.6803209 | -2.555881 | 1.64796241 | Cul3 |
| 10424485 | 0.04744 | 7.58E-03 | 3.6738053 | -2.565019 | 1.1699912 | Phf20l1 |
| 10528691 | 0.04746 | 7.59E-03 | 3.6731074 | -2.565999 | 2.00389827 | Rheb |
| 10551815 | 0.04753 | 7.63E-03 | 3.6694664 | -2.571108 | 1.12114569 | Zfp260 |
| 10436442 | 0.04754 | 7.64E-03 | 3.6684115 | -2.572589 | 1.51131387 | Fam60a |
| 10571005 | 0.04755 | 7.65E-03 | -3.6675625 | -2.573781 | -1.03205511 | D830025C05Rik |
| 10432897 | 0.04779 | 7.74E-03 | -3.658625 | -2.586334 | -1.14024171 | Krt79 |
| 10344966 | 0.04787 | 7.77E-03 | 3.6561942 | -2.58975 | 1.69315583 | Ly96 |
| 10530759 | 0.04791 | 7.79E-03 | 3.6543943 | -2.592279 | 1.13063502 | Ube2n |
| 10468131 | 0.04794 | 7.80E-03 | 3.6534434 | -2.593616 | 1.04111693 | 9130011E15Rik |
| 10448004 | 0.04795 | 7.80E-03 | 3.6528453 | -2.594457 | 1.55589482 | Phf10 |
| 10569890 | 0.04796 | 7.81E-03 | 3.6523365 | -2.595172 | 1.32793311 | Aida |
| 10350478 | 0.04806 | 7.84E-03 | 3.6487634 | -2.600197 | 1.32258689 | Glrx2///Glrx2 |
| 10517421 | 0.04806 | 7.85E-03 | 3.6486497 | -2.600357 | 1.25351528 | Pnrc2 |
| 10499080 | 0.04806 | 7.85E-03 | 3.6483613 | -2.600762 | 1.20851811 | Arfip1 |
| 10394102 | 0.04806 | 7.85E-03 | 3.6480244 | -2.601236 | 1.23031665 | Wdr45b |
| 10475394 | 0.04809 | 7.86E-03 | 3.6472148 | -2.602375 | 1.32565167 | Ctdspl2 |
| 10365482 | 0.04809 | 7.86E-03 | 3.647031 | -2.602633 | 1.19326492 | Timp3 |
| 10365974 | 0.04816 | 7.90E-03 | -3.6435185 | -2.607575 | -1.07519186 | Dcn |
| 10495651 | 0.04825 | 7.94E-03 | 3.6398666 | -2.612714 | 1.73921352 | Alg14 |
| 10543052 | 0.04825 | 7.94E-03 | -3.6395614 | -2.613144 | -1.08731287 | Rps27a |
| 10567173 | 0.04834 | 7.97E-03 | 3.6371499 | -2.616539 | 1.2760134 | Pik3c2a |
| 10384504 | 0.04839 | 7.98E-03 | 3.6356166 | -2.618698 | 1.05091896 | Meis1 |
| 10362922 | 0.04849 | 8.01E-03 | 3.6328432 | -2.622604 | 1.04978326 | Atg5 |
| 10432780 | 0.04868 | 8.08E-03 | 3.626794 | -2.631126 | 1.24616283 | Krt6a |
| 10445147 | 0.04882 | 8.15E-03 | -3.6203841 | -2.640162 | -1.16541864 | Olfr119 |
| 10350592 | 0.04891 | 8.17E-03 | -3.6183702 | -2.643003 | -1.21035991 | 3110040M04Rik |
| 10388684 | 0.04895 | 8.19E-03 | 3.6171661 | -2.644701 | 1.15965129 | Taok1 |
| 10513952 | 0.04896 | 8.19E-03 | 3.6169732 | -2.644973 | 1.22766242 | Tmem261 |
| 10487175 | 0.04902 | 8.20E-03 | 3.615554 | -2.646975 | 1.32884707 | Cops2 |
| 10461991 | 0.04907 | 8.23E-03 | 3.6135453 | -2.649809 | 1.06115618 | Zfand5 |
| 10490250 | 0.04907 | 8.23E-03 | 3.6133771 | -2.650046 | 1.00761034 | Gm14296///2210418O10Rik///Gm4724///Gm14410///Gm14305///Gm14308///Gm14295///0610010B08Rik///Gm14434///Gm14432///Gm14326///Gm14391///Zfp970///Gm14430///Zfp971///Gm6710///Gm14403///Zfp931///Gm14325 |
| 10502232 | 0.04907 | 8.23E-03 | 3.6132558 | -2.650218 | 1.06775984 | Aimp1 |
| 10444312 | 0.04911 | 8.24E-03 | -3.6123462 | -2.651501 | -1.12465534 | Btnl2 |
| 10399874 | 0.04917 | 8.26E-03 | 3.6101174 | -2.654647 | 1.15263941 | Bcap29 |
| 10471978 | 0.04928 | 8.31E-03 | 3.6056647 | -2.660933 | 1.11025267 | Epc2 |
| 10587284 | 0.04932 | 8.33E-03 | 3.6042169 | -2.662977 | 1.06753827 | Elovl5 |
| 10488124 | 0.04933 | 8.34E-03 | -3.6038785 | -2.663455 | -1.0050025 | Sel1l2 |
| 10485656 | 0.04943 | 8.36E-03 | 3.6015166 | -2.666791 | 1.23852588 | Elp4 |
| 10596269 | 0.04975 | 8.45E-03 | 3.5933982 | -2.678264 | 1.1206901 | Dnajc13 |
| 10406459 | 0.04983 | 8.49E-03 | 3.5906903 | -2.682092 | 1.07488672 | Ndufc1 |
| 10458349 | 0.04983 | 8.49E-03 | 3.5906852 | -2.682099 | 1.03394729 | Sra1 |

**Table S2 GSE117168 Full length DEGs**

| **ID** | **adj.P.Val** | **P.Value** | **t** | **B** | **logFC** | **Gene.symbol** |
| --- | --- | --- | --- | --- | --- | --- |
| 1450871_PM_a_at | 7.61E-08 | 1.69E-12 | 4.40E+01 | 16.505925 | 5.38 | Bcat1 |
| 1460129_PM_at | 8.55E-07 | 8.89E-11 | 2.92E+01 | 14.299212 | 2.55 | Slc6a2 |
| 1448237_PM_x_at | 8.55E-07 | 1.17E-10 | -2.83E+01 | 14.11529 | -2.45 | Ldhb |
| 1424528_PM_at | 8.55E-07 | 1.23E-10 | 2.82E+01 | 14.079795 | 3.31 | Cgref1 |
| 1442449_PM_at | 8.55E-07 | 1.32E-10 | 2.80E+01 | 14.031924 | 2.53 | Slc6a2 |
| 1439622_PM_at | 8.55E-07 | 1.33E-10 | 2.80E+01 | 14.02888 | 4.1 | Rassf4 |
| 1424688_PM_at | 1.18E-06 | 2.10E-10 | 2.67E+01 | 13.706484 | 3.6 | Creb3l3 |
| 1436119_PM_at | 1.26E-06 | 2.51E-10 | 2.62E+01 | 13.578111 | 3.71 | Aldh1l2 |
| 1439816_PM_at | 1.62E-06 | 3.58E-10 | -2.52E+01 | 13.31944 | -2.65 | Tcf24 |
| 1424529_PM_s_at | 2.35E-06 | 7.25E-10 | 2.34E+01 | 12.784771 | 3.24 | Cgref1 |
| 1418004_PM_a_at | 2.35E-06 | 7.28E-10 | -2.34E+01 | 12.782382 | -2.25 | Tmem176b |
| 1416183_PM_a_at | 4.46E-06 | 1.78E-09 | -2.13E+01 | 12.071965 | -2.43 | Ldhb |
| 1450276_PM_a_at | 5.38E-06 | 2.32E-09 | -2.07E+01 | 11.853315 | -1.83 | Scin |
| 1421282_PM_at | 5.38E-06 | 2.38E-09 | 2.07E+01 | 11.831906 | 1.77 | Bmp5 |
| 1448280_PM_at | 6.24E-06 | 2.96E-09 | -2.02E+01 | 11.65266 | -1.61 | Syp |
| 1433626_PM_at | 6.24E-06 | 3.16E-09 | -2.01E+01 | 11.598046 | -2.6 | Plscr4 |
| 1452183_PM_a_at | 6.24E-06 | 3.18E-09 | 2.01E+01 | 11.591608 | 1.81 | Meg3 |
| 1447311_PM_at | 6.49E-06 | 3.60E-09 | 1.98E+01 | 11.488642 | 2.33 | Slc6a2 |
| 1455699_PM_at | 6.59E-06 | 3.81E-09 | 1.97E+01 | 11.440677 | 3.15 | Bcat1 |
| 1452905_PM_at | 6.59E-06 | 3.94E-09 | 1.96E+01 | 11.411154 | 1.64 | Meg3 |
| 1427167_PM_at | 7.20E-06 | 4.53E-09 | 1.93E+01 | 11.293106 | 1.98 | Armcx4///DXErtd573e |
| 1429256_PM_at | 7.20E-06 | 4.78E-09 | 1.92E+01 | 11.246261 | 1.81 | Meg3 |
| 1421641_PM_at | 7.31E-06 | 5.03E-09 | 1.91E+01 | 11.202623 | 1.99 | Slc6a2 |
| 1434291_PM_a_at | 7.31E-06 | 5.18E-09 | 1.90E+01 | 11.177362 | 1.44 | Serf1 |
| 1430712_PM_at | 8.44E-06 | 6.36E-09 | 1.86E+01 | 11.001182 | 1.9 | Arhgap24 |
| 1416086_PM_at | 8.47E-06 | 6.70E-09 | 1.85E+01 | 10.956376 | 1.17 | Tpst2 |
| 1417355_PM_at | 8.47E-06 | 6.95E-09 | 1.85E+01 | 10.924573 | 1.4 | Peg3 |
| 1451611_PM_at | 8.47E-06 | 7.07E-09 | -1.84E+01 | 10.909351 | -1.47 | Pla2g16 |
| 1434580_PM_at | 8.47E-06 | 7.13E-09 | -1.84E+01 | 10.901298 | -1.62 | Enpp4 |
| 1452906_PM_at | 8.61E-06 | 7.44E-09 | 1.83E+01 | 10.864598 | 1.68 | Meg3 |
| 1459911_PM_at | 9.09E-06 | 8.05E-09 | 1.82E+01 | 10.795762 | 1.07 | Cdr2l |
| 1435036_PM_at | 9.34E-06 | 8.52E-09 | 1.81E+01 | 10.746125 | 1.33 | Aspg |
| 1426758_PM_s_at | 9.34E-06 | 8.69E-09 | 1.80E+01 | 10.729005 | 1.83 | Meg3 |
| 1435990_PM_at | 9.77E-06 | 9.60E-09 | 1.78E+01 | 10.64174 | 1.16 | Adamts2 |
| 1423593_PM_a_at | 9.77E-06 | 9.86E-09 | 1.78E+01 | 10.617502 | 1.92 | Csf1r |
| 1451886_PM_at | 9.77E-06 | 1.01E-08 | 1.78E+01 | 10.599109 | 1.03 | Speg |
| 1452453_PM_a_at | 1.01E-05 | 1.09E-08 | 1.76E+01 | 10.528021 | 2.55 | Camk2a |
| 1441811_PM_x_at | 1.01E-05 | 1.10E-08 | -1.76E+01 | 10.524314 | -2.62 | Tmem176a |
| 1424556_PM_at | 1.02E-05 | 1.13E-08 | 1.75E+01 | 10.495342 | 2.68 | Pycr1 |
| 1422866_PM_at | 1.07E-05 | 1.22E-08 | 1.74E+01 | 10.425657 | 1.58 | Col13a1 |
| 1427580_PM_a_at | 1.07E-05 | 1.23E-08 | 1.74E+01 | 10.420096 | 2.74 | Rian |
| 1448152_PM_at | 1.07E-05 | 1.28E-08 | -1.73E+01 | 10.383874 | -2.37 | Igf2 |
| 1430111_PM_a_at | 1.08E-05 | 1.31E-08 | 1.73E+01 | 10.3649 | 2.21 | Bcat1 |
| 1437568_PM_at | 1.15E-05 | 1.43E-08 | 1.71E+01 | 10.285941 | 1.28 | Mmp16 |
| 1443412_PM_s_at | 1.16E-05 | 1.47E-08 | 1.71E+01 | 10.264053 | 1.74 | Mmp16 |
| 1425968_PM_s_at | 1.16E-05 | 1.48E-08 | 1.70E+01 | 10.253764 | 1.16 | Speg |
| 1449509_PM_at | 1.45E-05 | 1.96E-08 | 1.65E+01 | 10.004155 | 1.43 | Serf1 |
| 1448929_PM_at | 1.52E-05 | 2.12E-08 | 1.64E+01 | 9.934317 | 1.72 | F13a1 |
| 1449581_PM_at | 1.74E-05 | 2.47E-08 | 1.61E+01 | 9.793269 | 1.58 | Emid1 |
| 1434069_PM_at | 1.75E-05 | 2.53E-08 | 1.61E+01 | 9.773303 | 1.24 | Prex1 |
| 1417149_PM_at | 2.04E-05 | 2.98E-08 | 1.58E+01 | 9.622186 | 1.18 | P4ha2 |
| 1427884_PM_at | 2.06E-05 | 3.07E-08 | 1.58E+01 | 9.594727 | 1.86 | Col3a1 |
| 1427294_PM_a_at | 2.06E-05 | 3.10E-08 | 1.58E+01 | 9.58455 | 1.27 | Slc38a10 |
| 1428764_PM_at | 2.09E-05 | 3.20E-08 | 1.57E+01 | 9.556867 | 1.67 | Meg3 |
| 1429027_PM_at | 2.11E-05 | 3.28E-08 | 1.57E+01 | 9.533651 | 1.05 | Snhg18 |
| 1436057_PM_at | 2.13E-05 | 3.39E-08 | 1.56E+01 | 9.504264 | 1.11 | Meg3 |
| 1448788_PM_at | 2.13E-05 | 3.39E-08 | -1.56E+01 | 9.502195 | -1.94 | Cd200 |
| 1456017_PM_x_at | 2.39E-05 | 3.97E-08 | 1.53E+01 | 9.357987 | 2.22 | Obox2 |
| 1415931_PM_at | 2.42E-05 | 4.13E-08 | -1.53E+01 | 9.320491 | -2.61 | Igf2 |
| 1450344_PM_a_at | 2.57E-05 | 4.59E-08 | -1.51E+01 | 9.221444 | -1.97 | Ptger3 |
| 1416515_PM_at | 2.57E-05 | 4.65E-08 | 1.51E+01 | 9.210014 | 1.8 | Fscn1 |
| 1429399_PM_at | 2.57E-05 | 4.67E-08 | -1.51E+01 | 9.20679 | -1.12 | Rnf125 |
| 1437125_PM_at | 2.57E-05 | 4.68E-08 | 1.51E+01 | 9.205186 | 3.04 | Camk2a |
| 1416741_PM_at | 2.58E-05 | 4.74E-08 | 1.51E+01 | 9.193305 | 1.36 | Col5a1 |
| 1416289_PM_at | 2.76E-05 | 5.14E-08 | 1.49E+01 | 9.116996 | 1.41 | Plod1 |
| 1449270_PM_at | 2.88E-05 | 5.50E-08 | -1.48E+01 | 9.053754 | -1.6 | Plxdc2 |
| 1416805_PM_at | 2.88E-05 | 5.53E-08 | 1.48E+01 | 9.049021 | 1.76 | Fam198b |
| 1421283_PM_at | 2.88E-05 | 5.62E-08 | 1.48E+01 | 9.033139 | 1.25 | Bmp5 |
| 1431094_PM_at | 2.89E-05 | 5.77E-08 | 1.47E+01 | 9.008311 | 1.8 | 1110006E14Rik |
| 1433877_PM_at | 2.90E-05 | 5.85E-08 | -1.47E+01 | 8.995194 | -2.33 | Fam46b |
| 1428168_PM_at | 3.40E-05 | 7.01E-08 | 1.44E+01 | 8.825259 | 1.58 | Mpzl1 |
| 1458833_PM_at | 3.53E-05 | 7.59E-08 | 1.43E+01 | 8.750234 | 1.87 | Nrcam |
| 1418086_PM_at | 3.61E-05 | 7.83E-08 | 1.43E+01 | 8.721054 | 1.22 | Ppp1r14a |
| 1418538_PM_at | 3.62E-05 | 7.94E-08 | 1.42E+01 | 8.707697 | 1.15 | Kdelr3 |
| 1429637_PM_at | 3.70E-05 | 8.29E-08 | 1.42E+01 | 8.66753 | 2.13 | Fam198b |
| 1431569_PM_a_at | 3.95E-05 | 9.01E-08 | 1.41E+01 | 8.587667 | 1.35 | Lypd1 |
| 1449440_PM_at | 3.95E-05 | 9.11E-08 | 1.40E+01 | 8.577834 | 1.11 | Lpin3 |
| 1428167_PM_a_at | 4.11E-05 | 9.92E-08 | 1.39E+01 | 8.496967 | 1.73 | Mpzl1 |
| 1429979_PM_a_at | 4.22E-05 | 1.03E-07 | 1.38E+01 | 8.45637 | 1.14 | Slc38a10 |
| 1436713_PM_s_at | 4.22E-05 | 1.04E-07 | 1.38E+01 | 8.453788 | 2.41 | Meg3 |
| 1417836_PM_at | 4.39E-05 | 1.10E-07 | 1.37E+01 | 8.395725 | 1.02 | Gpx7 |
| 1418796_PM_at | 4.39E-05 | 1.11E-07 | 1.37E+01 | 8.388355 | 1.69 | Clec11a |
| 1423239_PM_at | 4.39E-05 | 1.12E-07 | 1.37E+01 | 8.381393 | 1.21 | Impdh1 |
| 1422851_PM_at | 4.67E-05 | 1.22E-07 | -1.36E+01 | 8.299171 | -1.21 | Hmga2 |
| 1456624_PM_at | 4.82E-05 | 1.28E-07 | 1.35E+01 | 8.25612 | 1.27 | Wipi1 |
| 1434920_PM_a_at | 5.23E-05 | 1.45E-07 | 1.34E+01 | 8.134821 | 1.27 | Evl |
| 1454023_PM_a_at | 5.52E-05 | 1.57E-07 | 1.32E+01 | 8.054695 | 1.16 | Chpf |
| 1458667_PM_at | 5.52E-05 | 1.58E-07 | -1.32E+01 | 8.051925 | -1.85 | Ninl |
| 1436346_PM_at | 6.22E-05 | 1.81E-07 | -1.30E+01 | 7.921639 | -1.22 | Cd109 |
| 1425475_PM_at | 6.22E-05 | 1.82E-07 | 1.30E+01 | 7.915825 | 2.51 | Col4a5 |
| 1448510_PM_at | 6.47E-05 | 1.94E-07 | 1.29E+01 | 7.854834 | 1.3 | Efna1 |
| 1438530_PM_at | 7.38E-05 | 2.26E-07 | -1.27E+01 | 7.706312 | -1.23 | Tfpi |
| 1432448_PM_at | 7.60E-05 | 2.34E-07 | 1.27E+01 | 7.671163 | 1.19 | 2600006K01Rik |
| 1423909_PM_at | 7.78E-05 | 2.43E-07 | -1.26E+01 | 7.63405 | -2.58 | Tmem176a |
| 1416121_PM_at | 8.02E-05 | 2.54E-07 | 1.26E+01 | 7.591461 | 1.4 | Lox |
| 1434089_PM_at | 8.13E-05 | 2.59E-07 | -1.25E+01 | 7.571391 | -1.6 | Synpo |
| 1420690_PM_at | 8.25E-05 | 2.67E-07 | -1.25E+01 | 7.543595 | -1.28 | Fgf10 |
| 1450781_PM_at | 8.32E-05 | 2.71E-07 | -1.25E+01 | 7.528031 | -2.06 | Hmga2 |
| 1425658_PM_at | 8.71E-05 | 2.90E-07 | -1.24E+01 | 7.463746 | -1.06 | Cd109 |
| 1421061_PM_at | 9.00E-05 | 3.02E-07 | 1.23E+01 | 7.42242 | 1.19 | Guca1a |
| 1427295_PM_at | 9.00E-05 | 3.03E-07 | 1.23E+01 | 7.417844 | 1.3 | Slc38a10 |
| 1435261_PM_at | 9.28E-05 | 3.17E-07 | -1.23E+01 | 7.376 | -1.34 | Tmtc1///4732416N19Rik |
| 1428455_PM_at | 9.33E-05 | 3.20E-07 | 1.22E+01 | 7.364369 | 3.35 | Col14a1 |
| 1424245_PM_at | 9.34E-05 | 3.25E-07 | 1.22E+01 | 7.351232 | 1.71 | Ces2c |
| 1455851_PM_at | 9.49E-05 | 3.34E-07 | 1.22E+01 | 7.323033 | 1.64 | Bmp5 |
| 1436337_PM_at | 9.52E-05 | 3.39E-07 | -1.22E+01 | 7.310806 | -1.01 | Tmem243///Gm17739 |
| 1445027_PM_at | 9.52E-05 | 3.40E-07 | 1.22E+01 | 7.307607 | 1.31 | Cdr2l |
| 1460253_PM_at | 9.84E-05 | 3.60E-07 | 1.21E+01 | 7.25154 | 1.01 | Cmtm7 |
| 1415829_PM_at | 9.93E-05 | 3.67E-07 | -1.21E+01 | 7.23102 | -1.18 | Lbr |
| 1451866_PM_a_at | 1.03E-04 | 3.85E-07 | -1.20E+01 | 7.183978 | -1.03 | Hgf |
| 1439069_PM_a_at | 1.03E-04 | 3.86E-07 | -1.20E+01 | 7.182407 | -1.03 | Pisd-ps1///Pisd-ps3 |
| 1416203_PM_at | 1.04E-04 | 3.93E-07 | -1.20E+01 | 7.164706 | -2.75 | Aqp1 |
| 1452639_PM_at | 1.05E-04 | 3.98E-07 | -1.20E+01 | 7.152944 | -1.26 | Enpp4 |
| 1433935_PM_at | 1.07E-04 | 4.11E-07 | -1.19E+01 | 7.119823 | -1.05 | AU020206 |
| 1424470_PM_a_at | 1.07E-04 | 4.13E-07 | -1.19E+01 | 7.114841 | -1.25 | Rapgef3 |
| 1452392_PM_a_at | 1.14E-04 | 4.67E-07 | 1.18E+01 | 6.994683 | 1.03 | Wipi1 |
| 1456635_PM_at | 1.14E-04 | 4.69E-07 | -1.17E+01 | 6.990768 | -1.17 | A530040E14Rik |
| 1450571_PM_a_at | 1.15E-04 | 4.76E-07 | 1.17E+01 | 6.975716 | 1.05 | Bfsp1 |
| 1460603_PM_at | 1.23E-04 | 5.27E-07 | -1.16E+01 | 6.874379 | -1.28 | Samd9l |
| 1425476_PM_at | 1.23E-04 | 5.31E-07 | 1.16E+01 | 6.868296 | 1.84 | Col4a5 |
| 1418080_PM_at | 1.26E-04 | 5.48E-07 | 1.15E+01 | 6.836906 | 1.04 | B4galt2 |
| 1416753_PM_at | 1.29E-04 | 5.68E-07 | -1.15E+01 | 6.801427 | -1.05 | Prkar1b |
| 1419254_PM_at | 1.29E-04 | 5.72E-07 | 1.15E+01 | 6.794135 | 2.03 | Gsn///Mthfd2 |
| 1435275_PM_at | 1.29E-04 | 5.75E-07 | -1.15E+01 | 6.789409 | -1.33 | Cox6b2 |
| 1415951_PM_at | 1.29E-04 | 5.81E-07 | 1.15E+01 | 6.779471 | 1.36 | Fkbp10 |
| 1436528_PM_at | 1.35E-04 | 6.20E-07 | 1.14E+01 | 6.713858 | 1.78 | Kazald1 |
| 1429257_PM_at | 1.35E-04 | 6.34E-07 | 1.14E+01 | 6.692854 | 1.52 | Meg3 |
| 1424581_PM_at | 1.39E-04 | 6.63E-07 | -1.13E+01 | 6.647876 | -1.43 | Stac2 |
| 1459622_PM_at | 1.39E-04 | 6.69E-07 | 1.13E+01 | 6.639625 | 1.34 | Gm22 |
| 1426670_PM_at | 1.39E-04 | 6.71E-07 | -1.13E+01 | 6.635667 | -1.14 | Agrn |
| 1422397_PM_a_at | 1.39E-04 | 6.73E-07 | -1.13E+01 | 6.632697 | -1.06 | Il15ra |
| 1416644_PM_a_at | 1.42E-04 | 6.91E-07 | 1.13E+01 | 6.606117 | 1.08 | Sema3b |
| 1452899_PM_at | 1.45E-04 | 7.18E-07 | 1.12E+01 | 6.568284 | 2.55 | Rian |
| 1436861_PM_at | 1.47E-04 | 7.32E-07 | -1.12E+01 | 6.549763 | -1.08 | Il7 |
| 1450625_PM_at | 1.52E-04 | 7.59E-07 | 1.11E+01 | 6.513048 | 1.77 | Col5a2 |
| 1419613_PM_at | 1.54E-04 | 7.80E-07 | 1.11E+01 | 6.485897 | 1 | Col7a1 |
| 1431362_PM_a_at | 1.56E-04 | 7.94E-07 | 1.11E+01 | 6.46896 | 1.03 | Smoc2 |
| 1428572_PM_at | 1.66E-04 | 8.53E-07 | 1.10E+01 | 6.396826 | 1.54 | Basp1 |
| 1415836_PM_at | 1.72E-04 | 9.11E-07 | 1.09E+01 | 6.331438 | 1.04 | Aldh18a1 |
| 1435792_PM_at | 1.72E-04 | 9.11E-07 | -1.09E+01 | 6.331266 | -2.93 | Csprs |
| 1418099_PM_at | 1.72E-04 | 9.12E-07 | -1.09E+01 | 6.33044 | -1.06 | Tnfrsf1b |
| 1422141_PM_s_at | 1.72E-04 | 9.14E-07 | -1.09E+01 | 6.328115 | -2.35 | Csprs |
| 1425251_PM_at | 1.79E-04 | 9.88E-07 | -1.08E+01 | 6.251055 | -1.21 | Ptger3 |
| 1427919_PM_at | 1.79E-04 | 9.89E-07 | -1.08E+01 | 6.249494 | -1.11 | Srpx2 |
| 1432062_PM_at | 1.83E-04 | 1.02E-06 | 1.08E+01 | 6.219223 | 1.1 | Mdga1 |
| 1423668_PM_at | 1.85E-04 | 1.03E-06 | 1.08E+01 | 6.207977 | 1.08 | Zdhhc14 |
| 1455145_PM_at | 2.00E-04 | 1.15E-06 | -1.06E+01 | 6.096152 | -1.29 | Pcdh19 |
| 1417356_PM_at | 2.05E-04 | 1.21E-06 | 1.06E+01 | 6.047842 | 1.66 | Peg3 |
| 1427168_PM_a_at | 2.05E-04 | 1.21E-06 | 1.06E+01 | 6.047645 | 2.88 | Col14a1 |
| 1425741_PM_at | 2.05E-04 | 1.22E-06 | 1.06E+01 | 6.040546 | 1.19 | Srgap3 |
| 1425536_PM_at | 2.11E-04 | 1.27E-06 | -1.05E+01 | 6.002447 | -1.38 | Stx3 |
| 1421497_PM_at | 2.12E-04 | 1.30E-06 | 1.05E+01 | 5.976796 | 1.43 | Gpha2 |
| 1455280_PM_at | 2.16E-04 | 1.33E-06 | 1.05E+01 | 5.950734 | 1.99 | Frem1 |
| 1419253_PM_at | 2.17E-04 | 1.34E-06 | 1.05E+01 | 5.946618 | 1.5 | Gsn///Mthfd2 |
| 1454890_PM_at | 2.20E-04 | 1.37E-06 | -1.04E+01 | 5.921784 | -1.19 | Amot |
| 1421462_PM_a_at | 2.31E-04 | 1.47E-06 | 1.04E+01 | 5.851203 | 1.09 | P3h1 |
| 1437629_PM_at | 2.31E-04 | 1.48E-06 | 1.03E+01 | 5.846031 | 1.13 | Arhgef19 |
| 1445359_PM_at | 2.31E-04 | 1.48E-06 | 1.03E+01 | 5.844434 | 1.91 | Adcy1 |
| 1439373_PM_x_at | 2.32E-04 | 1.50E-06 | -1.03E+01 | 5.833713 | -1.14 | Wnt5b |
| 1434975_PM_x_at | 2.38E-04 | 1.57E-06 | -1.03E+01 | 5.787412 | -1.16 | Pisd-ps1///Pisd-ps3 |
| 1428055_PM_at | 2.40E-04 | 1.60E-06 | 1.03E+01 | 5.766914 | 1.61 | Rian |
| 1457311_PM_at | 2.50E-04 | 1.70E-06 | 1.02E+01 | 5.708934 | 2.53 | Camk2a |
| 1424677_PM_at | 2.52E-04 | 1.72E-06 | -1.02E+01 | 5.69319 | -1.62 | Cyp2j9 |
| 1448620_PM_at | 2.56E-04 | 1.76E-06 | 1.02E+01 | 5.673075 | 1.32 | Fcgr3 |
| 1423319_PM_at | 2.56E-04 | 1.77E-06 | -1.01E+01 | 5.662888 | -1.13 | Hhex |
| 1448996_PM_at | 2.64E-04 | 1.85E-06 | 1.01E+01 | 5.622591 | 1.57 | Rom1 |
| 1449172_PM_a_at | 2.67E-04 | 1.90E-06 | 1.01E+01 | 5.594002 | 1.1 | Lin7b |
| 1418162_PM_at | 2.68E-04 | 1.93E-06 | -1.00E+01 | 5.575477 | -1.28 | Tlr4 |
| 1436055_PM_at | 2.71E-04 | 1.97E-06 | -1.00E+01 | 5.559036 | -1.53 | Lrrc15 |
| 1424923_PM_at | 2.85E-04 | 2.14E-06 | -9.93 | 5.474498 | -1.7 | Serpina3g |
| 1457799_PM_at | 2.88E-04 | 2.19E-06 | -9.9 | 5.449388 | -1.2 | Kcns3 |
| 1418912_PM_at | 2.89E-04 | 2.20E-06 | -9.9 | 5.445452 | -1.07 | Plxdc2 |
| 1424650_PM_at | 2.92E-04 | 2.24E-06 | 9.88 | 5.427767 | 1.29 | Pdia5 |
| 1450852_PM_s_at | 2.97E-04 | 2.31E-06 | -9.84 | 5.397639 | -1.48 | F2r |
| 1418566_PM_s_at | 2.97E-04 | 2.31E-06 | 9.84 | 5.396314 | 1.06 | Nudcd2 |
| 1458903_PM_at | 3.01E-04 | 2.36E-06 | 9.82 | 5.375163 | 1.02 | Miat |
| 1426147_PM_s_at | 3.01E-04 | 2.36E-06 | 9.82 | 5.374116 | 1.05 | Cldn10 |
| 1456487_PM_at | 3.03E-04 | 2.38E-06 | 9.81 | 5.365464 | 1.97 | Adcy1 |
| 1417348_PM_at | 3.03E-04 | 2.39E-06 | 9.8 | 5.361168 | 1.01 | 2310039H08Rik |
| 1446791_PM_at | 3.15E-04 | 2.54E-06 | 9.74 | 5.30094 | 1.02 | Pi15 |
| 1438231_PM_at | 3.17E-04 | 2.56E-06 | -9.73 | 5.292537 | -1.02 | Foxp2 |
| 1450027_PM_at | 3.19E-04 | 2.59E-06 | 9.72 | 5.280452 | 1.37 | Sdc3 |
| 1423310_PM_at | 3.21E-04 | 2.62E-06 | -9.71 | 5.26959 | -1.7 | Tpbg |
| 1419358_PM_at | 3.21E-04 | 2.64E-06 | 9.7 | 5.260849 | 1.11 | Sorcs2 |
| 1421346_PM_a_at | 3.22E-04 | 2.66E-06 | -9.69 | 5.253347 | -2.03 | Slc6a6 |
| 1439117_PM_at | 3.27E-04 | 2.72E-06 | 9.66 | 5.229674 | 1.09 | Clmn |
| 1423055_PM_at | 3.27E-04 | 2.73E-06 | -9.66 | 5.228501 | -1.04 | Nsg1 |
| 1439830_PM_at | 3.33E-04 | 2.81E-06 | -9.63 | 5.198509 | -1.12 | Map3k5 |
| 1449286_PM_at | 3.34E-04 | 2.85E-06 | -9.61 | 5.181764 | -1.4 | Ntng1 |
| 1435084_PM_at | 3.41E-04 | 2.96E-06 | -9.57 | 5.146324 | -1.15 | C730049O14Rik |
| 1417697_PM_at | 3.42E-04 | 2.97E-06 | -9.57 | 5.140026 | -1.18 | Soat1 |
| 1444009_PM_at | 3.46E-04 | 3.03E-06 | 9.55 | 5.121165 | 1.67 | Rassf4 |
| 1456515_PM_s_at | 3.64E-04 | 3.24E-06 | 9.48 | 5.054162 | 1.83 | Tcfl5 |
| 1429235_PM_at | 3.72E-04 | 3.35E-06 | -9.44 | 5.020103 | -1.9 | Galnt15 |
| 1427508_PM_at | 3.72E-04 | 3.36E-06 | 9.44 | 5.016435 | 1.2 | Arsi |
| 1416051_PM_at | 3.72E-04 | 3.37E-06 | -9.43 | 5.014359 | -1.24 | C2 |
| 1448529_PM_at | 3.72E-04 | 3.37E-06 | -9.43 | 5.013446 | -1.32 | Thbd |
| 1440132_PM_s_at | 3.81E-04 | 3.50E-06 | -9.39 | 4.973582 | -1.29 | Prkar1b |
| 1431110_PM_at | 3.83E-04 | 3.53E-06 | -9.38 | 4.966939 | -1.33 | Plxdc2 |
| 1440001_PM_at | 3.94E-04 | 3.66E-06 | 9.35 | 4.930578 | 1.58 | Rian |
| 1421351_PM_at | 3.96E-04 | 3.68E-06 | 9.34 | 4.922642 | 1.45 | Gria4 |
| 1428853_PM_at | 3.96E-04 | 3.71E-06 | -9.33 | 4.916365 | -1.05 | Ptch1 |
| 1416832_PM_at | 3.96E-04 | 3.71E-06 | -9.33 | 4.915589 | -1.68 | Slc39a8 |
| 1453931_PM_at | 3.99E-04 | 3.75E-06 | 9.32 | 4.905185 | 2.69 | Col14a1 |
| 1422140_PM_at | 4.01E-04 | 3.78E-06 | -9.31 | 4.896512 | -1.44 | Csprs |
| 1416514_PM_a_at | 4.02E-04 | 3.82E-06 | 9.3 | 4.88704 | 2.09 | Fscn1 |
| 1437668_PM_at | 4.02E-04 | 3.82E-06 | -9.3 | 4.885985 | -2.41 | Ackr4///Acad11 |
| 1424659_PM_at | 4.07E-04 | 3.90E-06 | -9.28 | 4.863481 | -1.04 | Slit2 |
| 1433862_PM_at | 4.07E-04 | 3.93E-06 | 9.27 | 4.855853 | 1.09 | Espl1 |
| 1422788_PM_at | 4.07E-04 | 3.95E-06 | -9.26 | 4.850853 | -1.63 | Slc43a3 |
| 1425587_PM_a_at | 4.07E-04 | 3.95E-06 | -9.26 | 4.850389 | -1.2 | Ptprj |
| 1424214_PM_at | 4.13E-04 | 4.02E-06 | 9.24 | 4.832677 | 1.87 | Parm1 |
| 1435345_PM_at | 4.16E-04 | 4.10E-06 | 9.23 | 4.813696 | 1.18 | Cercam |
| 1427149_PM_at | 4.19E-04 | 4.18E-06 | -9.2 | 4.793629 | -2.07 | Plekha6 |
| 1448154_PM_at | 4.22E-04 | 4.23E-06 | -9.19 | 4.781616 | -1.57 | Ndrg2 |
| 1437669_PM_x_at | 4.31E-04 | 4.33E-06 | -9.17 | 4.757495 | -1.87 | Ackr4///Acad11 |
| 1433968_PM_a_at | 4.37E-04 | 4.41E-06 | -9.15 | 4.740272 | -1.04 | Megf9 |
| 1429236_PM_at | 4.49E-04 | 4.56E-06 | -9.11 | 4.704923 | -1.94 | Galnt15 |
| 1439795_PM_at | 4.55E-04 | 4.65E-06 | -9.09 | 4.684331 | -1.51 | Gm15241///Adgrg2 |
| 1460604_PM_at | 4.61E-04 | 4.74E-06 | 9.07 | 4.665047 | 1.68 | Cybrd1 |
| 1437052_PM_s_at | 4.63E-04 | 4.78E-06 | -9.07 | 4.658092 | -2.01 | Slc2a3 |
| 1418497_PM_at | 4.67E-04 | 4.85E-06 | -9.05 | 4.6422 | -1.26 | Fgf13 |
| 1421594_PM_a_at | 4.72E-04 | 4.92E-06 | 9.04 | 4.627281 | 1.04 | Sytl2 |
| 1440668_PM_at | 4.72E-04 | 4.94E-06 | -9.03 | 4.624367 | -1.48 | Adamtsl3 |
| 1437753_PM_at | 4.73E-04 | 4.98E-06 | -9.02 | 4.615828 | -1.11 | Faxc |
| 1417267_PM_s_at | 4.78E-04 | 5.04E-06 | 9.01 | 4.602227 | 2.02 | Fkbp11 |
| 1444232_PM_at | 5.05E-04 | 5.48E-06 | -8.92 | 4.516949 | -1.28 | Prkg1 |
| 1435849_PM_at | 5.14E-04 | 5.65E-06 | -8.89 | 4.48696 | -1.17 | Jakmip3 |
| 1434905_PM_at | 5.38E-04 | 5.97E-06 | 8.84 | 4.429314 | 1.32 | Ndufa4l2 |
| 1423311_PM_s_at | 5.42E-04 | 6.05E-06 | -8.82 | 4.415469 | -1.38 | Tpbg |
| 1442707_PM_at | 5.56E-04 | 6.31E-06 | 8.78 | 4.373553 | 1.65 | Camk2a |
| 1437865_PM_at | 5.63E-04 | 6.42E-06 | -8.76 | 4.355283 | -1.25 | Spata13 |
| 1454656_PM_at | 5.79E-04 | 6.70E-06 | -8.72 | 4.312473 | -1.34 | Spata13 |
| 1436115_PM_at | 5.79E-04 | 6.70E-06 | 8.72 | 4.312229 | 1.29 | Gm266 |
| 1449273_PM_at | 5.87E-04 | 6.93E-06 | -8.69 | 4.277592 | -1.62 | Cyfip2 |
| 1416635_PM_at | 6.04E-04 | 7.26E-06 | -8.64 | 4.230011 | -1.04 | Smpdl3a |
| 1448550_PM_at | 6.10E-04 | 7.35E-06 | -8.63 | 4.2163 | -1.09 | Lbp |
| 1450780_PM_s_at | 6.28E-04 | 7.77E-06 | -8.57 | 4.160123 | -1.04 | Hmga2 |
| 1448378_PM_at | 6.29E-04 | 7.79E-06 | 8.57 | 4.156553 | 1.68 | Fscn1 |
| 1435722_PM_at | 6.51E-04 | 8.23E-06 | 8.52 | 4.101289 | 1.43 | Gria4 |
| 1455898_PM_x_at | 6.51E-04 | 8.23E-06 | -8.52 | 4.100168 | -1.66 | Slc2a3 |
| 1460419_PM_a_at | 6.69E-04 | 8.52E-06 | 8.48 | 4.065053 | 1.09 | Prkcb |
| 1428765_PM_at | 6.95E-04 | 8.97E-06 | 8.43 | 4.011875 | 1.2 | Meg3 |
| 1449876_PM_at | 7.13E-04 | 9.31E-06 | -8.4 | 3.973642 | -1.9 | Prkg1 |
| 1447520_PM_at | 7.38E-04 | 9.75E-06 | -8.35 | 3.926134 | -1.12 | Lbp |
| 1421359_PM_at | 7.49E-04 | 9.99E-06 | 8.33 | 3.901206 | 1.52 | Ret |
| 1457619_PM_at | 7.74E-04 | 1.05E-05 | 8.28 | 3.852676 | 2.18 | Ces2b |
| 1448789_PM_at | 7.78E-04 | 1.06E-05 | -8.27 | 3.842223 | -1.1 | Aldh1a3 |
| 1438350_PM_at | 7.96E-04 | 1.10E-05 | -8.23 | 3.799734 | -1.66 | Gm15241 |
| 1419603_PM_at | 8.18E-04 | 1.15E-05 | -8.19 | 3.758219 | -1.76 | Ifi204 |
| 1437149_PM_at | 8.18E-04 | 1.15E-05 | -8.19 | 3.754448 | -1.81 | Slc6a6 |
| 1418176_PM_at | 8.18E-04 | 1.15E-05 | -8.19 | 3.753463 | -1.15 | Vdr |
| 1425814_PM_a_at | 8.18E-04 | 1.15E-05 | -8.19 | 3.753233 | -1.26 | Calcrl |
| 1429214_PM_at | 8.28E-04 | 1.18E-05 | 8.17 | 3.727575 | 1.15 | Adamtsl2 |
| 1449565_PM_at | 8.30E-04 | 1.19E-05 | -8.16 | 3.720853 | -3.26 | Cyp2g1 |
| 1448507_PM_at | 8.48E-04 | 1.23E-05 | -8.13 | 3.683492 | -1.43 | Efhd1 |
| 1435342_PM_at | 8.53E-04 | 1.25E-05 | 8.11 | 3.670429 | 1.08 | Kcnk6 |
| 1435050_PM_at | 8.57E-04 | 1.27E-05 | 8.1 | 3.658014 | 1.08 | Arfgef3 |
| 1442608_PM_at | 8.57E-04 | 1.27E-05 | -8.1 | 3.656661 | -1.02 | Layn |
| 1434474_PM_at | 8.88E-04 | 1.33E-05 | -8.05 | 3.604551 | -1.16 | Abca5 |
| 1450014_PM_at | 8.96E-04 | 1.35E-05 | -8.04 | 3.589229 | -1.14 | Cldn1 |
| 1427325_PM_s_at | 9.23E-04 | 1.42E-05 | -7.99 | 3.540457 | -1.05 | Akna |
| 1436095_PM_at | 9.27E-04 | 1.43E-05 | 7.99 | 3.533223 | 1.01 | Chd5 |
| 1422837_PM_at | 9.29E-04 | 1.44E-05 | -7.98 | 3.523947 | -2.29 | Scel |
| 1422955_PM_at | 9.30E-04 | 1.45E-05 | -7.97 | 3.516306 | -1.06 | Syt17 |
| 1428471_PM_at | 9.30E-04 | 1.45E-05 | -7.97 | 3.51579 | -1.05 | Sorbs1 |
| 1455995_PM_at | 9.38E-04 | 1.47E-05 | 7.96 | 3.501045 | 1.92 | Arfgef3 |
| 1418260_PM_at | 9.74E-04 | 1.57E-05 | 7.9 | 3.437666 | 1.04 | Hunk |
| 1428384_PM_at | 9.83E-04 | 1.60E-05 | -7.88 | 3.417916 | -1.1 | Lurap1l |
| 1416332_PM_at | 9.83E-04 | 1.60E-05 | -7.88 | 3.417627 | -1.33 | Cirbp |
| 1450484_PM_a_at | 9.95E-04 | 1.63E-05 | -7.87 | 3.397695 | -1.28 | Cmpk2 |
| 1425452_PM_s_at | 1.01E-03 | 1.68E-05 | 7.84 | 3.364927 | 1.33 | Fam84a |
| 1453377_PM_at | 1.04E-03 | 1.75E-05 | -7.8 | 3.325123 | -1.38 | Sh2d4a |
| 1455447_PM_at | 1.05E-03 | 1.77E-05 | 7.79 | 3.309982 | 1.22 | D430019H16Rik |
| 1448681_PM_at | 1.09E-03 | 1.88E-05 | -7.74 | 3.251577 | -1.1 | Il15ra |
| 1416953_PM_at | 1.10E-03 | 1.90E-05 | -7.72 | 3.237841 | -2.04 | Ctgf |
| 1417599_PM_at | 1.10E-03 | 1.91E-05 | 7.72 | 3.234456 | 1.15 | Cd276 |
| 1447933_PM_at | 1.11E-03 | 1.93E-05 | 7.71 | 3.221133 | 1.06 | Kif26a |
| 1424306_PM_at | 1.15E-03 | 2.04E-05 | -7.66 | 3.16526 | -2 | Elovl4 |
| 1454966_PM_at | 1.18E-03 | 2.15E-05 | -7.61 | 3.109182 | -1.25 | Itga8 |
| 1442226_PM_at | 1.18E-03 | 2.16E-05 | -7.61 | 3.107995 | -1.13 | Sema3e |
| 1419161_PM_a_at | 1.19E-03 | 2.22E-05 | -7.59 | 3.079778 | -1.04 | Nox4 |
| 1437214_PM_at | 1.21E-03 | 2.25E-05 | 7.57 | 3.063102 | 1.28 | Lrrtm4 |
| 1416666_PM_at | 1.25E-03 | 2.38E-05 | -7.52 | 3.006403 | -1.4 | Serpine2 |
| 1439030_PM_at | 1.25E-03 | 2.38E-05 | 7.52 | 3.004351 | 1.12 | Gmppb |
| 1418674_PM_at | 1.25E-03 | 2.39E-05 | -7.52 | 3.00327 | -1.15 | Osmr |
| 1419376_PM_at | 1.27E-03 | 2.44E-05 | -7.5 | 2.981086 | -2.51 | Fibin |
| 1418675_PM_at | 1.27E-03 | 2.45E-05 | -7.5 | 2.977015 | -1.02 | Osmr |
| 1442257_PM_at | 1.28E-03 | 2.48E-05 | 7.48 | 2.963701 | 1.07 | Cdh6 |
| 1442542_PM_at | 1.28E-03 | 2.49E-05 | -7.48 | 2.959569 | -1.13 | Eya4 |
| 1454801_PM_at | 1.29E-03 | 2.52E-05 | -7.47 | 2.947611 | -1.19 | Ankrd28///3110068a07rik |
| 1451827_PM_a_at | 1.29E-03 | 2.53E-05 | -7.47 | 2.942999 | -1.13 | Nox4 |
| 1422975_PM_at | 1.30E-03 | 2.53E-05 | -7.46 | 2.940754 | -1.45 | Mme |
| 1418780_PM_at | 1.32E-03 | 2.59E-05 | -7.45 | 2.918451 | -1.01 | Cyp39a1 |
| 1436821_PM_at | 1.35E-03 | 2.68E-05 | -7.41 | 2.881606 | -1.05 | Plcxd3 |
| 1415856_PM_at | 1.44E-03 | 2.98E-05 | -7.32 | 2.773876 | -1.12 | Emb |
| 1434630_PM_at | 1.46E-03 | 3.00E-05 | -7.31 | 2.764274 | -1.19 | Ankrd28///3110068a07rik |
| 1422293_PM_a_at | 1.46E-03 | 3.01E-05 | -7.31 | 2.761911 | -1.19 | Kctd1 |
| 1441958_PM_s_at | 1.46E-03 | 3.02E-05 | 7.31 | 2.759451 | 1 | Ager |
| 1436212_PM_at | 1.47E-03 | 3.06E-05 | -7.3 | 2.744449 | -1.29 | Tmem71 |
| 1429671_PM_at | 1.48E-03 | 3.13E-05 | 7.28 | 2.72296 | 1.08 | Zbed5 |
| 1451308_PM_at | 1.51E-03 | 3.23E-05 | -7.25 | 2.688182 | -1.73 | Elovl4 |
| 1424882_PM_a_at | 1.51E-03 | 3.24E-05 | 7.25 | 2.686833 | 1.27 | Nt5dc2 |
| 1418489_PM_a_at | 1.61E-03 | 3.55E-05 | -7.17 | 2.590819 | -1.5 | Calcrl |
| 1426338_PM_a_at | 1.63E-03 | 3.60E-05 | -7.16 | 2.577938 | -1.27 | Ntng1 |
| 1428733_PM_at | 1.65E-03 | 3.66E-05 | -7.14 | 2.559488 | -1.34 | Gngt2 |
| 1438232_PM_at | 1.66E-03 | 3.70E-05 | -7.13 | 2.547928 | -1.45 | Foxp2 |
| 1457030_PM_at | 1.68E-03 | 3.76E-05 | 7.12 | 2.530876 | 2.71 | Mirg |
| 1418788_PM_at | 1.70E-03 | 3.83E-05 | -7.1 | 2.513487 | -1.73 | Tek |
| 1454984_PM_at | 1.71E-03 | 3.89E-05 | -7.09 | 2.495599 | -1.15 | Lifr |
| 1437128_PM_a_at | 1.81E-03 | 4.22E-05 | -7.02 | 2.4107 | -1.05 | Zfp945 |
| 1418537_PM_at | 1.85E-03 | 4.36E-05 | 6.99 | 2.377775 | 1 | Isoc2b |
| 1419225_PM_at | 1.86E-03 | 4.42E-05 | 6.98 | 2.364016 | 1.45 | Cacna2d3 |
| 1428891_PM_at | 1.90E-03 | 4.53E-05 | 6.96 | 2.337999 | 1.47 | Parm1 |
| 1434581_PM_at | 1.92E-03 | 4.61E-05 | -6.94 | 2.318704 | -1.13 | Mturn |
| 1452139_PM_at | 1.94E-03 | 4.71E-05 | 6.93 | 2.298413 | 1.06 | Gm4026///Slc35c1 |
| 1416474_PM_at | 1.94E-03 | 4.71E-05 | 6.93 | 2.29662 | 1.2 | Igdcc4 |
| 1435857_PM_s_at | 1.95E-03 | 4.75E-05 | 6.92 | 2.288055 | 1.64 | Aplp1 |
| 1418945_PM_at | 2.10E-03 | 5.27E-05 | 6.83 | 2.180234 | 1.09 | Mmp3 |
| 1437308_PM_s_at | 2.11E-03 | 5.29E-05 | -6.83 | 2.176559 | -1.3 | F2r |
| 1460578_PM_at | 2.15E-03 | 5.42E-05 | 6.81 | 2.151115 | 1.33 | Fgd5 |
| 1420928_PM_at | 2.17E-03 | 5.50E-05 | -6.8 | 2.137044 | -1.16 | St6gal1 |
| 1422643_PM_at | 2.21E-03 | 5.60E-05 | 6.78 | 2.116783 | 1.18 | Moxd1 |
| 1419490_PM_at | 2.21E-03 | 5.68E-05 | -6.77 | 2.103143 | -1.52 | Fam19a5 |
| 1448628_PM_at | 2.22E-03 | 5.70E-05 | 6.77 | 2.099355 | 1.18 | Scg3 |
| 1425040_PM_at | 2.27E-03 | 5.89E-05 | 6.74 | 2.064317 | 1.56 | Cybrd1 |
| 1439426_PM_x_at | 2.31E-03 | 6.06E-05 | -6.72 | 2.036134 | -1.08 | Lyz2 |
| 1416772_PM_at | 2.32E-03 | 6.14E-05 | 6.71 | 2.021935 | 1.06 | Cpt2 |
| 1428043_PM_a_at | 2.35E-03 | 6.24E-05 | 6.69 | 2.00528 | 1.47 | Cmah |
| 1450648_PM_s_at | 2.35E-03 | 6.24E-05 | -6.69 | 2.004857 | -1.14 | H2-Ab1 |
| 1437932_PM_a_at | 2.45E-03 | 6.62E-05 | -6.64 | 1.942934 | -1.22 | Cldn1 |
| 1435180_PM_at | 2.51E-03 | 6.86E-05 | 6.62 | 1.907126 | 1.14 | Podn |
| 1416892_PM_s_at | 2.53E-03 | 6.96E-05 | -6.6 | 1.89163 | -1.27 | Fam107b |
| 1423280_PM_at | 2.56E-03 | 7.08E-05 | -6.59 | 1.873014 | -1.62 | Stmn2 |
| 1450243_PM_a_at | 2.58E-03 | 7.16E-05 | -6.58 | 1.861871 | -1.26 | Rcan2 |
| 1434558_PM_at | 2.62E-03 | 7.36E-05 | -6.56 | 1.833877 | -1 | Wdr47 |
| 1428402_PM_at | 2.65E-03 | 7.47E-05 | 6.55 | 1.817068 | 1.08 | Zcchc3 |
| 1435409_PM_at | 2.66E-03 | 7.58E-05 | -6.53 | 1.802852 | -1.31 | Tmem26 |
| 1436043_PM_at | 2.78E-03 | 8.15E-05 | -6.48 | 1.727556 | -1.11 | Scn7a |
| 1421403_PM_at | 2.82E-03 | 8.37E-05 | 6.45 | 1.699024 | 1.14 | Pi15 |
| 1448509_PM_at | 2.83E-03 | 8.42E-05 | -6.45 | 1.693029 | -1.26 | Fam107b |
| 1427537_PM_at | 2.89E-03 | 8.63E-05 | -6.43 | 1.667609 | -1.36 | Eppk1 |
| 1437492_PM_at | 2.89E-03 | 8.66E-05 | -6.43 | 1.663904 | -1.11 | Mkx |
| 1439827_PM_at | 2.92E-03 | 8.81E-05 | 6.41 | 1.646319 | 1.01 | Adamts12 |
| 1448591_PM_at | 3.06E-03 | 9.43E-05 | -6.36 | 1.575108 | -1.29 | Ctss |
| 1423312_PM_at | 3.14E-03 | 9.71E-05 | -6.34 | 1.544216 | -1.34 | Tpbg |
| 1419703_PM_at | 3.14E-03 | 9.72E-05 | 6.33 | 1.543108 | 1.02 | Col5a3 |
| 1449751_PM_at | 3.14E-03 | 9.73E-05 | -6.33 | 1.542525 | -1.65 | Slc6a6 |
| 1419136_PM_at | 3.20E-03 | 1.00E-04 | -6.31 | 1.512722 | -1.37 | Akr1c18 |
| 1429564_PM_at | 3.43E-03 | 1.10E-04 | -6.23 | 1.411898 | -1.02 | Pcgf5 |
| 1455325_PM_at | 3.62E-03 | 1.20E-04 | 6.17 | 1.325432 | 1.39 | Miat |
| 1416021_PM_a_at | 3.65E-03 | 1.22E-04 | -6.16 | 1.309679 | -1.25 | Fabp5 |
| 1416612_PM_at | 3.70E-03 | 1.24E-04 | -6.14 | 1.288846 | -1.02 | 1700038P13Rik///Cyp1b1 |
| 1453345_PM_at | 3.74E-03 | 1.26E-04 | -6.13 | 1.273715 | -1.21 | Nipal1 |
| 1449770_PM_x_at | 3.80E-03 | 1.29E-04 | -6.11 | 1.249738 | -1.09 | Tmem191c |
| 1439183_PM_at | 3.84E-03 | 1.32E-04 | -6.1 | 1.228082 | -1.76 | Acer1 |
| 1415857_PM_at | 3.86E-03 | 1.33E-04 | -6.09 | 1.219431 | -1.21 | Emb |
| 1449865_PM_at | 3.89E-03 | 1.35E-04 | 6.08 | 1.202623 | 1.09 | Sema3a |
| 1428347_PM_at | 4.22E-03 | 1.51E-04 | -5.99 | 1.081022 | -1.46 | Cyfip2 |
| 1435195_PM_at | 4.42E-03 | 1.61E-04 | 5.94 | 1.014376 | 1.15 | Vash1 |
| 1421079_PM_at | 4.45E-03 | 1.63E-04 | -5.93 | 1.004818 | -1.79 | Nr4a3 |
| 1416745_PM_x_at | 4.59E-03 | 1.70E-04 | -5.9 | 0.96184 | -1.02 | Uap1 |
| 1454838_PM_s_at | 4.59E-03 | 1.70E-04 | 5.9 | 0.95893 | 1.34 | Pkdcc |
| 1455452_PM_x_at | 4.64E-03 | 1.73E-04 | 5.89 | 0.941612 | 1.1 | Kctd14 |
| 1418918_PM_at | 4.67E-03 | 1.75E-04 | 5.88 | 0.93183 | 1.24 | Igfbp1 |
| 1455333_PM_at | 4.70E-03 | 1.77E-04 | -5.87 | 0.920938 | -1.23 | Tns3 |
| 1417561_PM_at | 4.70E-03 | 1.77E-04 | -5.87 | 0.9209 | -1.35 | Apoc1 |
| 1455872_PM_at | 4.77E-03 | 1.79E-04 | 5.86 | 0.904666 | 1.46 | Fam167a |
| 1453317_PM_a_at | 4.79E-03 | 1.80E-04 | 5.86 | 0.899621 | 1.08 | Khdrbs3 |
| 1416613_PM_at | 4.82E-03 | 1.82E-04 | -5.85 | 0.888378 | -1.36 | 1700038P13Rik///Cyp1b1 |
| 1439284_PM_at | 4.83E-03 | 1.83E-04 | -5.84 | 0.884495 | -1.07 | Epb41l5 |
| 1416022_PM_at | 4.93E-03 | 1.89E-04 | -5.82 | 0.848309 | -1.37 | Fabp5 |
| 1420148_PM_at | 5.22E-03 | 2.06E-04 | -5.75 | 0.759601 | -1.41 | Slc6a6 |
| 1418829_PM_a_at | 5.37E-03 | 2.17E-04 | -5.72 | 0.707101 | -1.41 | Eno2 |
| 1418175_PM_at | 5.41E-03 | 2.21E-04 | -5.7 | 0.68863 | -1.36 | Vdr |
| 1440108_PM_at | 5.50E-03 | 2.26E-04 | -5.69 | 0.662836 | -1.23 | Foxp2 |
| 1419829_PM_a_at | 5.58E-03 | 2.30E-04 | -5.67 | 0.64389 | -1.08 | Gab2 |
| 1437167_PM_at | 5.60E-03 | 2.31E-04 | -5.67 | 0.638741 | -1.13 | Tmod2 |
| 1438796_PM_at | 5.69E-03 | 2.36E-04 | -5.65 | 0.617082 | -1.7 | Nr4a3 |
| 1437671_PM_x_at | 5.72E-03 | 2.39E-04 | -5.64 | 0.605616 | -1.32 | Prss23///Prss23os |
| 1423281_PM_at | 5.81E-03 | 2.44E-04 | -5.63 | 0.582435 | -1.49 | Stmn2 |
| 1417049_PM_at | 5.90E-03 | 2.51E-04 | 5.61 | 0.554556 | 1.59 | Rhd |
| 1425428_PM_at | 6.12E-03 | 2.65E-04 | 5.57 | 0.497155 | 1.11 | Hif3a |
| 1416744_PM_at | 6.35E-03 | 2.79E-04 | -5.53 | 0.443192 | -1.04 | Uap1 |
| 1424902_PM_at | 6.39E-03 | 2.82E-04 | -5.52 | 0.43234 | -1 | Plxdc1 |
| 1422638_PM_s_at | 6.68E-03 | 3.04E-04 | -5.47 | 0.355857 | -1.4 | Rassf5 |
| 1423935_PM_x_at | 6.85E-03 | 3.16E-04 | -5.44 | 0.315478 | -1.65 | Krt14 |
| 1421852_PM_at | 6.96E-03 | 3.22E-04 | -5.43 | 0.294663 | -1.04 | Kcnk5 |
| 1423804_PM_a_at | 7.03E-03 | 3.28E-04 | -5.41 | 0.276389 | -1.24 | Idi1 |
| 1417023_PM_a_at | 7.13E-03 | 3.35E-04 | -5.4 | 0.254622 | -2.22 | Fabp4 |
| 1425546_PM_a_at | 7.33E-03 | 3.51E-04 | -5.36 | 0.204999 | -1.07 | Trf |
| 1449586_PM_at | 7.38E-03 | 3.55E-04 | 5.36 | 0.192259 | 1.42 | Pkp1 |
| 1419073_PM_at | 7.38E-03 | 3.55E-04 | -5.36 | 0.191792 | -1.14 | Tmeff2 |
| 1435110_PM_at | 7.72E-03 | 3.83E-04 | 5.3 | 0.114741 | 1 | Unc5b |
| 1419426_PM_s_at | 7.79E-03 | 3.89E-04 | -5.29 | 0.097244 | -1 | Ccl21b |
| 1455889_PM_at | 7.88E-03 | 3.96E-04 | -5.28 | 0.079261 | -1.94 | Far2 |
| 1419420_PM_at | 8.00E-03 | 4.04E-04 | -5.26 | 0.056657 | -1.38 | St6galnac5 |
| 1440186_PM_s_at | 8.39E-03 | 4.34E-04 | -5.21 | -0.017073 | -2.06 | Psapl1 |
| 1431057_PM_a_at | 9.00E-03 | 4.81E-04 | -5.14 | -0.123723 | -1.62 | Prss23 |
| 1436939_PM_at | 9.21E-03 | 4.99E-04 | -5.11 | -0.162612 | -1.39 | Unc45b///Slfn5os |
| 1439573_PM_at | 9.52E-03 | 5.24E-04 | -5.08 | -0.213746 | -1.09 | Rtn4rl2 |
| 1421117_PM_at | 9.80E-03 | 5.46E-04 | -5.05 | -0.256272 | -1.65 | Dst |
| 1440888_PM_at | 9.82E-03 | 5.48E-04 | 5.05 | -0.259798 | 1.8 | Oxtr |
| 1451263_PM_a_at | 1.00E-02 | 5.64E-04 | -5.03 | -0.289179 | -3.02 | Fabp4 |
| 1436293_PM_x_at | 1.04E-02 | 5.96E-04 | -4.99 | -0.346929 | -1.3 | Ildr2 |
| 1424451_PM_at | 1.05E-02 | 6.13E-04 | -4.97 | -0.377493 | -1.29 | Acaa1b |
| 1448595_PM_a_at | 1.08E-02 | 6.31E-04 | -4.95 | -0.407615 | -1.41 | Bex1 |
| 1418979_PM_at | 1.12E-02 | 6.71E-04 | -4.91 | -0.471485 | -2.42 | Akr1c14 |
| 1440973_PM_at | 1.12E-02 | 6.76E-04 | -4.9 | -0.478629 | -1.02 | LOC552874 |
| 1421080_PM_at | 1.17E-02 | 7.18E-04 | -4.86 | -0.541136 | -1.24 | Nr4a3 |
| 1421214_PM_at | 1.21E-02 | 7.56E-04 | 4.83 | -0.595792 | 1.37 | Cmah |
| 1460347_PM_at | 1.23E-02 | 7.84E-04 | -4.8 | -0.633134 | -1.59 | Krt14 |
| 1420401_PM_a_at | 1.29E-02 | 8.41E-04 | 4.75 | -0.705987 | 1.1 | Ramp3 |
| 1419414_PM_at | 1.31E-02 | 8.51E-04 | 4.75 | -0.718602 | 1.04 | Gng13 |
| 1435851_PM_at | 1.35E-02 | 8.87E-04 | 4.72 | -0.761102 | 1.33 | Lgi1 |
| 1417487_PM_at | 1.38E-02 | 9.25E-04 | -4.69 | -0.80507 | -1.94 | Fosl1 |
| 1417488_PM_at | 1.39E-02 | 9.33E-04 | -4.69 | -0.81359 | -1.52 | Fosl1 |
| 1446560_PM_at | 1.43E-02 | 9.73E-04 | -4.66 | -0.857504 | -1.06 | Prss23///Prss23os |
| 1416619_PM_at | 1.44E-02 | 9.84E-04 | -4.65 | -0.868855 | -1.19 | Vsir |
| 1426960_PM_a_at | 1.44E-02 | 9.89E-04 | -4.65 | -0.874858 | -1.5 | Fa2h |
| 1449009_PM_at | 1.45E-02 | 1.00E-03 | -4.64 | -0.888293 | -1.26 | Tgtp1 |
| 1453261_PM_at | 1.46E-02 | 1.01E-03 | -4.64 | -0.891295 | -1.06 | 2610035D17Rik |
| 1449468_PM_at | 1.54E-02 | 1.09E-03 | -4.58 | -0.979957 | -1.05 | St6galnac5 |
| 1455299_PM_at | 1.57E-02 | 1.13E-03 | -4.56 | -1.011642 | -1.02 | Vgll3 |
| 1427946_PM_s_at | 1.57E-02 | 1.13E-03 | -4.56 | -1.01388 | -1.09 | Dpyd |
| 1430503_PM_at | 1.98E-02 | 1.61E-03 | 4.32 | -1.382539 | 1.07 | 6330522J23Rik |
| 1435060_PM_at | 2.05E-02 | 1.71E-03 | -4.29 | -1.442557 | -1.03 | Tmod2 |
| 1429267_PM_at | 2.05E-02 | 1.71E-03 | 4.28 | -1.444469 | 1.18 | Acot11///Fam151a |
| 1425411_PM_at | 2.10E-02 | 1.76E-03 | -4.27 | -1.475074 | -1.32 | Arl4a |
| 1448110_PM_at | 2.10E-02 | 1.77E-03 | -4.26 | -1.478079 | -1.61 | Sema4a |
| 1429159_PM_at | 2.11E-02 | 1.78E-03 | -4.26 | -1.487251 | -1.05 | Itih5 |
| 1422749_PM_at | 2.19E-02 | 1.90E-03 | -4.22 | -1.549339 | -1.16 | Ly6g6c |
| 1418937_PM_at | 2.22E-02 | 1.93E-03 | -4.21 | -1.567331 | -1.2 | Dio2 |
| 1417403_PM_at | 2.30E-02 | 2.03E-03 | -4.18 | -1.620296 | -1.06 | Elovl6 |
| 1443143_PM_at | 2.40E-02 | 2.15E-03 | 4.14 | -1.678719 | 2.23 | Flrt1 |
| 1449106_PM_at | 2.46E-02 | 2.23E-03 | 4.11 | -1.718615 | 1.07 | Gpx3 |
| 1427352_PM_at | 2.58E-02 | 2.40E-03 | -4.07 | -1.792201 | -1.21 | Krt79 |
| 1425789_PM_s_at | 2.65E-02 | 2.49E-03 | -4.04 | -1.831627 | -1.3 | Anxa8 |
| 1417574_PM_at | 2.74E-02 | 2.62E-03 | -4.01 | -1.885147 | -1.08 | Cxcl12 |
| 1417404_PM_at | 2.95E-02 | 2.93E-03 | -3.94 | -2.000199 | -1.5 | Elovl6 |
| 1418872_PM_at | 3.04E-02 | 3.08E-03 | -3.91 | -2.05181 | -1.04 | Abcb1b |
| 1448471_PM_a_at | 3.59E-02 | 3.96E-03 | -3.75 | -2.309605 | -1.38 | Ctla2a |
| 1453287_PM_at | 3.71E-02 | 4.17E-03 | -3.72 | -2.362629 | -1.29 | Ankrd33b |
| 1450930_PM_at | 3.83E-02 | 4.36E-03 | -3.69 | -2.409724 | -1.27 | Hpca |
| 1417732_PM_at | 3.84E-02 | 4.38E-03 | -3.69 | -2.414405 | -1.31 | Anxa8 |
| 1437667_PM_a_at | 3.96E-02 | 4.59E-03 | -3.66 | -2.461428 | -1.04 | Bach2 |
| 1436039_PM_at | 4.09E-02 | 4.82E-03 | 3.63 | -2.511878 | 1.07 | Cmah |
| 1436515_PM_at | 4.18E-02 | 5.01E-03 | -3.61 | -2.550127 | -1.25 | Bach2 |
| 1435436_PM_at | 4.33E-02 | 5.27E-03 | -3.58 | -2.603685 | -1.06 | Epas1 |
| 1437270_PM_a_at | 4.36E-02 | 5.33E-03 | -3.57 | -2.613753 | -1.19 | Clcf1-pold4///Clcf1 |
| 1437689_PM_x_at | 4.43E-02 | 5.45E-03 | -3.56 | -2.638169 | -1.02 | Clu |
| 1459585_PM_at | 4.64E-02 | 5.79E-03 | 3.52 | -2.699518 | 1.24 | Gas6 |
| 1423858_PM_a_at | 4.78E-02 | 6.08E-03 | -3.49 | -2.749534 | -1.12 | Hmgcs2 |
| 1418301_PM_at | 4.78E-02 | 6.09E-03 | -3.49 | -2.75091 | -1.77 | Irf6 |
| 1424096_PM_at | 4.90E-02 | 6.35E-03 | -3.46 | -2.793133 | -1.04 | Krt5 |
| 1416271_PM_at | 4.95E-02 | 6.43E-03 | -3.46 | -2.806134 | -1.62 | Perp |

**Table S3 GSE117763 Full length DEGs**

| ID | adj.P.Val | P.Value | t | B | logFC | Gene.symbol |
| --- | --- | --- | --- | --- | --- | --- |
| 1424688_PM_at | 0.0000191 | 6.34E-10 | 2.68E+01 | 9.968972 | 2.67 | Creb3l3 |
| 1452906_PM_at | 0.0000191 | 8.47E-10 | 2.59E+01 | 9.864947 | 2.39 | Meg3 |
| 1440001_PM_at | 0.0000379 | 2.96E-09 | 2.25E+01 | 9.361484 | 2.55 | Rian |
| 1428055_PM_at | 0.0000379 | 3.36E-09 | 2.22E+01 | 9.306168 | 2.62 | Rian |
| 1426758_PM_s_at | 0.0000533 | 7.82E-09 | 2.02E+01 | 8.908884 | 1.69 | Meg3 |
| 1429257_PM_at | 0.0000533 | 7.86E-09 | 2.02E+01 | 8.906705 | 1.88 | Meg3 |
| 1428764_PM_at | 0.0000533 | 8.27E-09 | 2.01E+01 | 8.881221 | 2.48 | Meg3 |
| 1450871_PM_a_at | 0.0000758 | 1.64E-08 | 1.86E+01 | 8.526176 | 4.11 | Bcat1 |
| 1429256_PM_at | 0.0000758 | 1.84E-08 | 1.83E+01 | 8.464625 | 2.4 | Meg3 |
| 1452905_PM_at | 0.0001635 | 4.90E-08 | 1.64E+01 | 7.898648 | 1.56 | Meg3 |
| 1452183_PM_a_at | 0.0001635 | 5.07E-08 | 1.63E+01 | 7.877301 | 2.25 | Meg3 |
| 1441228_PM_at | 0.000195 | 6.48E-08 | 1.59E+01 | 7.726131 | 1.74 | Apold1 |
| 1450276_PM_a_at | 0.000234 | 8.30E-08 | -1.55E+01 | 7.57044 | -1.89 | Scin |
| 1452899_PM_at | 0.0002646 | 1.06E-07 | 1.50E+01 | 7.415365 | 3.08 | Rian |
| 1439622_PM_at | 0.0002958 | 1.25E-07 | 1.48E+01 | 7.306582 | 2.43 | Rassf4 |
| 1416183_PM_a_at | 0.0003009 | 1.33E-07 | -1.46E+01 | 7.261182 | -1.87 | Ldhb |
| 1436713_PM_s_at | 0.0003299 | 1.58E-07 | 1.44E+01 | 7.146329 | 2.41 | Meg3 |
| 1426338_PM_a_at | 0.0003299 | 1.61E-07 | -1.43E+01 | 7.135548 | -1.9 | Ntng1 |
| 1448237_PM_x_at | 0.0003395 | 1.73E-07 | -1.42E+01 | 7.085823 | -2.01 | Ldhb |
| 1436057_PM_at | 0.0005214 | 2.92E-07 | 1.34E+01 | 6.72145 | 1.29 | Meg3 |
| 1442449_PM_at | 0.0005214 | 3.00E-07 | 1.33E+01 | 6.701726 | 2.27 | Slc6a2 |
| 1427580_PM_a_at | 0.0006067 | 3.89E-07 | 1.29E+01 | 6.515629 | 3.53 | Rian |
| 1428765_PM_at | 0.0006067 | 3.90E-07 | 1.29E+01 | 6.513862 | 1.95 | Meg3 |
| 1431094_PM_at | 0.0006161 | 4.23E-07 | 1.28E+01 | 6.454598 | 2.8 | 1110006E14Rik |
| 1421641_PM_at | 0.0006509 | 4.61E-07 | 1.27E+01 | 6.391112 | 1.53 | Slc6a2 |
| 1424529_PM_s_at | 0.0006851 | 5.01E-07 | 1.26E+01 | 6.330639 | 2.08 | Cgref1 |
| 1436528_PM_at | 0.0007154 | 5.39E-07 | 1.25E+01 | 6.2765 | 1.28 | Kazald1 |
| 1452453_PM_a_at | 0.0012326 | 9.56E-07 | 1.16E+01 | 5.842161 | 1.94 | Camk2a |
| 1416514_PM_a_at | 0.0016201 | 1.33E-06 | 1.12E+01 | 5.585349 | 2.64 | Fscn1 |
| 1457030_PM_at | 0.0021365 | 1.80E-06 | 1.08E+01 | 5.343912 | 2.62 | Mirg |
| 1436119_PM_at | 0.0029193 | 2.66E-06 | 1.03E+01 | 5.027095 | 2.7 | Aldh1l2 |
| 1433877_PM_at | 0.0031055 | 2.96E-06 | -1.02E+01 | 4.938601 | -1.12 | Fam46b |
| 1416515_PM_at | 0.0031431 | 3.06E-06 | 1.01E+01 | 4.909643 | 1.21 | Fscn1 |
| 1448378_PM_at | 0.0041631 | 4.37E-06 | 9.72 | 4.612906 | 1.52 | Fscn1 |
| 1448152_PM_at | 0.0041631 | 4.45E-06 | -9.7 | 4.597706 | -2.27 | Igf2 |
| 1438121_PM_at | 0.0041631 | 4.50E-06 | 9.69 | 4.587968 | 1.13 | Sec24a |
| 1455325_PM_at | 0.0041631 | 4.50E-06 | 9.69 | 4.587814 | 1.81 | Miat |
| 1425475_PM_at | 0.0041631 | 4.52E-06 | 9.69 | 4.584884 | 1.54 | Col4a5 |
| 1439816_PM_at | 0.0050907 | 5.66E-06 | -9.43 | 4.393797 | -1.06 | Tcf24 |
| 1424528_PM_at | 0.0064431 | 8.14E-06 | 9.02 | 4.081766 | 1.8 | Cgref1 |
| 1417314_PM_at | 0.0079139 | 1.03E-05 | -8.76 | 3.872468 | -1.19 | Cfb |
| 1460129_PM_at | 0.0081459 | 1.08E-05 | 8.71 | 3.832391 | 2.19 | Slc6a2 |
| 1451611_PM_at | 0.0091703 | 1.24E-05 | -8.57 | 3.71356 | -1.46 | Pla2g16 |
| 1447311_PM_at | 0.0102942 | 1.44E-05 | 8.41 | 3.582641 | 1.59 | Slc6a2 |
| 1449172_PM_a_at | 0.0142842 | 2.32E-05 | 7.93 | 3.153421 | 1.61 | Lin7b |
| 1428168_PM_at | 0.0142842 | 2.34E-05 | 7.92 | 3.144622 | 1.35 | Mpzl1 |
| 1428167_PM_a_at | 0.0142842 | 2.38E-05 | 7.9 | 3.131094 | 1.67 | Mpzl1 |
| 1430111_PM_a_at | 0.0142842 | 2.42E-05 | 7.89 | 3.114874 | 1.01 | Bcat1 |
| 1416121_PM_at | 0.0142842 | 2.44E-05 | 7.88 | 3.108892 | 1.25 | Lox |
| 1418507_PM_s_at | 0.0153312 | 2.68E-05 | -7.78 | 3.021482 | -1.77 | Socs2 |
| 1424556_PM_at | 0.0153942 | 2.73E-05 | 7.77 | 3.00632 | 1.84 | Pycr1 |
| 1434580_PM_at | 0.0155831 | 2.80E-05 | -7.74 | 2.983937 | -1.19 | Enpp4 |
| 1430712_PM_at | 0.0161537 | 3.04E-05 | 7.66 | 2.907257 | 1.04 | Arhgap24 |
| 1415931_PM_at | 0.0177037 | 3.57E-05 | -7.51 | 2.761035 | -2.3 | Igf2 |
| 1437125_PM_at | 0.0177037 | 3.64E-05 | 7.49 | 2.744119 | 1.72 | Camk2a |
| 1439795_PM_at | 0.0177037 | 3.65E-05 | -7.49 | 2.741109 | -1.4 | Gm15241///Adgrg2 |
| 1423593_PM_a_at | 0.0179761 | 3.78E-05 | 7.45 | 2.707549 | 1.07 | Csf1r |
| 1428455_PM_at | 0.0209894 | 4.98E-05 | 7.2 | 2.453022 | 1.77 | Col14a1 |
| 1436337_PM_at | 0.0214243 | 5.17E-05 | -7.16 | 2.418623 | -1.24 | Tmem243///Gm17739 |
| 1421161_PM_at | 0.0214907 | 5.24E-05 | -7.15 | 2.407276 | -1.02 | Btc |
| 1436346_PM_at | 0.0221059 | 5.51E-05 | -7.11 | 2.359886 | -1.36 | Cd109 |
| 1449581_PM_at | 0.0221059 | 5.63E-05 | 7.09 | 2.339771 | 1.55 | Emid1 |
| 1435110_PM_at | 0.0224588 | 5.85E-05 | 7.05 | 2.304224 | 1.4 | Unc5b |
| 1417356_PM_at | 0.0232671 | 6.48E-05 | 6.96 | 2.208881 | 2.05 | Peg3 |
| 1423909_PM_at | 0.0232671 | 6.49E-05 | -6.96 | 2.207388 | -2.42 | Tmem176a |
| 1455280_PM_at | 0.0254378 | 7.44E-05 | 6.84 | 2.080022 | 1.07 | Frem1 |
| 1425658_PM_at | 0.0299803 | 9.70E-05 | -6.6 | 1.830751 | -1.08 | Cd109 |
| 1425040_PM_at | 0.0332351 | 1.11E-04 | 6.48 | 1.700735 | 1.6 | Cybrd1 |
| 1438588_PM_at | 0.0332351 | 1.13E-04 | 6.47 | 1.685788 | 1.24 | 2610311E24Rik |
| 1437149_PM_at | 0.0332351 | 1.13E-04 | -6.47 | 1.685522 | -1.12 | Slc6a6 |
| 1458602_PM_at | 0.0334458 | 1.15E-04 | -6.46 | 1.670787 | -1.08 | Bbx |
| 1427168_PM_a_at | 0.0336721 | 1.16E-04 | 6.45 | 1.658305 | 1.8 | Col14a1 |
| 1416741_PM_at | 0.0338736 | 1.18E-04 | 6.44 | 1.646597 | 1.34 | Col5a1 |
| 1417332_PM_at | 0.0352394 | 1.25E-04 | -6.39 | 1.592746 | -1.2 | Rfx2 |
| 1418004_PM_a_at | 0.0370847 | 1.37E-04 | -6.31 | 1.50209 | -1.42 | Tmem176b |
| 1423280_PM_at | 0.0370847 | 1.37E-04 | -6.31 | 1.501967 | -1.06 | Stmn2 |
| 1425546_PM_a_at | 0.0379984 | 1.44E-04 | -6.27 | 1.455059 | -1.07 | Trf |
| 1438350_PM_at | 0.0379984 | 1.45E-04 | -6.26 | 1.450725 | -1.33 | Gm15241 |
| 1441033_PM_at | 0.0403369 | 1.63E-04 | 6.16 | 1.33864 | 1.04 | Tmtc2 |
| 1424677_PM_at | 0.0408056 | 1.68E-04 | -6.14 | 1.308013 | -1.04 | Cyp2j9 |
| 1460419_PM_a_at | 0.0408056 | 1.69E-04 | 6.13 | 1.303016 | 1.38 | Prkcb |
| 1441811_PM_x_at | 0.0415442 | 1.76E-04 | -6.1 | 1.265108 | -2.05 | Tmem176a |
| 1455699_PM_at | 0.041563 | 1.79E-04 | 6.09 | 1.250259 | 1.31 | Bcat1 |
| 1418175_PM_at | 0.0420931 | 1.90E-04 | -6.04 | 1.192048 | -1.2 | Vdr |
| 1416051_PM_at | 0.0441887 | 2.06E-04 | -5.97 | 1.115663 | -1.27 | C2 |
| 1417355_PM_at | 0.0443222 | 2.08E-04 | 5.96 | 1.103673 | 1.53 | Peg3 |
| 1422140_PM_at | 0.0446658 | 2.12E-04 | -5.95 | 1.087252 | -1.21 | Csprs |
| 1424923_PM_at | 0.0455396 | 2.24E-04 | -5.91 | 1.034745 | -2.08 | Serpina3g |

**Table S4 GO and pathway enrichmenta table with Gene counts of 10 hub genes**

| ONTOLOGY | ID | Description | GeneRatio | BgRatio | pvalue | p.adjust | qvalue |
| --- | --- | --- | --- | --- | --- | --- | --- |
| BP | GO:0097300 | programmed necrotic cell death | 5/10 | 38/23210 | 2.24e-12 | 1.39e-09 | 4.67e-10 |
| BP | GO:0070265 | necrotic cell death | 5/10 | 51/23210 | 1.05e-11 | 3.25e-09 | 1.09e-09 |
| BP | GO:0051291 | protein heterooligomerization | 6/10 | 160/23210 | 2.00e-11 | 3.36e-09 | 1.13e-09 |
| BP | GO:0071356 | cellular response to tumor necrosis factor | 6/10 | 162/23210 | 2.16e-11 | 3.36e-09 | 1.13e-09 |
| BP | GO:0034612 | response to tumor necrosis factor | 6/10 | 179/23210 | 3.96e-11 | 4.93e-09 | 1.65e-09 |
| CC | GO:0045121 | membrane raft | 7/10 | 364/23436 | 2.37e-11 | 2.93e-10 | 1.28e-10 |
| CC | GO:0098857 | membrane microdomain | 7/10 | 365/23436 | 2.42e-11 | 2.93e-10 | 1.28e-10 |
| CC | GO:0098589 | membrane region | 7/10 | 377/23436 | 3.04e-11 | 2.93e-10 | 1.28e-10 |
| CC | GO:0043235 | receptor complex | 5/10 | 394/23436 | 3.08e-07 | 2.23e-06 | 9.72e-07 |
| CC | GO:0035631 | CD40 receptor complex | 2/10 | 11/23436 | 8.99e-06 | 5.22e-05 | 2.27e-05 |
| MF | GO:0032813 | tumor necrosis factor receptor superfamily binding | 3/10 | 45/22710 | 8.64e-07 | 5.10e-05 | 1.00e-05 |
| MF | GO:0070513 | death domain binding | 2/10 | 10/22710 | 7.84e-06 | 2.31e-04 | 4.54e-05 |
| MF | GO:0004709 | MAP kinase kinase kinase activity | 2/10 | 26/22710 | 5.64e-05 | 0.001 | 2.04e-04 |
| MF | GO:0043027 | cysteine-type endopeptidase inhibitor activity involved in apoptotic process | 2/10 | 29/22710 | 7.04e-05 | 0.001 | 2.04e-04 |
| MF | GO:0005164 | tumor necrosis factor receptor binding | 2/10 | 33/22710 | 9.15e-05 | 0.001 | 2.12e-04 |
| KEGG | mmu04668 | TNF signaling pathway | 8/10 | 113/8910 | 2.30e-14 | 1.56e-12 | 5.56e-13 |
| KEGG | mmu04210 | Apoptosis | 7/10 | 136/8910 | 1.91e-11 | 6.49e-10 | 2.31e-10 |
| KEGG | mmu04064 | NF-kappa B signaling pathway | 6/10 | 110/8910 | 6.23e-10 | 1.41e-08 | 5.03e-09 |
| KEGG | mmu04920 | Adipocytokine signaling pathway | 5/10 | 71/8910 | 6.81e-09 | 1.16e-07 | 4.12e-08 |
| KEGG | mmu04217 | Necroptosis | 6/10 | 174/8910 | 1.00e-08 | 1.36e-07 | 4.85e-08 |

**Table S5 GO and pathway enrichmenta table with Gene counts of 6 DEGs**

| ONTOLOGY | ID | Description | GeneRatio | BgRatio | pvalue | p.adjust | qvalue |
| --- | --- | --- | --- | --- | --- | --- | --- |
| BP | GO:0010039 | response to iron ion | 2/6 | 20/23210 | 1.06e-05 | 0.002 | 5.78e-04 |
| BP | GO:0001894 | tissue homeostasis | 2/6 | 232/23210 | 0.001 | 0.047 | 0.016 |
| BP | GO:0010038 | response to metal ion | 2/6 | 269/23210 | 0.002 | 0.047 | 0.016 |
| BP | GO:0070444 | oligodendrocyte progenitor proliferation | 1/6 | 12/23210 | 0.003 | 0.047 | 0.016 |
| BP | GO:0070445 | regulation of oligodendrocyte progenitor proliferation | 1/6 | 12/23210 | 0.003 | 0.047 | 0.016 |
| CC | GO:0051286 | cell tip | 1/6 | 10/23436 | 0.003 | 0.062 | 0.020 |
| CC | GO:0062023 | collagen-containing extracellular matrix | 2/6 | 359/23436 | 0.003 | 0.062 | 0.020 |
| CC | GO:0005614 | interstitial matrix | 1/6 | 17/23436 | 0.004 | 0.062 | 0.020 |
| CC | GO:0031012 | extracellular matrix | 2/6 | 475/23436 | 0.006 | 0.063 | 0.020 |
| CC | GO:0009925 | basal plasma membrane | 1/6 | 43/23436 | 0.011 | 0.070 | 0.022 |
| MF | GO:0008199 | ferric iron binding | 1/6 | 18/22710 | 0.005 | 0.026 | 0.011 |
| MF | GO:0016722 | oxidoreductase activity, oxidizing metal ions | 1/6 | 26/22710 | 0.007 | 0.026 | 0.011 |
| MF | GO:0008198 | ferrous iron binding | 1/6 | 33/22710 | 0.009 | 0.026 | 0.011 |
| MF | GO:0046915 | transition metal ion transmembrane transporter activity | 1/6 | 35/22710 | 0.009 | 0.026 | 0.011 |
| MF | GO:0030020 | extracellular matrix structural constituent conferring tensile strength | 1/6 | 38/22710 | 0.010 | 0.026 | 0.011 |
| KEGG | mmu04978 | Mineral absorption | 2/4 | 53/8910 | 2.07e-04 | 0.001 | 4.35e-04 |
| KEGG | mmu04216 | Ferroptosis | 1/4 | 40/8910 | 0.018 | 0.045 | 0.019 |
| KEGG | mmu04974 | Protein digestion and absorption | 1/4 | 108/8910 | 0.048 | 0.063 | 0.026 |
| KEGG | mmu04066 | HIF-1 signaling pathway | 1/4 | 114/8910 | 0.050 | 0.063 | 0.026 |
| KEGG | mmu04514 | Cell adhesion molecules | 1/4 | 175/8910 | 0.076 | 0.076 | 0.032 |

**Figure S1 miRNA-hub gene regulatory network**


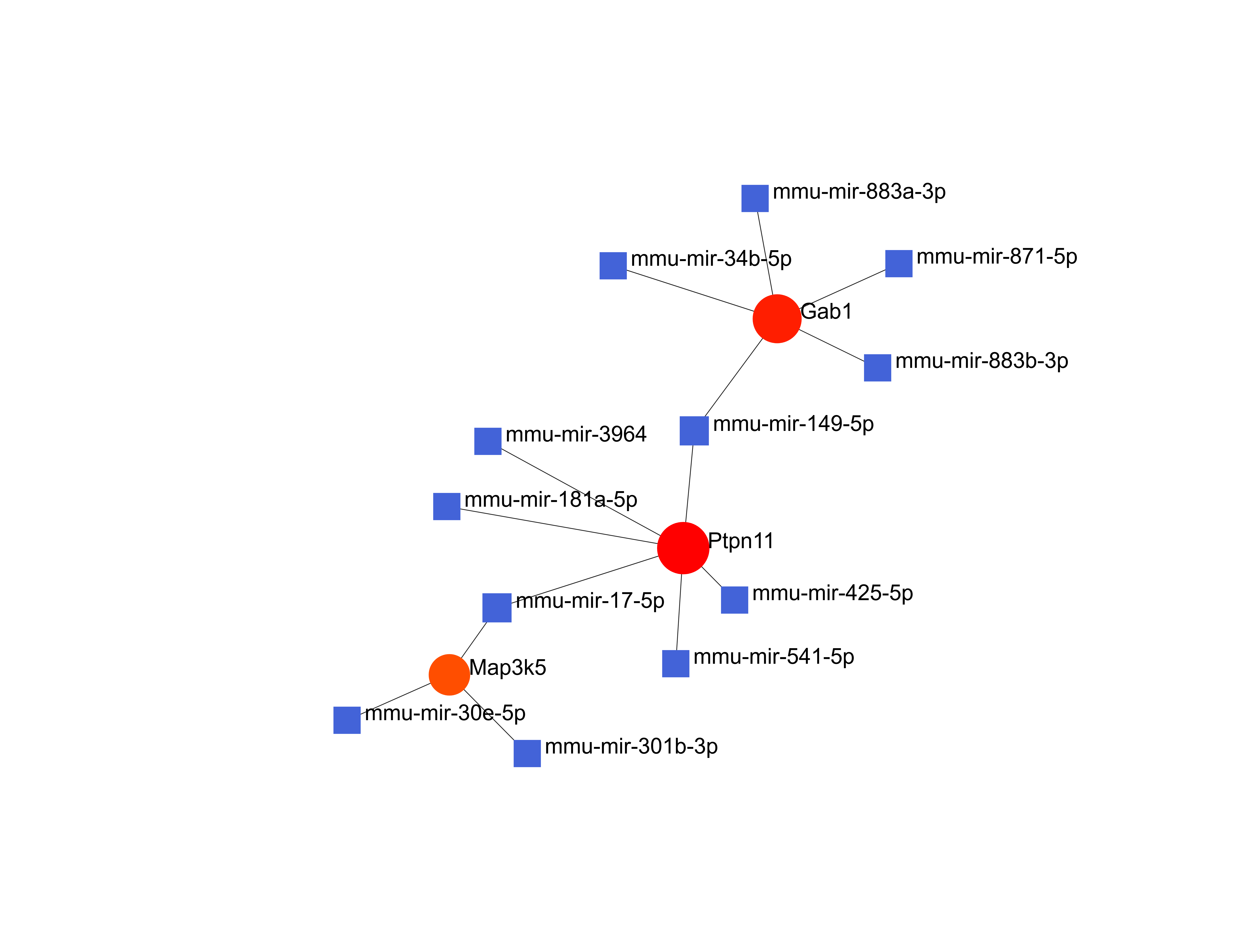


**Figure S2 TF-hub gene regulatory network**


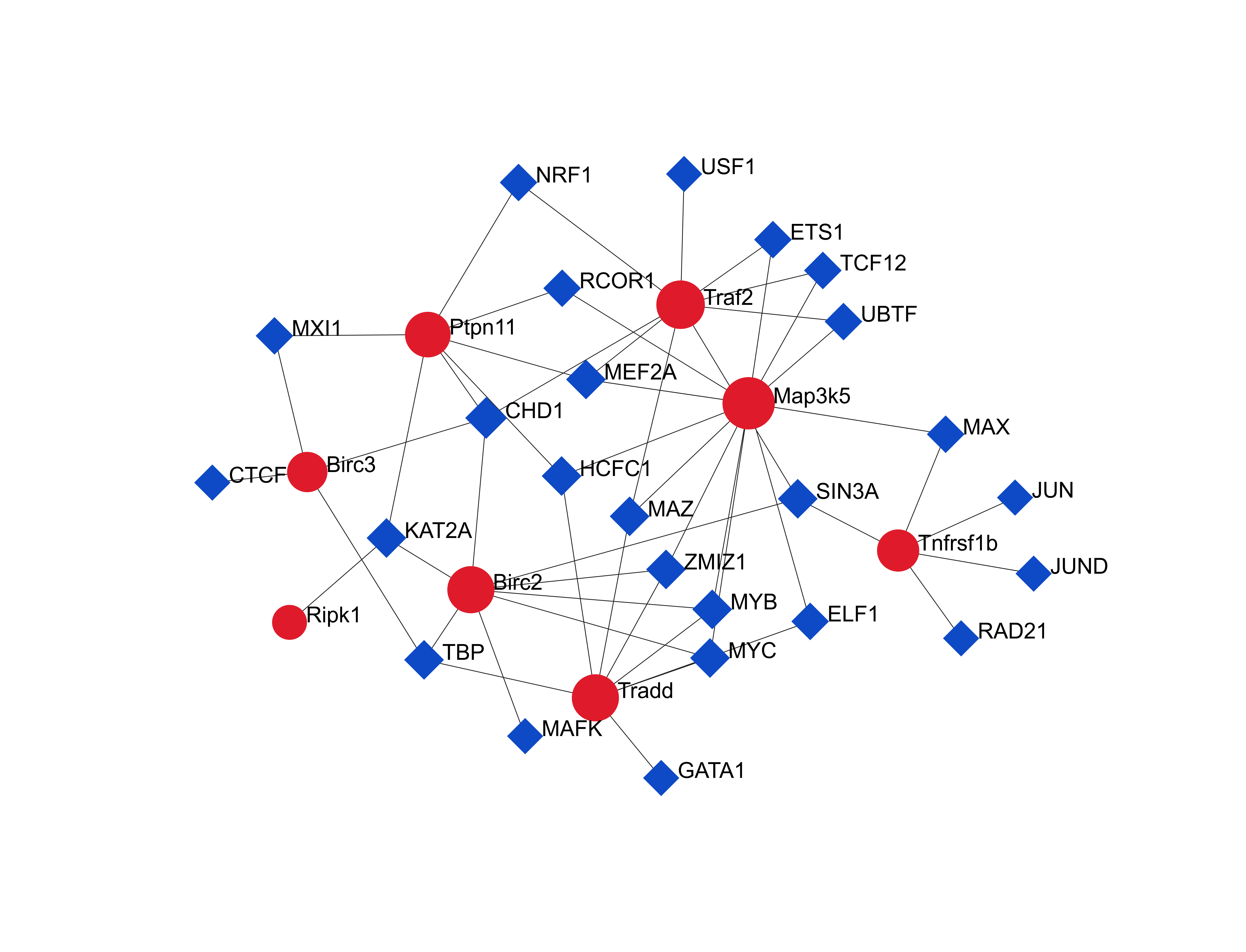

Supplement: Supplementary file 1 — Supplementary Information. [file 41598_2022_16314_MOESM1_ESM.doc]
